# Dynamics of glia and neurons regulate homeostatic rest, sleep and feeding behavior in *Drosophila*

**DOI:** 10.1038/s41593-025-01942-1

**Published:** 2025-04-21

**Authors:** Andres Flores-Valle, Ivan Vishniakou, Johannes D. Seelig

**Affiliations:** Max Planck Institute for Neurobiology of Behavior – caesar (MPINB), Bonn, Germany

**Keywords:** Sleep, Astrocyte, Microglia, Neural circuits, Feeding behaviour

## Abstract

Homeostatic processes, including sleep, are critical for brain function. Here we identify astrocyte-like glia (or astrocytes, AL) and ensheathing glia (EG), the two major classes of glia that arborize inside the brain, as brain-wide, locally acting homeostats for the short, naturally occurring rest and sleep bouts of *Drosophila*, and show that a subset of neurons in the fan-shaped body encodes feeding homeostasis. We show that the metabolic gas carbon dioxide, changes in pH and behavioral activity all induce long-lasting calcium responses in EG and AL, and that calcium levels in both glia types show circadian modulation. The homeostatic dynamics of these glia can be modeled based on behavior. Additionally, local optogenetic activation of AL or EG is sufficient to induce rest. Together, these results suggest that glial calcium levels are homeostatic controllers of metabolic activity, thus establishing a link between metabolism, rest and sleep.

## Abstract (web summary)

Animals alternate between active periods and periods of rest or sleep. This study in fruit flies points to brain metabolism as a cause for this and shows that a network of glial cells interacting with neurons links brain function with the need for rest and sleep.

## Main

Homeostatic processes in the brain, which often occur during sleep, are important for the reconfiguration of networks for learning and memory, for metabolism, for the removal of waste products, for the regulation of ion concentrations, and in response to disease^1–4^. Glia play a role in many of these processes^5^. Of the various classes of *Drosophila* glia^6,7^, astrocyte-like glia (AL) and ensheathing glia (EG) arborize inside the neuropil, numbering around 4,000 and 5,000 cells^6^, respectively, compared to about 130,000 neurons. Both AL and EG contribute to circadian and homeostatic sleep regulation^5,8,9^.

Bouts of rest and sleep in *Drosophila*^10–13^ are distributed throughout the day and night with a mean duration of only around 20 min^13–15^. Sleep is typically defined as bouts of rest that last longer than 5 min. The role of these short bouts of rest and sleep remains little understood, as typical approaches for investigating sleep homeostasis in the fly rely on many hours of sleep deprivation. Unlike in mice, where calcium imaging has linked glial activity to sleep–wake cycles^16^, the dynamics of glia and of most sleep-related neurons have yet to be described in behaving flies.

Here, we use two-photon calcium imaging over multiple days in head-fixed flies navigating in virtual reality (VR), to investigate the dynamics of EG, AL and sleep-related neurons in the central complex (CX)^9,17,18^. We describe circadian calcium dynamics and homeostatic dynamics for rest, sleep and feeding behavior. We further establish a link between the calcium-dependent regulation of carbon dioxide and pH and the subdivision of fly behavior into bouts of activity and rest or sleep.

## Short, spontaneous sleep bouts set timescale of homeostasis

**Fig. 1:**
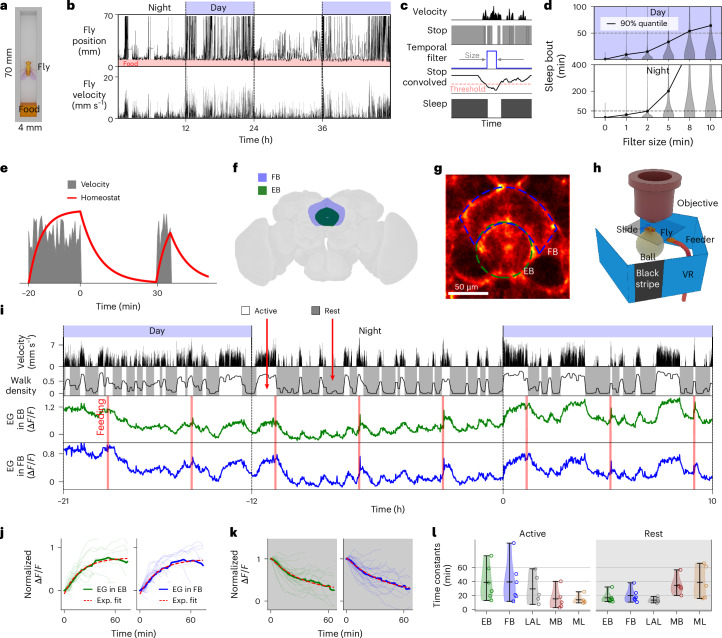
Behavior in freely moving flies and long-term calcium imaging in EG during behavior. **a**, Position of flies walking in chamber with food is tracked with a camera. **b**, Position along chamber and velocity of fly (2 days). **c**, Velocity (first row) is thresholded to find bouts of immobility (‘stop’, second row). Temporal filter (third row) convolved with fly stop state (fourth row) and thresholding to characterize sleep (fifth row). **d**, Distribution of sleep bouts for 15 flies during day and night depending on filter size. **e**, Schematic of sleep homeostat integrating behavior or time awake (rising, with nonzero velocity, gray) and time sleeping (decreasing, zero velocity). **f**, EB and FB. **g**, EG express jGCaMP8m and regions of interest (ROIs) in the EB (green) and the FB (blue). **h**, Setup for long-term imaging using microscopy: a fly is glued to a glass slide and navigates on an air-supported ball in a VR setup (Methods). **i**, Long-term recording of a fly over 31 h. Top row: day/night cycle in VR. Second row from top: absolute ball velocity. Third row: walk density (low-pass filtering with a cutoff period of 6 min of ‘walk’; walk: 1 if fly has nonzero velocity in 1-s bins, 0 otherwise). Filtering leads to epochs with mixture of states; see model-based analysis for separation of behavioral states. Active and rest states (walk density above or below threshold; Methods). Fourth and fifth rows: calcium activity in the EB and FB EG. Vertical red lines indicate feeding. **j**, Traces of increasing normalized calcium activity (Methods) during active states (thin lines, *n* = 30 traces); average (thick line) and exponential (red) fit for EB (left) and FB (right). **k**, As in **j** but during rest or sleep state (*n* = 25 traces), fitted with exponentials (red). **l**, Time constants and violin plots for exponential fitting during active (white region) and rest or sleep states (gray region) for EB, FB, LAL, MBs and ML show the distribution of time constants for six flies in the EB and FB, five flies in LAL and five flies in MBs and ML (circles). Short horizontal black lines in violin plots show maximum, mean and minimum values in descending order.

We confirmed that the short duration of a fly’s rest and sleep bouts^10,11,14,15^ is not the result of minor movements that only briefly interrupt overall longer bouts. We tracked flies walking in a rectangular chamber with a camera (Fig. 1a,b, Supplementary Fig. 1a,b and Methods), removed shorter periods of movement of varying lengths between consecutive bouts of immobility (Fig. 1c) and computed the resulting bout distribution. Even when filtering out movement periods of 2 min, 90% of bouts of immobility were still shorter than 50 min during the day and night (Fig. 1d), and even shorter bouts of immobility were observed in constant darkness (Supplementary Fig. 1c), consistent with previous results^14,15^.

A sleep homeostat needs to keep track of time spent awake and asleep^4,19,20^. This is typically modeled with two single exponential functions approaching an upper threshold for tracking time spent awake and a lower threshold for measuring time spent sleeping (Fig. 1e). The time constants of the two exponentials determine average wake and sleep bout durations^19,20^. Thus, based on behavior, the time constants of a brain signal that keeps track of rest or sleep and wake bouts in the fly are expected to lie in the range of several minutes or at most tens of minutes.

Although fly sleep is typically defined as at least 5 min of immobility, the arousal threshold increases already in the first 30 s to 1 min after the onset of immobility^12,21^ (also called ‘early sleep’)^22^. Additionally, a large fraction of immobility bouts is shorter than 5 min (Supplementary Fig. 2), and short bouts of inactivity of a maximum of 20 s are sufficient for long-term survival^23^. Further, the transition from rest to sleep is difficult to observe in a single fly and, as in mice^24^, could involve continuous and discrete processes. Therefore, in most cases we did not distinguish between immobility bouts shorter and longer than 5 min, or between rest and sleep; the discussion of homeostatic processes below applies to combined bouts of rest and sleep.

## EG and AL show homeostatic and circadian activity

Both AL and EG are important for sleep^5,7,9,25–28^ but whether and how these different glia types contribute to the short bouts of rest and sleep in the fly is not known. EG form diffusion barriers around different brain compartments^6,7^. Such a structural arrangement could serve to monitor the accumulation of metabolites and, therefore, to integrate neural activity in brain compartments, as would be expected of a sleep homeostat^20^. AL, which tile the brain more homogeneously^6^, could sense neural activity more locally^29^.

**Extended Data Fig. 1:**
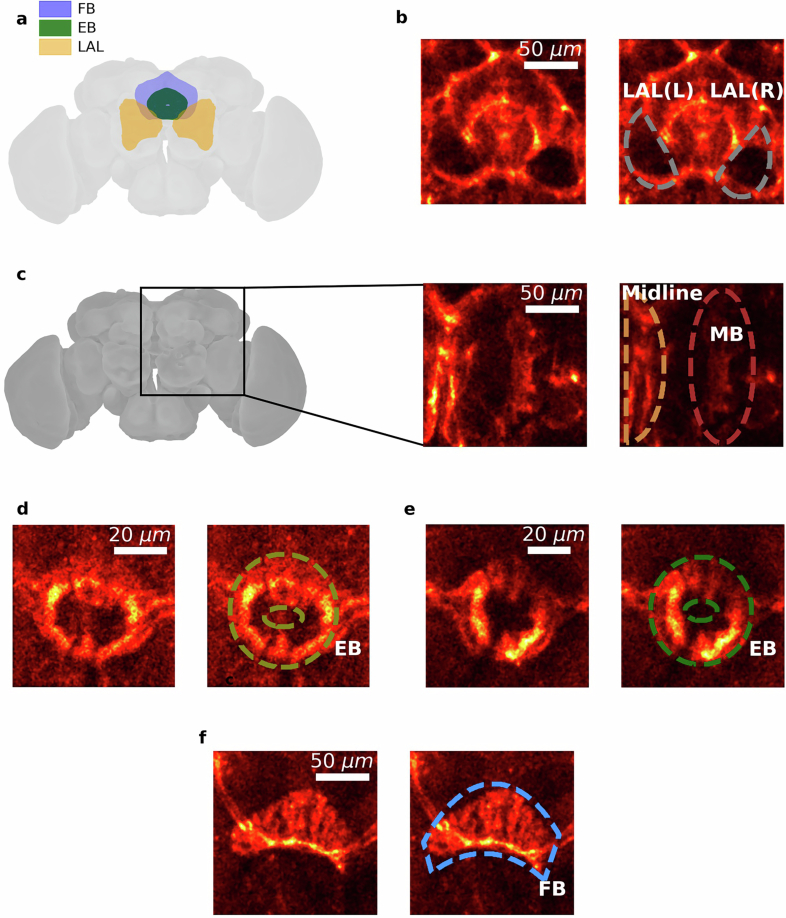
Regions of interests (ROIs) for recordings of EG in LAL, MB, and midline, as well as of R5 and dFB neurons. **a** Schematic of EB, FB, and left and right LAL in the fly brain. **b** Left side: EG in the central complex and LAL. On the right, ROI along the right (R) and left (L) LAL. **c** Left side: Drosophila brain area where EG in MB and midline were recorded (center). Right side: ROI along MB (brown) and midline (dark orange). **d** Left side: R5 neurons labeled by 58H05-GAL4. Right side: ROI along the ring-shaped projections of R5 neurons for calculating fluorescence changes. **e** Same as in a, but for R5 neurons labeled by 88F06-GAL4. **f** Same as a and b, but for dFB neurons labeled by 23E10-GAL4.

To investigate calcium dynamics of EG and AL in behaving animals, we used two-photon calcium imaging. In these experiments, flies walked on an air-supported ball in a VR setup in closed loop with a single dark stripe on a bright background during the day and in darkness during the night (12-h light–dark cycle; Methods). This closed-loop paradigm is frequently used for tethered fly behavior and provided a circadian light–dark cycle. We first expressed the calcium indicator jGCaMP8m in EG using the GAL4 line R56F03 (refs. ^6,25^) and imaged different compartments of the central brain, for example, the ellipsoid body (EB) and the fan-shaped body (FB; Fig. 1f,g and Methods). We fed flies in the imaging setup using an automated feeder (Fig. 1h) every 4 h (Methods), which induced epochs of continuous walking activity (often before feeding) or rest and sleep (often after feeding; Fig. 1i). Under these conditions, EG in different brain areas, including the EB, the FB, the lateral accessory lobe (LAL; Extended Data Fig. 1a,b), mushroom bodies (MBs) and the midline (ML; Extended Data Fig. 1c), showed pronounced calcium fluctuations (Fig. 1i and Supplementary Figs. 3–5 and Videos 1–6 for EB and FB).

In a first analysis, we distinguished two behavioral states based on the fly’s walking activity: ‘walk’ (fly is walking) and ‘stop’ (fly is standing still as assessed based on ball velocity; second row in Fig. 1i, and Methods). We then selected epochs of at least 10 min during which the fly was walking most of the time (active; Fig. 1i) or immobile most of the time (rest or sleep; Fig. 1i) by thresholding the ‘walk density’, obtained by low-pass filtering of ‘walk’. Low-pass filtering results in a mixture of behavioral states, where epochs of immobility contain a fraction of walking and vice versa. A complete separation of behavioral states is achieved with the model-based analysis described below.

**Extended Data Fig. 2:**
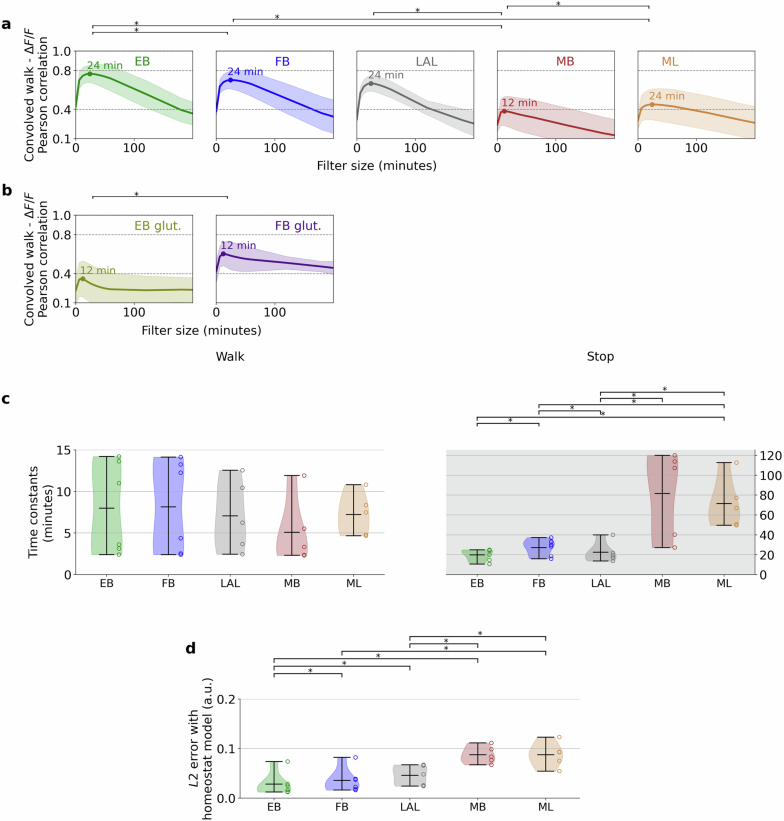
Comparison between EG activity in different neuropils and glutamate activity in the central complex. **a** Pearson correlation between convolved ‘walk’ and glia calcium activity in different neuropils for different filter sizes (x-axis, see Extended Data Fig. 7h for concept, and section ‘Correlation between ‘convolved walk’ and calcium’ in Methods). Thick-colored lines represent average of correlation curves, while light-colored areas represent standard deviation for all flies (n= 6 flies for EB and FB, and n= 5 flies for LAL, MB, and midline). Colored numbers indicate filter size at maximum correlation value in min. **b** Same as a, but for glutamate activity in EB and FB (n= 5 flies). **c** Distributions of time constants for ‘stop’ and ‘walk’ states from model fitting (n= 6 flies in EB and FB, and n= 5 flies in LAL, MB, and midline). **d** **L**2 error between normalized fluorescence and the corresponding fitted homeostat 2-state model (n= 6 flies in EB and FB, and n= 5 flies in LAL, MB, and midline). In **c**-**d**, short horizontal black lines: maximum, mean, minimum in descending order. Asterisks in each panel represent statistical significance using two-sided t-tests (p < 0.05). All p-values were corrected using Benjamini-Hochberg procedure (Supplementary Data 1).

Fluorescence traces were averaged over the selected active and rest or sleep epochs (Fig. 1j,k and Supplementary Figs. 6–8). The averaged fluorescence could be fitted with exponentials (Methods), the expected time course of sleep homeostasis^19^ (Fig. 1j,k and Supplementary Figs. 6–8). We verified that plateaus were not due to indicator saturation (Methods and Supplementary Fig. 9). The resulting rise and decay time constants of the exponential fits are shown in Fig. 1l for six flies for the EB and FB, five flies for LAL and five flies for MBs and ML, and lie in the range expected based on behavior (Fig. 1d,e). Dynamics in the EB, FB and LAL were similar, whereas MBs and the ML showed faster dynamics during active epochs (but lower correlation with walking behavior; Extended Data Fig. 2a and Methods), and slower dynamics during rest and sleep epochs (see also Extended Data Fig. 2c, Supplementary Figs. 3–8, 10 and 11 for MBs, and ML, and Supplementary Results 1.1). EG also showed fluctuations at circadian timescales, with overall higher activity during day than night (Fig. 1i, see below).

**Fig. 2:**
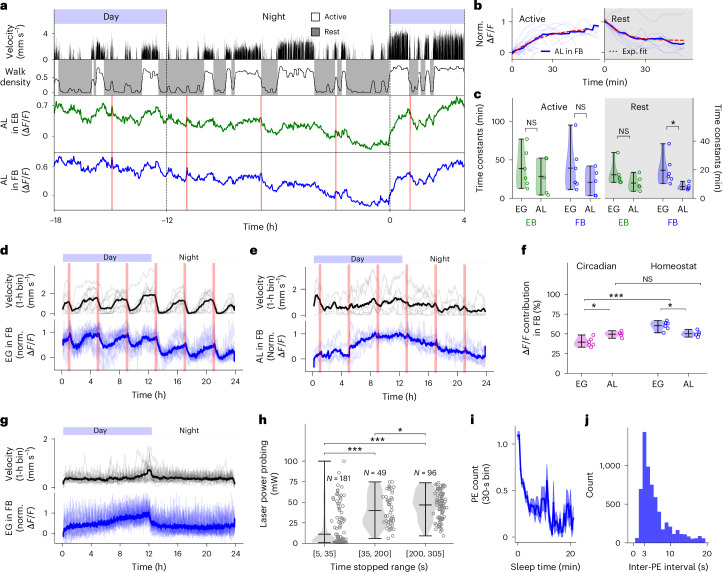
Calcium dynamics in AL, circadian activity, arousal threshold and proboscis extension during long-term imaging experiments. **a**, Recording of AL in the EB and FB for 22 h (similar to Fig. 1i). **b**, As in Fig. 1j,k but for AL in the FB (*n* = 22 and *n* = 12 traces for active and rest, respectively). **c**, Time constants of EG (6 flies) and AL (5 flies) in the EB and FB during active and rest behaviors (as in Fig. 1l; note different scales for active and rest) together with violin plots (see **h**). **d**, Average velocity (second row) and activity of EG in the FB (third row) over 24 h (*n* = 14 traces from 6 flies). Thin lines represent individual days for each fly, while thick lines represent the average. Red vertical lines show feeding times. **e**, As in **d** but for AL in the FB (*n* = 9 traces from 5 flies). **f**, Percentage of circadian (magenta) and homeostat (blue) contributions to the activity of EG (6 flies) and AL (5 flies) in the FB (Methods) and violin plots (see **h**). In **c** and **f**, statistical significance was assessed using a two-sided *t*-test (*P < 0.05 or ***P < 0.0005; for detailed statistical information, see Supplementary Data 1). **g**, Same as **d** but for experiments where flies were fed every 26 min (*n* = 19 traces from 5 flies). **h**, Laser power required to awaken the fly based on the previous durations of immobility during calcium imaging together with violin plots. Durations of immobility are grouped in three different time ranges; the number of probing trials is indicated at the top (for a total of 6 flies). Statistical significance was assessed using the two-sided Kolmogorov–Smirnov test (*P < 0.05 or ***P < 0.0005). *P* values were corrected using the Benjamini–Hochberg procedure. For detailed statistical information, see Supplementary Data 1. Circles denote individual data points; short horizontal black lines in **c**, **f** and **h** indicate the maximum, mean and minimum values in descending order. **i**, Proboscis extension (PE) count as a function of sleep time during long-term imaging experiments for 21 flies. Only rest or sleep bouts longer than 1 min of immobility were considered. The blue thick line shows the mean, and the blue region represents the s.e.m. **j**, Histogram of time between proboscis extension events during rest or sleep behavior during long-term imaging experiments (21 flies). NS, not significant.

We also investigated AL calcium dynamics. We expressed the calcium indicator jGCaMP8m in 86E01-GAL4 (ref. ^26^). AL showed calcium signals with stronger circadian modulation than EG (Fig. 2a) and also showed superimposed homeostatic fluctuations (Fig. 2a and Supplementary Fig. 12). Time constants for these modulations were computed as before (see below for a more detailed, model-based analysis), by separating epochs where flies were either mostly walking or at rest or sleeping (Fig. 2b, Supplementary Fig. 13 and Methods). The time constants for AL homeostatic signals resulting from this analysis were slightly faster than those of EG (Fig. 2c).

Circadian activity was weaker in EG than in AL (Fig. 2d–f and Methods). To better observe circadian oscillations in EG largely independent of behavior, we minimized walking activity by keeping flies well fed at all times. We fed the flies every 26 min (Fig. 1g and Supplementary Fig. 14) or 16 min (Supplementary Fig. 15)—a rate similar to the rate with which freely walking flies approached food (Supplementary Fig. 1d)—for 2 min using the automated feeder, without interrupting the experiments (Fig. 1h). The approaching feeder led to brief bouts of walking behavior at the time of feeding and corresponding walking-induced (as described in the previous section, as well as potentially due to startling the fly, see below) spikes of calcium activity (Fig. 2g and Supplementary Figs. 14 and 15) but little walking activity between feeding events. Under these conditions, calcium activity—both in individual flies and averaged across flies—slowly increased over the day and reset during the night, with a circadian pattern largely independent of behavior (Fig. 2g). Overall, both EG and AL show homeostatic fluctuations—with dynamics specific to behavior and brain area—and circadian fluctuations. Homeostatic fluctuations in EG were stronger than circadian fluctuations, and were stronger than homeostatic fluctuations in AL (Fig. 2f). Circadian fluctuations were stronger in AL than in EG (Fig. 2f).

## Flies show essential characteristics of sleep during imaging

**Extended Data Fig. 3:**
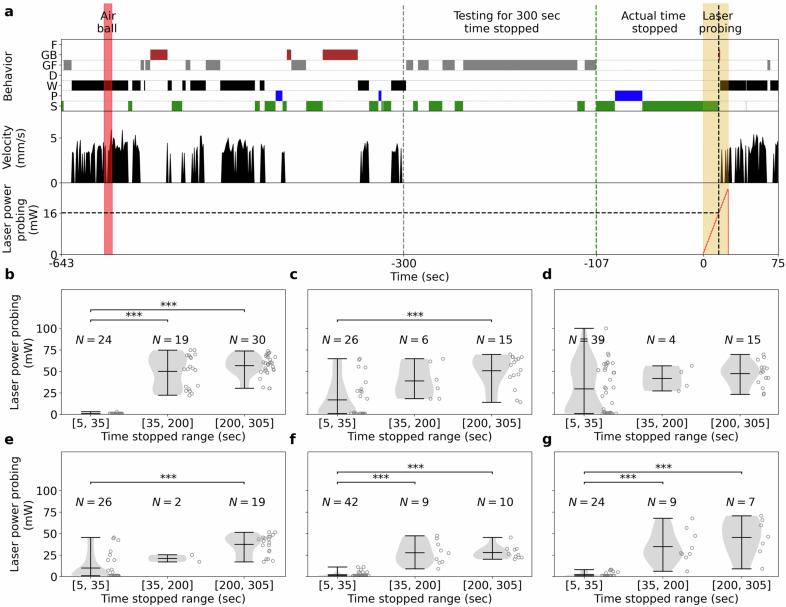
Arousal threshold increases with time of immobility during two-photon imaging as in freely moving animals. **a** Example of trial protocol for testing arousal threshold after 300 sec of immobility of a fly on the ball. First, the air stream supporting the ball is switched on and off repeatedly (1 s on, 1 s off, 3 times, red area) to ensure the fly is awake. The fly stops walking for 300 sec (fly velocity is zero in second row) and the laser (third row) starts probing the arousal threshold of the fly by increasing its power every 0.5 sec (orange area). The laser is switched off when the fly starts walking again (velocity larger than 0). Post processing: behavior classification (first row) is used to compute the actual time fly was immobile, without grooming (107 sec in this trial), as well as the actual laser power the fly reacts to by grooming, discomfort, or walking during laser probing (16 mW in this trial). **b-g** Distribution of laser powers at which fly wakes up for all trials for different durations of immobility for 6 flies (each panel is a different fly). Circles: individual data points; short horizontal black lines: maximum, mean, minimum in descending order. One, two, or three asterisks indicate statistical significance using two-sided t-tests (p < 0.05, p < 0.005, p < 0.0005, respectively, Supplementary Data 1.

The distributions of immobility bouts during long-term imaging were similar (during the day) and identical (during the night) to those of freely walking flies (Supplementary Fig. 2a,b and Methods). To determine whether longer epochs of immobility during long-term imaging experiments correspond to sleep, we tested whether the arousal threshold—the required stimulus strength to induce a behavioral response in resting flies—increased with the duration of immobility, as would be expected for sleep^12^. As a stimulus, we used an infrared (IR) laser beam pointed at the fly’s abdomen. A representative trial of threshold probing is shown in Extended Data Fig. 3a (see Methods and Supplementary Results 2.1 for details on how arousal thresholds were measured). Longer periods of immobility required higher laser power to trigger a behavioral response (Fig. 2h), indicating an increased arousal threshold—a feature of sleep also observed in non-head-fixed flies^10–12^ (see Extended Data Fig. 3b–g for individual flies). Proboscis extension, another sleep-related behavior^21,22^, was detected by classifying behavior using machine learning (Methods and Supplementary Fig. 16) and occurred more frequently at the beginning of rest or sleep bouts, as previously described, and also at similar frequencies (Fig. 2i,j)^22^; however, the distribution (Fig. 2i) shown here includes both bouts of rest and rest after feeding, during long-term imaging (Fig. 2i). Typical characteristics of sleep behavior observed in freely moving flies are thus preserved during long-term imaging, suggesting that brain-related mechanisms for sleep control will operate similarly under both conditions.

## EG integrate behavioral effort and show fast calcium spikes

Since EG show stronger homeostatic fluctuations of calcium activity than AL (Fig. 2f), we performed a more detailed analysis of the homeostatic features of these cells. Rest and sleep behavior often—but not always—followed feeding events (Figs. 1i and 2a and Supplementary Figs. 3–5), with flies increasingly walking more with time elapsed since feeding, and resting or sleeping more after feeding (as observed in freely walking flies^9^).

**Fig. 3:**
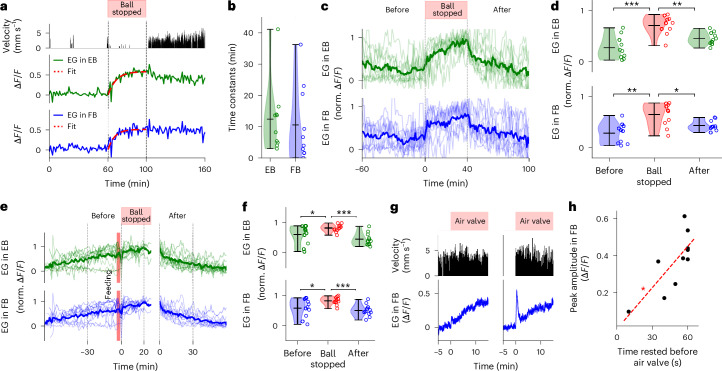
Calcium dynamics in EG increase due to effortful behavior, decrease during rest, not after feeding, and spike at rest to walking transitions. **a**, Calcium activity during effort with blocked treadmill ball. Second row: velocity of fly over time (zero while stopping the ball). Third and fourth rows: EG activity and exponential fit (red) in the EB and FB, respectively. **b**, Time constants of exponential fits together with violin plots (see **f**) while ball was blocked for *n* = 10 trials in 5 flies. **c**, Normalized fluorescence traces of EG activity in the EB and FB before, during and after ball was blocked (top row). Second and third rows: single trials (*n* = 10 trials in 5 flies, thin lines) and average (thick lines) in the EB and FB, respectively. **d**, Normalized fluorescence levels from **c** before, during and after ball blocking (60, 40 and 60 min average, for *n* = 10 trials in 5 flies) and violin plots (see **f**), respectively. **e**, Calcium activity during effortful behavior after feeding. Normalized fluorescence traces of EG activity in the EB and FB before feeding, after feeding while the ball was blocked, and after releasing the ball (for *n* = 11 trials in 5 flies). **f**, Normalized fluorescence levels and violin plots from **e** before feeding, after feeding while blocking the ball, and after releasing the ball (30, 26 and 30 min average, for *n* = 11 trials in 5 flies). In **d** and **f**, statistical significance was assessed using a two-sided *t*-test (*P < 0.05, **P < 0.005 and ***P < 0.0005; for detailed statistical information, see Supplementary Data 1). Circles denote individual data points. Short horizontal black lines in **b**, **d** and **f** denote the maximum, mean and minimum values in descending order. **g**, Velocity (second row) and EG activity in the FB (bottom row) while the air valve of the ball is switched on and off every second (top row, red) for two trials without and with a calcium peak at the onset of mechanical stimulation (left and right, respectively). **h**, EG peak amplitude in the FB at the onset of mechanical stimulation as a function of time rested, shown 60 s before the onset of ball stimulation (longer intervals are included at 60 s, for *n* = 10 trials in 7 flies). Red line shows linear fit. Statistical significance of the fit was assessed using a Pearson correlation (*P < 0.05; for detailed statistical information, see Supplementary Data 1).

We then assessed whether the increase in calcium activity in EG observed during walking (Fig. 1i,j) was specifically triggered by walking, or whether other effortful behaviors cause similar activity changes. We immobilized the ball for epochs of 40 min while the fly was in the VR setup, and recorded behavior and EG activity in the EB and FB as before. Movement of the ball was blocked by holding it down with the tip of a remote-controlled brush in such a way that the fly continually pushed or pulled (Methods). As seen in the resulting fluorescence traces (Fig. 3a and Supplementary Fig. 17), this led to a steady increase in or saturation of calcium levels similar to the dynamics observed due to walking activity but with slightly faster time constants (Fig. 3b and Fig. 1j,l). This shows that EG calcium activity increases not only due to coordinated walking but also due to other effortful behaviors (Fig. 3c,d), as similarly observed in zebrafish^30^.

Next, we verified that feeding was not the cause for the decay of calcium activity in those instances where flies were resting or sleeping after feeding. We induced sleep deprivation in flies immediately after feeding by inhibiting ball movement as above (Fig. 3e and Supplementary Fig. 18). This not only suppressed the resetting of calcium levels, but also slightly increased calcium signals after feeding (Fig. 3e,f), demonstrating that rest or sleep, and not feeding, underlies the decay of calcium levels after feeding.

EG integrate activity over long timescales (tens of minutes), as indicated by the time constants (Fig. 1l, and see below for time constants from model fitting). Modulations of fluorescence signals were also observed at the timescale of 1 min when comparing changes in walking velocity with changes in high-pass-filtered calcium signals in the EB and FB (*ΔF/F*; Supplementary Fig. 19a–i and Methods). Thus, the accumulation of such faster fluctuations could lead to the integrated activity observed over longer timescales.

To investigate the transition from immobility to walking in more detail, we imaged calcium dynamics at higher time resolution (Fig. 3g and Methods). We selected bouts where flies were immobile for several minutes, and then forced them to walk by interrupting the airstream supporting the treadmill ball with an automated valve periodically every second. Under these conditions, EG calcium activity showed a fast peak at walking onset, as similarly observed in mice^31,32^, followed by the slower integrating response described above (Fig. 3g). Such calcium peaks were not observed when the fly was already walking before the onset of treadmill perturbations (Fig. 3g). The peak amplitude increased when flies were immobile for longer periods before the onset of forced walking (Fig. 3h) with a linear relationship for bouts of immobility for up to 60 s. Potentially related signals were observed in a recent paper following electric shock in MBs^33^. These findings suggest that also faster fluctuations of calcium, and not only overall homeostatic trends, are relevant for behavior.

## Sleep deprivation saturates EG activity

**Fig. 4:**
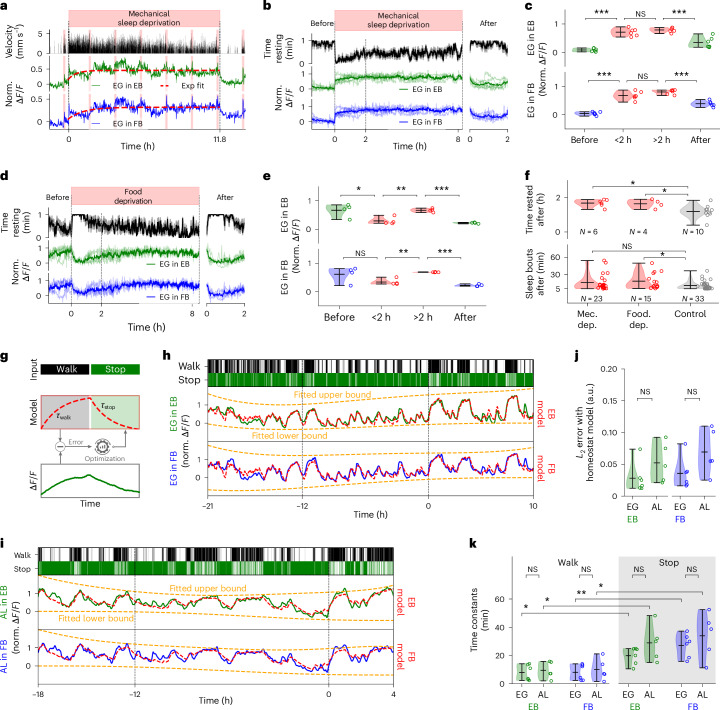
EG activity during sleep deprivation and homeostat model. **a**, Behavior and calcium activity in EB and FB EG under mechanical sleep deprivation. Top row: time of deprivation. Second row: fly velocity. Third and fourth rows: activity of EG (exponential fit for visualization, red). Red lines indicate feeding. **b**, Sleep deprivation for *n* = 6 trials and *n* = 5 flies (including fly in **a**). Top row as in **a**. Second row: average time resting, 1-min bins. Third and fourth rows: normalized fluorescence of EG per fly (thin) and average (thick line, at least 2 flies) in the EB (green) and the FB (blue). Times of sleep deprivation varied. Flies were fed during experiments (Supplementary Fig. 20). **c**, Average fluorescence from **b** and violin plots (see **k**) before (over 1.5 h), during the first 2 h, between 2 h and 8 h of, and after, sleep deprivation (2 h, *n* = 6 trials, *n* = 5 flies). **d**, As in **b** but during starvation-induced sleep deprivation; *n* = 4 trials in different flies. Top row: time (red) between feeding (Supplementary Fig. 20b). **e**, As in **c** for starvation (*n* = 4 trials). **f**, Top row: time rested over 2 h and violin plots (see **k**), after mechanical or starvation-induced deprivation, and controls (*n* = 10 trials, *n* = 6 flies). Bottom row: sleep bouts (>5 min) over 2 h after mechanical or starvation-induced deprivation, and controls. Asterisks indicate significance (*t*-test, *P* < 0.05, **c**, **e**, top row in **f**, Kolmogorov–Smirnov bottom row of **f**; *P* values in **f** corrected with Benjamini–Hochberg; for detailed statistical information, see Supplementary Data 1). **g**, Model fitting, behavior (walk, stop) is integrated with time constants and fitted to EG activity. **h**, Band-pass-filtered (0.5 to 12 h) calcium in EG EB and FB. Top row: ‘walk’ state (velocity > 0 in 1-s bins). Second and third rows: activity in the EB and FB. Red line, fitted two-state model; orange lines, fitted corrections (Methods). **i**, As in **h** but for AL in the EB and FB. **j**, *L*_2_ error between model and EG calcium with violin plots (see **k**; *n* = 6 flies) and AL (*n* = 5 flies) in the EB (left) and the FB (right). **k**, Time constants for ‘stop’ and ‘walk’ states from model fitting and violin plots for EG (*n* = 6 flies) and AL (*n* = 5 flies) in the EB (green) and the FB (blue). In **c**, **e**, **f**, **j** and **k**, statistical significance was assessed using a two-sided *t*-test (*P < 0.05, **P < 0.005, ***P < 0.0005; for detailed statistical information, see Supplementary Data 1). Circles and short horizontal black lines denote individual data points, and maximum, mean and minimum values in descending order, respectively. See Methods and Supplementary Results 1.4 for models with three and seven behavioral states. a.u., arbitrary units.

A signature of a sleep homeostat is that it saturates and plateaus under sleep deprivation^19^. To induce sleep deprivation in flies during long-term imaging, we periodically opened and closed the airstream supporting the ball at 1-s intervals for 6 s every 20 s (similar to sleep deprivation induced in freely walking flies^17,23^), which induced short bouts of fast walking (at least every 20 s). Calcium activity saturated in the EB and FB (Fig. 4a,b and Supplementary Fig. 20a) within 2 h and plateaued (Fig. 4c), with occasional fluctuations around the saturation level (indicated by an exponential fit; Fig. 4a). This experiment again confirmed that behavioral activity—here walking—prevents resetting of calcium levels after feeding, similarly to the experiment in Fig. 3e (see Fig. 4a and Supplementary Fig. 20a for feeding events during sleep deprivation).

We also used food deprivation to induce sleep deprivation in flies^9^. Single trials and average activity of four flies that were continuously walking for at least 3 h following food deprivation with fluorescence traces normalized for comparison are shown in Fig. 4d (see Supplementary Fig. 20b for individual trials). Starvation-induced walking produced a slow increase of EG calcium activity (Fig. 4d), and, similarly to mechanical sleep deprivation, calcium activity saturated after 2 h (Fig. 4e).

Sleep deprivation for extended periods of time leads to rebound sleep in flies^9–11^. Twelve hours of sleep deprivation induced without strong mechanical perturbation results in <1 h of mean rebound sleep^23^. After termination of mechanically or food-induced sleep deprivation, flies became less active (Fig. 4b,d), and EG activity returned to lower levels. Flies displayed an average of 1.68 h and 1.64 h of immobility in the 2 h after termination of mechanical and starvation-induced sleep deprivation, respectively, compared to 1.19 h in control flies (Fig. 4f and Methods)—a difference of around 30 min. Additionally, food deprivation resulted in more sleep bouts of >5-min duration (Fig. 4f). Food deprivation does not lead to homeostatic responses^34^, but camera-based methods might be required to detect the smaller effects we observe here^14,23^. Therefore, EG show the characteristics of a sleep homeostat during extended sleep deprivation, including rebound sleep in the range expected based on EG calcium time constants.

## Homeostat model describes glial calcium activity

Homeostatic activity can be modeled as exponentially decaying during sleep and exponentially approaching a saturation level during wakefulness (Fig. 1e)^19^. Therefore, we quantified whether EG calcium levels integrate wake and immobility according to such homeostatic dynamics. We did not distinguish between rest and sleep (more than 5 min of immobility; see next section); the resulting homeostat therefore resets during rest and sleep with the same time constant. We fitted a differential equation with two time constants for charging and resetting of the homeostat, respectively, to EG calcium activity dependent on behavioral state (two-state model; Methods). As illustrated in Fig. 4g, the behavioral states, here ‘walk’ and ‘stop’, are passed to the homeostat model and differentially increase or decrease activity with time constants characterizing the exponential dynamics. After fitting, the homeostat model describes EG activity over the time course of the experiments based on behavior (31 h in Fig. 4h, see also Supplementary Figs. 21, 22 and 23). Similarly, AL calcium dynamics can be fitted with a homeostat model (Fig. 4i and Supplementary Fig. 24, see Fig. 4j for statistical difference in *L*_2_ error between the model fits and activity).

The resulting rise and decay time constants of EG and AL for six and five flies, respectively, in the EB and FB are shown in Fig. 4k (see Extended Data Fig. 2c for EG in LAL, MBs and ML). The homeostat model fitted activity in the EB, FB and LAL regions better than activity in MBs and ML (Extended Data Fig. 2d). Time constants in Fig. 2c were more similar between rise and decay than those obtained by model fitting (Fig. 4k and Extended Data Fig. 2c). This was because active and rest states in Figs. 1j–l and 2b,c were determined using low-pass filtering of walking activity (Figs. 1i and 2a), resulting in rest states that still contained a fraction of walking activity, and vice versa. Low-pass-filtered active states contained more brief periods of immobility, than vice versa, and therefore time constants of active states changed more strongly between the two approaches. Time constants obtained from model fitting were not different between AL and EG (Fig. 4k).

Resetting of the homeostat to below 2% from baseline occurs over about four time constants or 60 to 120 min for CX, or 80 to 200 min in MBs, a time span that covers a large fraction of the rest and sleep bouts observed in freely walking flies^10,14,15^ (Figs. 1d and 4k). The time constants were faster for charging than for resetting of the homeostat (Fig. 4k and Supplementary Fig. 25a), consistent with the observation that flies rest or sleep more than they are awake over a 24-h period^14,15^. The glia homeostat (that is, calcium levels) reset over time during both rest and sleep, and the arousal threshold increased with sleep duration (Fig. 2h), at least initially, as expected^12^. That the arousal threshold increases with the duration of rest or sleep could be attributed to a decrease of neural excitability^20^. Excitability is, therefore, expected to be highest at the end of wake bouts, when also homeostatic calcium levels are highest.

**Extended Data Fig. 4:**
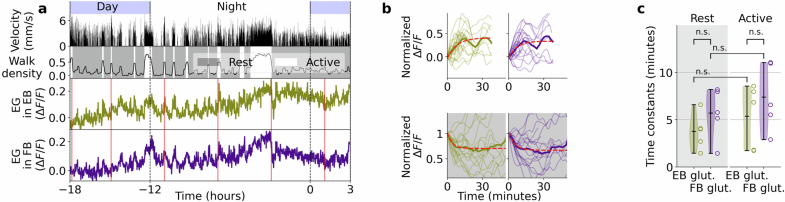
Ensheathing glia glutamate dynamics. **a** Glutamate long-term imaging in EB and FB. Similar to Fig. 1i for glutamate. Top row: day and night cycle in VR. Second row: fly velocity. Third row: walk density with rest (gray region) and active (white region). Fourth and fifth row: extracellular glutamate activity in EB and FB, respectively. Thick lines indicate band-pass filtered signal (0.1 h). **b** Top: single traces (n= 25) of increasing normalized glutamate activity (see Methods) during active state (thin lines), with average (thick line) and exponential fit (red) for EB and FB. Bottom: Same as the top panel but during rest state (n= 19). **c** Time constants for exponential fit during active (white region) and rest (gray region) for extracellular glutamate in EB and FB in n=5 flies. ROIs were selected similarly as for calcium imaging, see Fig. 1g. Circles: individual data points; short horizontal black lines: maximum, mean, and minimum values in descending order. No statistical significance across the time constants was observed using the two-sided t-test (p > 0.05, Supplementary Data 1). See also Supplementary Fig. S26.

As EG play a role in glutamate homeostasis^7^, we additionally tested whether calcium responses could result from accumulation of glutamate. However, time constants for glutamate were faster than those for calcium (Extended Data Figs. 2b and 4, Supplementary Figs. 26 and 27 and Supplementary Results 1.3), suggesting that glutamate accumulation is not the determining factor for EG calcium dynamics. In conclusion, this analysis shows that EG calcium dynamics can be modeled as a homeostat taking into account walking and rest behavior.

**Extended Data Fig. 5:**
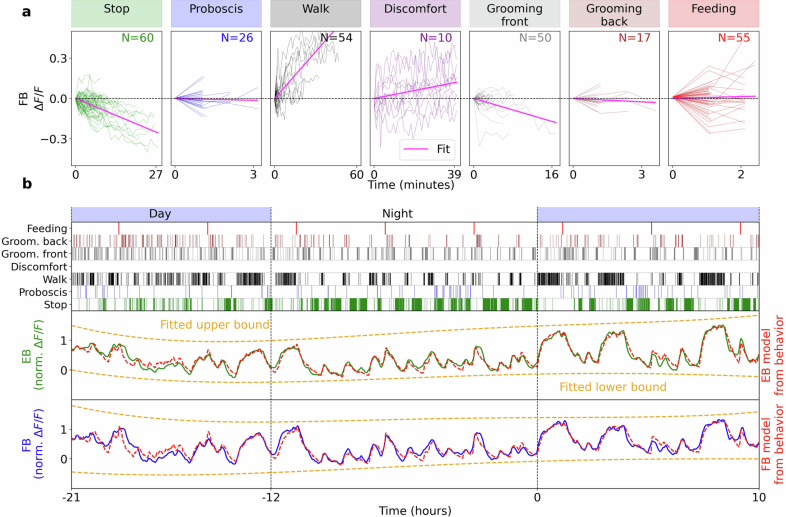
A homeostat model with 7 behavioral states (seven-state model) to describe EG activity. Fitting calcium activity with 7 behavioral states (seven-state model). **a** Determination of sign of weights of each behavior for model fitting. Fluorescence FB traces while fly is performing the respective behavior for at least two consecutive fluorescence recordings (2 min, combined across flies, see Methods). black shows linear fit, negative slope results in negative weight, and vice versa. Flies were often walking fast during feeding, likely resulting in increasing fluorescence. Note that the scale of y-axes differs between plots. **b** Model fitting with 7 states. Top: day-night cycle. Second row: classified fly behavior over time. Third row: band-pass filtered activity of glia in EB (green line); red line shows model fit, orange lines show fitted correction of fluorescence levels. Fourth row: same as third row for FB. ROIs are shown in Fig. 1g.

Different behaviors contribute differently to sleep need and are, therefore, expected to charge the homeostat with different time constants^2,9^. An exponential homeostat model with seven different behaviors, including proboscis extension and front or back grooming, improved model fitting (Methods, Supplementary Results 1.4, Supplementary Fig. 25, Supplementary Tables 5 and 6 and Extended Data Fig. 5).

To determine whether different time constants for rest and sleep could be inferred, we fitted a homeostat model with two independent decays to EG activity. Distinguishing between epochs with less and more than 5 min of immobility did not result in significantly different time constants between the two states (Supplementary Figs. 28 and 29 and Methods), also when defining a second state as starting only 5 min after the onset of immobility (but see also the next section).

**Extended Data Fig. 6:**
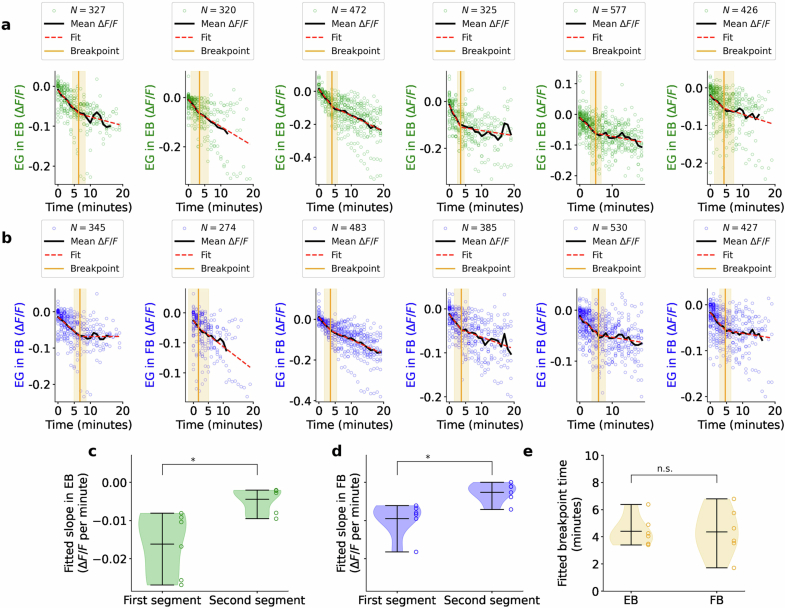
Two different states can be distinguished based on calcium reset velocity, with a transition around 5 minutes. Piecewise linear regression applied to calcium activity in ensheathing glia (EG) during periods of immobility. **a** Each panel shows calcium activity recorded in EB from a single fly (from Fig. 1i and Supplementary Fig. S3). Green dots (total number N shown at the top) represent the measured fluorescence during immobility bouts of at least 3 min, obtained from long-term calcium recordings (see Methods). The black line indicates the average fluorescence over time, while the red line shows the piecewise regression, consisting of two linear segments. The orange vertical line marks the fitted breakpoint between the two segments, and the shaded orange region represents the confidence interval for the breakpoint. **b** Same as a, but for FB. **c** and **d** show the fitted slopes of the two segments for EB and FB in panels **a** and **b**, respectively (from 6 flies), with asterisks indicating statistical significance (two-sided t-test, p-values < 0.05, Supplementary Data 1). **e** Fitted breakpoint times at which the regression transitions between segments for EB and FB (from 6 flies). The fitted breakpoints in EB and FB do not show a statistically significant difference (two-sided t-test, p-value > 0.05, Supplementary Data 1). Circles: individual data points. Short horizontal black lines in panels **c**-**e**: maximum, mean, and minimum values in descending order.

For an exponential model, the calcium change (or velocity) is time dependent: the decay is fastest at the beginning and slows down with time. We, therefore, assessed whether rest and sleep could be distinguished based on calcium velocity. An approximation of calcium decays during immobility with two linear models separated by optimization of a single breakpoint yields a change of state at around 4–6 min (Extended Data Fig. 6 and Methods). Therefore, two different states can be distinguished based on EG calcium velocity, corresponding approximately to rest and sleep (defined as 5 min of immobility).

## EG homeostat does not follow sleep-related neurons

We next examined whether the observed dynamics in EG could result from activity of previously described homeostatic sleep-regulating neurons in the same brain compartments. In the EB, ring neurons (R5) increase calcium activity recorded in brain explants after prolonged sleep deprivation, consistent with a sleep homeostat^17,35^. A second component of this circuit, dFB neurons, innervate the FB and are frequently used as a ‘sleep switch’ to induce sleep^18,36^.

**Extended Data Fig. 7:**
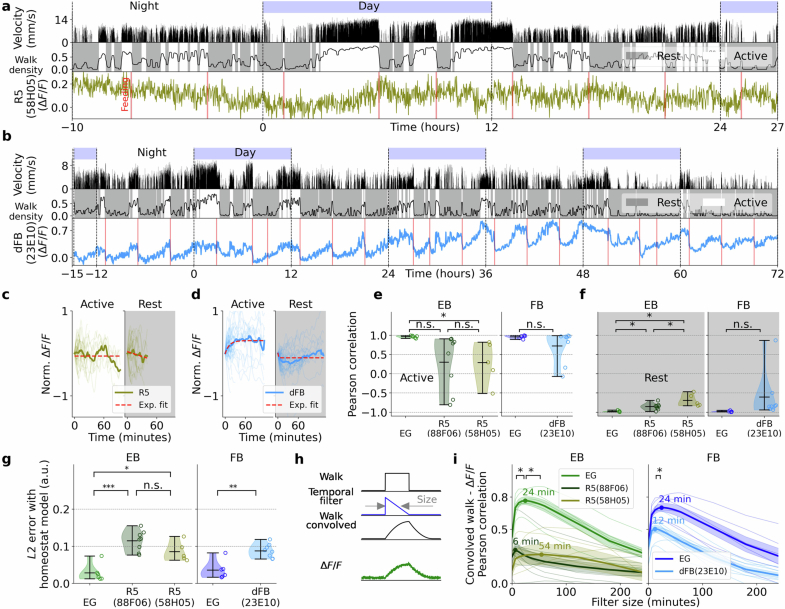
Calcium dynamics in sleep-related neurons (dFB, R5) and comparison with EG. Calcium dynamics in sleep-related neurons and comparison with EG. **a** Imaging R5 neurons (labeled by R58H05). Top row: day/night cycle. Second row: fly velocity. Third row: ‘walk’ density, rest (gray), active epochs (white). Fourth row: calcium activity in R5. ROIs in EB in Extended Data Fig. 1. **b** As a, but for dFB neurons (R23E10). **c** Normalized single (thin) and average (thick lines) fluorescence and exponential fits (red lines, see Methods), during active (*n* = 39 traces) and rest epochs (n = 29) for R5 in a. **d** As c, but for dFB in b (n= 66 traces during active, n= 57 during rest). ROIs in FB in Extended Data Fig. 1. **e** Left side: Pearson correlation between time flies are active and average fluorescence traces for EG (in EB), R5 (R88F06 and R58H05). Right side: as left side, but for EG (in FB) and dFB neurons. Number of data points in panels **e**, **f**, **g** and of traces in panel i are: n=5 flies for EG (in EB and FB), n= 8 for R5 (R88F06), n=5 for R5 (R88F06), n= 8 for dFB. **f** Pearson correlation between time resting and average fluorescence from EG and neurons, as in e. **g** L2 error between fitted homeostat model and activity of EG and neurons in EB (left) and FB (right). In e-g, circles: individual data points; short horizontal black lines: maximum, mean, and minimum in descending order. **h** Computing correlation between calcium activity and convolved ‘walk’: walk is obtained from behavior of fly (first row); ‘walk’ is convolved with triangular temporal filter with defined size (second row) to obtain ‘convolved walk’; correlation between calcium and ‘convolved walk’ is obtained. **i** Pearson correlation between ‘convolved walk’ and calcium for different filter sizes (x-axis) for EG, neurons in EB (left), FB (right). Thick lines, light regions, and thin lines: average, standard error of the mean, single correlation traces for each fly. In panels **e**, **f**, **g**, and **i**: one, two, or three asterisks indicate significance (two-sided t-test, *p* < 0.05, *p* < 0.005, and p < 0.0005, respectively). All p-values are corrected using the Benjamini-Hochberg procedure (Supplementary Data 1).

We recorded calcium activity of R5 neurons in the EB (with GAL4 lines R58H05 and R88F06; Extended Data Fig. 1d,e), and in dFB neurons (23E10-GAL4; Extended Data Fig. 1f), using multiple calcium indicators (GCaMP7f, GCaMP8f and jGCaMP8m), as well as imaging protocols (Extended Data Fig. 7a,b, Supplementary Figs. 30–40 and Methods). To assess whether these neurons encode homeostats similarly to EG or AL in the respective neuropils, we first again determined active and rest epochs based on behavior (Extended Data Fig. 7c,d and Supplementary Figs. 31, 35 and 40). We then computed the correlation between active (Extended Data Fig. 7e) and rest epochs (Extended Data Fig. 7f) and the normalized calcium activity of neurons and EG (to avoid saturation, we limited this analysis to traces of 30 min). For a sleep homeostat, the correlation should be positive during active epochs (increasing activity over time) and negative during rest or sleep (decreasing activity over time). This was, on average, the case for both neurons and EG, but the correlations were considerably higher for EG than for R5 neurons (Extended Data Fig. 7e,f). Calcium activity of dFB neurons displayed high correlations during active epochs, whereas the slope of decreasing activity in dFB neurons was close to zero during rest or sleep (Supplementary Fig. 41 and Methods). Immobility-based and activity-based analyses, such as a two-state model (Supplementary Figs. 32, 36, 37, 42 and 43 and Extended Data Fig. 7g), or an analysis using temporal filtering (Extended Data Fig. 7h,i) could not explain the neural data either (Supplementary Results 1.2 and Methods). Together, this suggests that activity observed in EG does not simply reflect activity of homeostatic neuronal circuits in the underlying compartments. Additionally, EG better represent sleep homeostasis for the naturally occurring sleep and wake bouts than either R5 or dFB neuronal activity recorded in the EB and FB, respectively.

## dFB neurons encode hunger and walking

**Extended Data Fig. 8:**
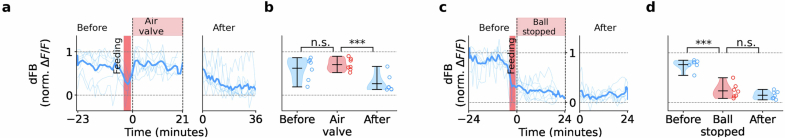
Change in dFB calcium activity after feeding depends on behavioral state. State-dependent reset of dFB neurons after feeding **a** Calcium in dFB during sleep deprivation, perturbing ball-supporting air stream after feeding. Normalized fluorescence traces of dFB activity (thin lines, n= 7 traces, n= 5 flies) and average (thick) before, after feeding while air valve was perturbing ball, and after termination of perturbation. **b** Normalized fluorescence levels from a (n= 7 traces, n= 5 flies, y-axis shared with a) before, after feeding, during sleep deprivation, after offset of deprivation. Sleep deprivation performed as above (interrupting air stream to ball inducing walking). **c**, **d** Same as a and b, but with sleep deprivation by blocking the ball to induce effortful behaviors, not walking (n= 8 traces for n= 6 flies). In b, d circles: individual data points; short horizontal black lines: maximum, mean, and minimum in descending order. Three asterisks indicate significance (two-sided t-test, p < 0.0005, Supplementary Data 1).

Activity in dFB neurons was correlated with walking speed, and rapidly reset during feeding (in several cases limited by the time resolution of recordings of 1 min). The rapid reset of activity after feeding in dFB neurons is not consistent with the behavior of a sleep homeostat, and it depended on the behavioral state of the fly. Specifically, if the fly was sleep deprived immediately after feeding using a method that induced bouts of fast walking, fluorescence activity increased again (Extended Data Fig. 8a,b and Supplementary Fig. 44), but if sleep deprivation was induced using a method that did not trigger coordinated walking (Methods), fluorescence reset (Extended Data Fig. 8c,d and Supplementary Fig. 45); this indicates that dFB neurons track feeding and walking state, but not behavioral effort or wakefulness. By contrast, EG activity remained high during both types of post-feeding sleep deprivation (Figs. 3e,f and 4a and Supplementary Figs. 20a and 18), as would be expected of a rest or sleep homeostat.

**Fig. 5:**
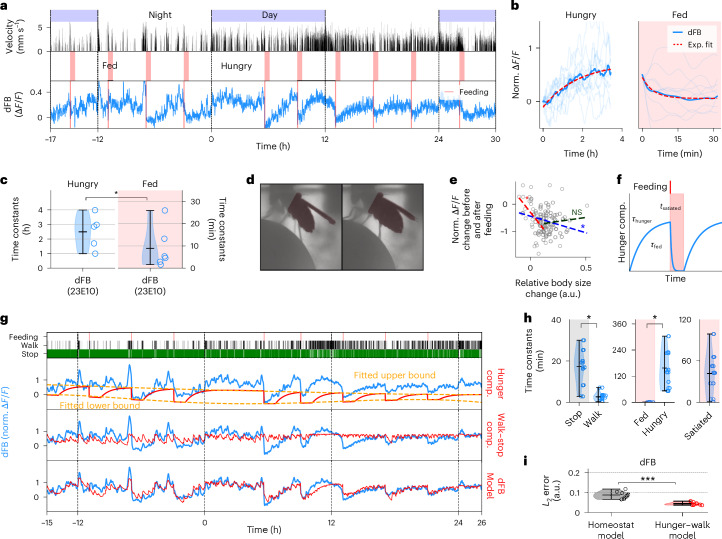
Feeding-related modulation in dFB neurons. **a**, Long-term imaging in dFB neurons. Top row: day and night cycle in VR. Second row: fly velocity. Third row: ‘hungry’ epochs before feeding (white region) and ‘fed’ epochs after feeding (red region). Fourth row: calcium activity in dFB neurons. **b**, Normalized individual (thin) and average (thick lines) fluorescence traces and exponential fits (red lines; Methods) during hungry (*n* = 11 traces) and fed epochs (*n* = 10 traces) of recordings in dFB neurons in **a**. **c**, Time constants of fitted exponentials during hungry and fed epochs (*n* = 8 flies). **d**, View of fly during imaging before (hungry, left side) and after (fed, right side) feeding. Mask of abdomen (highlighted in dark color) for computing change in size. **e**, Normalized fluorescence change against relative change in body size (Methods) between 10 min before and 10 min after feeding. Blue line represents a linear fit over all points (*n* = 107 feeding events from *n* = 8 flies); red and green lines represent a linear fit over the first and second half of relative body size changes (Pearson correlation, *P < 0.05; for detailed statistical information, see Supplementary Data 1). **f**, Parametrization (*τ*_hunger_, *τ*_fed_ and *t*_satiated_) of the hunger component of the hunger–walk model. Before feeding event (top row, red), hunger component increases with time constant *τ*_hunger_ and resets after feeding with time constant *τ*_fed_ for a given time *t*_satiated_. **g**, Fitting of the hunger–walk model to activity of dFB neurons. Top row shows events of feeding, walking and stopping. Second, third and fourth rows show normalized fluorescence (blue) and the fitted hunger component, walk–stop component and hunger–walk model (combination of the two previous components, red). **h**, Time constants (*n* = 8 flies) of stop and walk (left, from stop-walk component), and time constants of ‘fed’, ‘hungry’ and ‘satiated’ (center and right, from hunger component) with violin plots (see **i**). **i**, *L*_2_ error between fitted homeostat and hunger–walk models and normalized activity of dFB neurons with violin plots (*n* = 8 flies). In **c**, **h** and **i**, statistical significance was assessed using a two-sided *t*-test (*P < 0.05, ***P < 0.0005; for detailed statistical information, see Supplementary Data 1); circles denote individual data points; short horizontal black lines denote maximum, mean and minimum values in descending order.

Activity of dFB neurons increased with time since the last feeding event. Therefore, we averaged fluorescence traces of dFB neurons during hungry and fed epochs, defined as periods of 3.5 h before and 0.5 h after feeding events (separated by 4 h), respectively (Fig. 5a, see below and Methods for a more detailed model). Activity of dFB neurons exponentially approached an upper limit during hungry epochs, with time constants in the order of hours (Fig. 5b,c, and Supplementary Fig. 46). The calcium decay in dFB neurons during fed epochs was correlated with the amount of food ingested. The correlation between the relative change in abdomen size before feeding and after feeding (as a proxy for the amount of food ingested; Methods) and the change in dFB neuron fluorescence are shown in Fig. 5d,e. This correlation was stronger for small changes in abdomen size (Fig. 5e). These results suggest that dFB neurons can encode hunger, as has also been suggested for other FB neurons^37,38^.

Therefore, we fitted the fluorescence activity of dFB neurons with a homeostatic hunger model, which exponentially resets at feeding events and exponentially approaches an upper limit after an epoch of satiety (Fig. 5f) and a walking component (Figs. 4g and 5g–i and Supplementary Figs. 47 and 48; see Methods and Supplementary Results 2.2 for the ‘hunger–walk’ model). These findings show that activity in dFB neurons is modulated by hunger, consistent with a role of FB in feeding behavior^37,38^ rather than sleep^21,39^.

## Glia detect changes in CO_2_ concentration

**Fig. 6:**
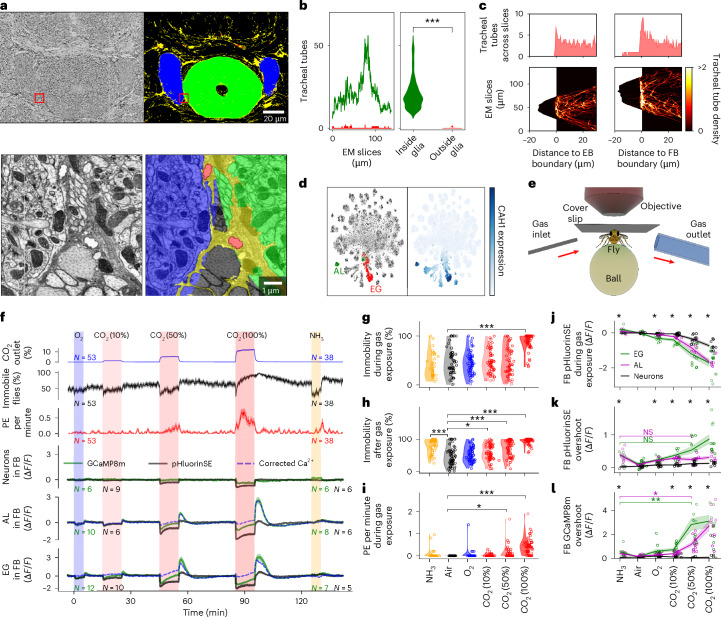
Trachea, CO_2_ and pH. **a**, Left: slice from EM hemibrain (top) and zoomed-in image (red, bottom). Right: EB (green), FB (blue), glia (yellow) and trachea (red). Downloaded and adapted from ref. ^40^. **b**, Number of tracheal tubes per slice (left) and distribution across slices (right) detected inside (green) or outside (red) glia. Significance was assessed using a Kolmogorov–Smirnov test, ***P < 0.0005; for detailed statistical information, see Supplementary Data 1. **c**, Distribution of trachea across slices (top) or for each slice (bottom) relative to EB (left) and FB (right) boundary. Negative distance indicates trachea inside neuropils. **d**, Right: single-cell assay for transposase-accessible chromatin using sequencing of brain projected in two dimensions (*t*-distributed stochastic neighbor embedding; data from ref. ^45^). EG (red), AL (green). Left: CAH1 expression. **e**, Setup: inlet delivers, outlet removes gas. **f**, First row: time of gas delivery. Air was delivered between other gases at the same rate. *n* = 53 flies exposed to oxygen and CO_2_ (left), *n* = 38 flies also exposed to ammonia (right). Second row: CO_2_ levels at outlet. Third row: the percentage of immobile flies. Fourth row: average proboscis extensions per minute. Fifth, sixth and seventh rows: jGCaMP8m (green) and pHluorinSE (black) in neurons (N), EG and AL. Colored lines and regions denote the mean and s.e.m. for each group. Single traces for each group are shown in Supplementary Figs. 49–54. pH-corrected calcium signal (EG, AL) in blue (Extended Data Fig. 9). Total number of flies (*N*) exposed to oxygen and CO_2_ on the left (jGCaMP8m in green, pHluorinSE in black), including a subset of flies also exposed to ammonia (*N*, right). **g**,**h**, Immobility of flies (Methods) with violin plots during (**g**) and after (**h**) gas delivery (see **i**). **i**, Proboscis extensions per minute during gas delivery. In **g**–**i**, *n* = 53 flies for air, oxygen and CO_2_, *n* = 38 flies for ammonia (two-sided Kolmogorov–Smirnov test, *P < 0.05, **P < 0.005 or ***P < 0.0005; for detailed statistical information, see Supplementary Data 1). Maximum, mean and minimum values in violin plots are not shown for clarity. **j**, pHluorinSE in the FB during gas delivery for N (black), EG (green) and AL (purple; see **l**). **k**,**l**, Overshoot of pHluorinSE (**k**) and GCaMP8m (**l**) in the FB after gas delivery. In **g**–**l**, colored circles, lines and regions denote individual data points, mean and s.e.m. for each group. **f** shows number of points (*N*) per group: air, oxygen, CO_2_ and ammonia. Black asterisks at the top of the graphs in **j**–**l** indicate significance between N, EG and AL for each gas (one-way analysis of variance). In **k** and **l**, two colored asterisks indicate statistical significance between ammonia and CO_2_ (50%) for EG (green) and AL (purple; two-sided *t*-test, *P* < 0.005; for detailed statistical information, see Supplementary Data 1). See Extended Data Fig. 10 for analysis in the EB.

What mechanism underlies the integration of behavioral activity over time in AL and EG? The structure of EG, forming diffusion barriers around brain areas, could enable them to detect the accumulation of metabolites inside corresponding compartments. We noticed in the fly connectome^40^ that glia surround tracheal tubes, which are responsible for gas exchange, as similarly observed in *Drosophila* larvae^41^. By segmenting the tracheal system, glial matter, and EB and FB compartments (Fig. 6a), we found that tracheal tubes exclusively occupy the space between brain compartments that is also covered by glia (Fig. 6b). Furthermore, tracheal tubes remain at the boundaries and do not enter the neuropil (Fig. 6c). This suggests a close interaction between the respiratory system and EG (Methods).

Therefore, we hypothesized that the observed calcium dynamics could be linked to metabolic gases such as CO_2_. Since the metabolic demand of neurons is higher during behavioral activity than during rest^42^, more CO_2_ is produced during wakefulness than during sleep in flies^43^. In mammals, glia support the metabolic activity of neurons in part by processing gases such as CO_2_ (ref. ^44^). In a single-cell transcriptome dataset^45^, we further observed that carbonic anhydrase (CAH1), an enzyme that catalyzes the hydration of CO_2_, is highly expressed in AL and EG (Fig. 6d), additionally suggesting that these glia are important for CO_2_ processing in flies.

We next tested whether changes in CO_2_ concentration in the brain induce changes in EG and AL calcium levels. We exposed flies in the imaging setup to a CO_2_ plume (Fig. 6e). At the two lower concentrations tested, CO_2_ induced walking behavior as shown before^46^, followed by rest or sleep after CO_2_ offset (Fig. 6f–h), whereas the highest concentration induced immobility (Fig. 6f–h). We imaged calcium activity in EG (56F03-GAL4) and AL (86E01-GAL4) as above, as well as in neurons by expressing sensors pan-neuronally using 57C10-GAL4 (Methods). To measure changes in pH, we expressed the sensor pHluorinSE in these lines (Methods).

**Extended Data Fig. 9:**
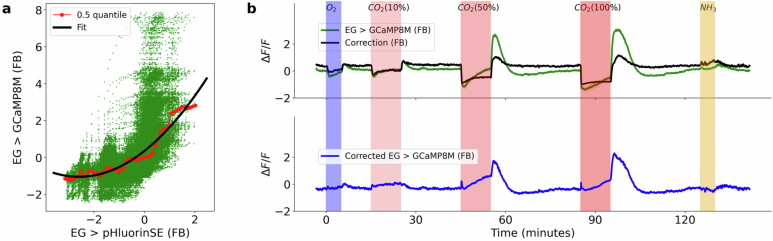
Correction of calcium sensor response for pH during response to CO_2_ in EG. jGCaMP8m correction for pH during response to CO_2_ in EG. **a** pHluorinSE versus jGCaMP8m expressed in EG in FB (orange, n= 208800 points) during the gas exposure experiment (Fig. 6f). Red points indicate 0.2 quantiles of jGCaMP8m values for each pHluorinSE bin (see Methods), while black line is a polynomial fit of the red points. **b** First row: average jGCaMP8m in FB of EG (orange) and jGCaMP8m correction (black) obtained from a. Second row: corrected jGCaMP8m signal (green), obtained from difference between recorded jGCaMP8m (green, first row) and correction (black, first row).

Exposure to CO_2_ (Fig. 6f) led to an initial, rapid decrease in fluorescence of both calcium and pH sensors in neurons, AL and EG (Fig. 6j; due to a change in pH^1,47^ resulting from diffusion of CO_2_ into tissue^48^), followed by a slow increase in calcium levels in EG and AL only; CO_2_ offset induced an overshoot for both calcium and pH sensor signals (Fig. 6f). After correction for pH sensitivity of the calcium sensor (Extended Data Fig. 9 and Supplementary Results 1.5), a net slow increase in calcium during CO_2_ exposure results, followed by an overshoot. This suggests a slow homeostatic calcium response counteracting pH changes in AL and EG, but not in neurons. Oxygen (O_2_; Fig. 6f) led to only a small decrease in calcium levels in EG. Ammonia (NH_3_) led to a long-lasting increase in pH sensor signal expressed in EG and AL (Fig. 6j,k), consistent with alkalinization. NH_3_ or 50% CO_2_ exposure induced similar changes in pH after exposure (Fig. 6k). However, calcium overshoot occurred only after CO_2_ exposure (Fig. 6l), indicating that fluorescence changes in jGCaMP8m were not exclusively due to the impact of pH alkalization on the calcium sensor (see Supplementary Results 1.5 and Supplementary Figs. 49–54 for single traces for each group).

Immobility after the termination of CO_2_ exposure lasted beyond the time required for EG and AL calcium to reset to baseline, indicating longer-lasting behavioral effects of CO_2_. High CO_2_ concentrations also induced proboscis extension (Fig. 6i, see also end of next section). In conclusion, glia but not neurons respond to changes in CO_2_ concentration with slow compensatory or homeostatic calcium transients.

## Optogenetic activation of AL and EG induces immobility and proboscis extension

**Fig. 7:**
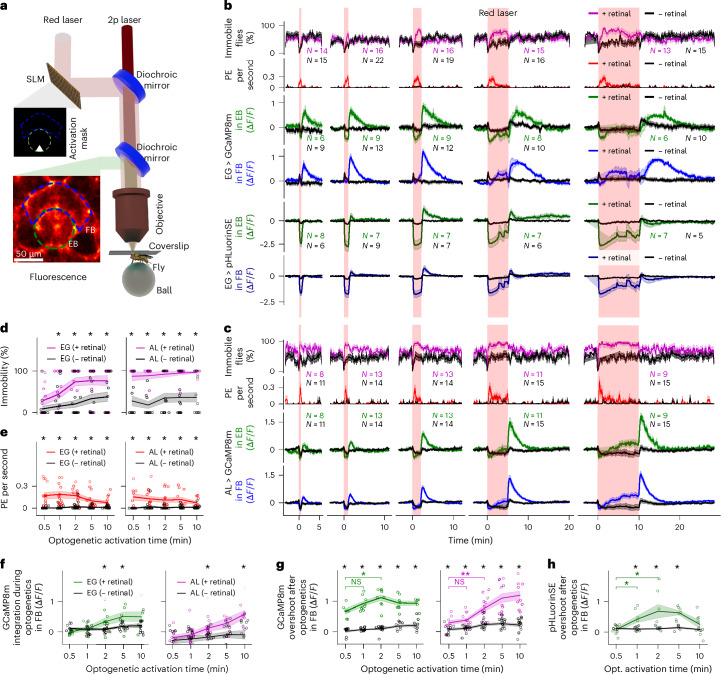
Combined optogenetics and imaging in EG and AL during behavior. **a**, Setup: activation (white triangle indicates a section of EB) generated by red laser and SLM (Methods). **b**, Optogenetics in EG: columns show trials with different activation duration: 0.5, 1, 2, 5 and 10 min (red vertical areas). First row: the percentage of immobile flies. Second row: average proboscis extensions per second. Number of trials for first and second rows (*N*) for retinal-fed flies (retinal flies, black, 12 flies), controls (flies fed with standard food, black, 15 flies), combining flies expressing GCaMP8m or pHluorinSE. Third and fourth rows: GCaMP8m in retinal flies (green, EB; blue, FB) or controls (black). Trial numbers for *n* = 6 retinal flies (green), *n* = 9 controls (black). Fifth and sixth rows: pHLourinSE for retinal flies (dark green, EB; dark blue, FB) or controls (black). Trial numbers for *n* = 6 retinal flies (green), *n* = 6 controls (black; see **h**). **c**, As in **b** for AL. Traces in **b** and **c** in Supplementary Figs. 56 and 57. **d**, Immobility (see **h** and Methods) during optogenetic activation for each trial (*x* axis) in EG (left) and AL (right). Purple, retinal flies (*N* of trials in **b** and **c** in purple). Black, controls (*N* of trials in **b** and **c** in black; see **h**). **e**, Proboscis extensions per second (see **h**) during optogenetics for each trial (*x* axis) in EG (left) and AL (right). Red, retinal flies (*N* of trials shown in **b** and **c** in purple). Black, controls (*N* of trials in **b** and **c** in black) (see **h**). **f**, GCaMP8m in the FB, 0.9 quantile during optogenetic activation for each trial (*x* axis) in EG (left) and AL (right; see **h**). **g**, GCaMP8m overshoot in the FB, defined as 0.9 quantile after optogenetic activation for each trial (*x* axis) in EG (left) and AL (right; see **h**). **h**, pHLourinSE overshoot in the FB, as in **g**. In **f**–**h**, black represents control flies. *N* of trials for retinal-fed flies in **b** (EG) and **c** (AL) in green and for controls in black. In **d**–**h**, colored lines, regions and circles denote the mean, s.e.m. and individual points for each group, respectively. Black asterisks denote significance between + and − retinal-fed flies (two-sided *t*-test, *P* < 0.05). Colored asterisks denote significance across + retinal-fed flies for different optogenetic activation times (green, EG; purple, AL) using a two-sided *t*-test (*P < 0.05, **P < 0.005; for detailed statistical information, see Supplementary Data 1). See Supplementary Fig. 58 for EB.

The dependence between calcium activity and behavioral state (Figs. 1 and 2 and Extended Data Fig. 6) raises the question of whether glia only track behaviors, or also control them. We, therefore, expressed the photoactivatable channel CSChrimson (Methods; more than 90% of CSChrimson current is due to protons and not due to calcium^49^), together with jGCaMP8m in EG or AL. A spatial light modulator (SLM) was used to activate CSChrimson in defined patterns using red light (Fig. 7a, Methods and Supplementary Fig. 55) while at the same time imaging calcium activity in flies walking on an air-supported ball as before.

Optogenetic activation of either EG or AL in a section of EB (Fig. 7a and Methods) induced immobility (see single traces in Supplementary Figs. 56 (EG) and 57 (AL)), as indicated by the percentage of immobile flies following activation (Fig. 7b–d). Thus, even local activation of AL and EG can cause immobility, similarly to thermogenetic manipulation of all EG^25^ or AL^26^.

Optogenetic activation of both EG and AL also induced proboscis extension (Fig. 7b,c,e), although at higher frequency than during spontaneous sleep (Supplementary Fig. 58a)^13,21,22^. Proboscis extension is most frequent early in spontaneous sleep bouts^22^, when acidification of neurons is likely higher due to continued prior neural activity^1,50–52^, and could serve to remove CO_2_ (that is, reduce acidification) from the brain^53^. Our finding of stronger proboscis extension due to optogenetic activation of either EG or AL at the beginning of the stimulation when the drop in pH is largest (Fig. 7b) would also agree with such a mechanism (see Fig. 2i for spontaneous rest and sleep).

Fluorescence signals in the EB and FB of EG and AL rapidly decreased at the onset of activation, consistent with acidification of the activated compartment (EB)^49^, as similarly observed during CO_2_ exposure (Fig. 6). We confirmed that optogenetic activation of EG leads to a decrease in pH in EG as measured with pHluorinSE (Fig. 7b). Similar to experiments with CO_2_ exposure (Fig. 6), the initial decrease in fluorescence was followed by a slow drift of calcium levels during activation in both EG and AL (Fig. 7f), and termination of activation again led to an overshoot response in both EG and AL (Fig. 7g; here we did not compute a correction for pH sensitivity of the sensor, due to the more limited pH range compared to CO_2_ experiments, where ammonia stimulation was used to broaden the pH range; Methods). As a control for the specificity of optogenetic activation, flies were not fed retinal food, which prevents the light-induced activation of Chrimson; immobility was significantly more prevalent in retinal-fed flies than in control flies (Fig. 7d). Activation of EB in EG or AL in control flies nevertheless led to small changes in calcium activity in both EG and AL, mostly during but not following optical stimulation. These small calcium changes could be due to residual or modified activity of CSChrimson in the absence of retinal. Local optogenetic activation of the EB in both EG and AL also induced calcium signals in the FB, suggesting that EG and AL interact across neuropils.

**Extended Data Fig. 10:**
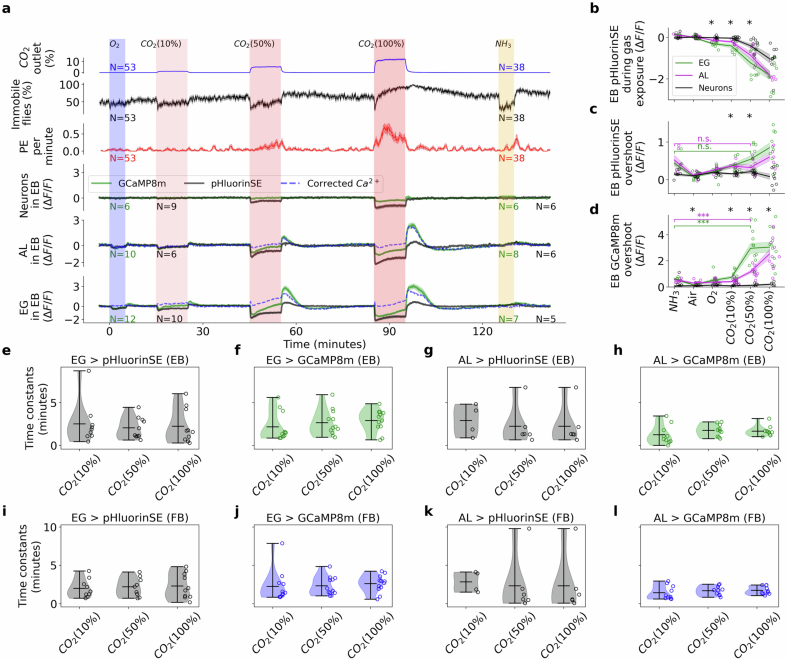
Analysis of calcium and pH sensor responses to CO_2_ exposure in glia. **a** Same as Fig. 6f but for EB. The total number of flies N considered for each row is shown on the left with the color representing each group. Only a subset of these flies were exposed to ammonia, shown on the right. **b-d** Same as Fig. 6j-l but for EB (see statistical data in Supplementary Data 1). **e-h** Fitted time constants for the decay after CO_2_ exposure in EB for EG and AL expressing pHluorinSE and jGCaMP8m, respectively. **i-l** Same as e-h but in FB. Same as e-h but in FB. In **b**-**l**, circles: individual data points; short horizontal black lines: maximum, mean, and minimum in descending order. The number of flies considered in panels **b**-**l** for each group correspond to the number of flies shown in panel **a**.

Glial activity reset more slowly during spontaneous sleep than following manipulations with CO_2_ or optogenetics (see time constants in Fig. 1l, Extended Data Fig. 10e–l and Supplementary Fig 58d,e). This discrepancy may result from unphysiological conditions that drive fast and strong pH shifts from acidic to alkaline states, disrupting natural glial dynamics.

## Controller model of calcium dynamics

**Fig. 8:**
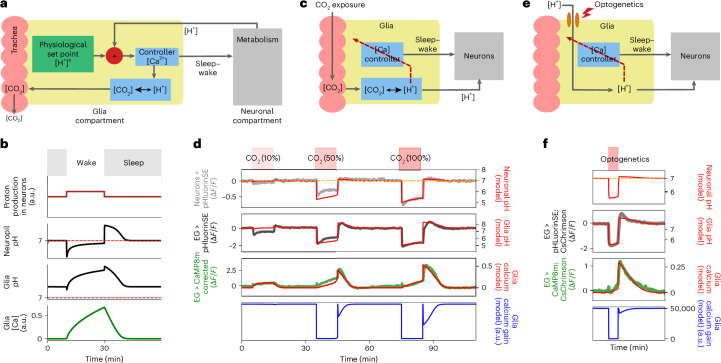
Controller model for glial calcium dynamics. **a**, Schematic of glia acting as a pH controller: EG (yellow) comparison of optimal physiological set point with pH changes (protons) in neuropils (gray). Negative deviations of pH from set point increases calcium, which is assumed to shift equilibrium between CO_2_ and protons to the left, increasing CO_2_ concentration for diffusive removal. **b**, Model simulation during wake and rest or sleep bouts. During wake (white area), more protons are produced in neuropils (first row) compared to rest or sleep (gray area). This leads to a decrease in pH in neurons (second row) and a pH increase in glia (third row). Calcium in glia increases (fourth row) to maintain pH in the neuronal compartment at set point (pH = 7). **c**, Controller schematic during CO_2_ experiments. High concentration of CO_2_ enters tracheal tubes, shifting equilibrium between CO_2_ and protons to the right, lowering pH in glia and in neurons through diffusion. At very low pH, the calcium controller is partially inhibited (red dashed arrow). **d**, Model dynamics during CO_2_ exposure. First row: application of 10%, 50% and 100% CO_2_. Second and third rows: average of fluorescence traces in neurons (measured with pHluorinSE, gray) and EG (black), as in Fig. 6f. Simulated dynamics of pH inside neuronal and glial compartments are shown in red. Fourth row: pH-corrected jGCaMP8m in EG (Methods and Supplementary Results 1.5), together with the simulated calcium controller (red). Fifth row: simulated gain of the calcium controller, partially inhibited during CO_2_ application, produces slow dynamics and calcium overshoot afterwards (Supplementary Fig 59 and Methods). Partial inhibition of the controller leads to a weak response, leading to slow integration. **e**, Controller schematic during optogenetics. During optical stimulation, protons enter glial cells and lower pH. At very low pH the calcium controller is partially inhibited (red dashed arrow). **f**, Model dynamics during optogenetic experiments. Second and third rows: simulated pH dynamics in neurons and glia. Fourth row: average of calcium traces in EG during optogenetics in the FB (taken from Fig. 7b, third column) compared to simulated calcium dynamics (red). Fifth row: simulated gain of the calcium controller partially inhibited during optogenetic activation.

Both CO_2_ and optogenetic experiments revealed similar calcium and pH dynamics in AL and EG (Figs. 6 and 7). CO_2_ in solution is in equilibrium with bicarbonate and protons, and this equilibrium can be modulated in (mammalian) glia by carbonic anhydrases, pH buffers and ion channels^47,51,54^. To describe the observed dynamics, we use a model with two compartments, a neuronal compartment and a glial compartment (Fig. 8a and Methods). We assume that pH in neurons is detected by glia and that a calcium-dependent mechanism maintains pH at a physiological set point^55^. Deviations from the set point led to changes in glial calcium levels (Supplementary Results 2.3–2.8).

Model simulations (Fig. 8b) show that neural metabolic activity during the wake state lowers pH in the neuronal compartment. This drop in pH is detected by glia, triggering an increase in calcium in glia, which restores pH in neurons to the set point, by shifting the equilibrium from protons to CO_2_ in glia for removal through respiration (Fig. 8a). This produces an increase in pH in the glial compartment. Glial alkalinization in the model accelerates proton diffusion from the neuronal to the glial compartments, bringing the neuronal pH back to its set point. When calcium activity reaches an upper threshold, it triggers rest or sleep, which inhibits neural activity and prevents further acidification and allows resetting.

Drift and overshoot dynamics resulting from CO_2_ exposure or optogenetics can be modeled (Fig. 8d,f) by assuming that calcium regulation is partially inhibited when pH is outside normal physiological values (Fig. 8c,e and Supplementary Fig. 59), as is likely the case for the strong perturbations induced during CO_2_ exposure or optogenetic activation. When pH rapidly rises after CO_2_ exposure or optogenetic stimulation, the inhibition of the calcium controller is lifted (Fig. 8c,e). The controller (in the glia) now detects a sharp pH deviation from the set point. This results in a strong calcium and pH overshoot in glia (Methods; see Supplementary Results 1.5 for correction of jGCaMP for pH, and Supplementary Results 2.8 for possible biological mechanisms underlying model assumptions).

Any processes that result in higher metabolic activity, for example, active behavior compared with rest or sleep, lead to CO_2_ production and ATP hydrolysis and corresponding acidification^1,51^. The strong and fast perturbations resulting from high concentrations of CO_2_ or optogenetics are likely unphysiological. However, neural activity might lead to weaker acidification, and we observed calcium dynamics at low concentrations of CO_2_ (10% in Fig. 8d), similar to the dynamics detected during wake and sleep behavior. Thus, the artificially induced responses to fast onset and offset of CO_2_ or optogenetic perturbations could be similar to the response of a controller measured with step function inputs.

## Discussion

Homeostatic control—sensing deviations from and resetting to a preferred physiological state—is essential for brain function, as exemplified by the restorative and beneficial functions of sleep across species. Here, by monitoring calcium dynamics in glia in flies navigating in VR, we link rest and sleep homeostasis, which divides fly behavior into many short epochs of activity and inactivity, to metabolism as reflected in pH and CO_2_ sensitivity. EG and AL show homeostatic calcium transients for the short rest and sleep bouts typical for the fly. Both also show calcium fluctuations on circadian timescales (Figs. 1 and 2). Local optogenetic activation of glia is sufficient to induce rest.

The finding that calcium activity of glia increases during behavior, corresponding to increased metabolic activity detected through CO_2_ or pH, is consistent with the idea that wakefulness is energetically challenging for the brain^4,56^. Time from onset of immobility to onset of deeper sleep needs to be measured in the brain, which could also be accomplished by measuring glial calcium velocity (Extended Data Fig. 6). In mammals, the transition from rest to sleep shows gradual as well as abrupt changes^24,57^. Gradual changes as described here for glial calcium levels could, therefore, also be part of sleep initiation in the fly.

Immobility continued after termination of AL optogenetic activation, but not after termination of EG activation, suggesting that local optogenetic activation of only one cell type does not replicate all aspects of elevated calcium activity resulting from behavioral activity. Additionally, the pH overshoot in EG observed after their optogenetic activation (Fig. 7b) causes glia to transition abruptly from an acidic to an alkaline state (as seen by the overshoot of the pH response; Fig. 7). This shift might be inconsistent with sleep and activate other processes that promote walking activity.

In the proposed model, rest or sleep is triggered when calcium reaches an upper threshold; this could for example prevent excessive energy expenditure on the glia controller^54^, allowing replenishment of energy storages, or restoration of pH bicarbonate buffers and substrates that are coupled with proton shuttles, such as lactate or glutamine. Dysregulation of astrocytic pH is linked to various brain diseases^54^. In our experiments, reaching the upper or lower thresholds of the EG or AL calcium homeostat did not immediately trigger sleep or awakening, potentially due to the uncertainty with which the threshold could be defined given the long-term calcium fluctuations. However, long bouts of activity with a saturated homeostat were only observed under mechanically or hunger-induced sleep deprivation (Fig. 4b,d), indicating that nevertheless calcium levels could control behavioral activity with a threshold-dependent mechanism, possibly taking into account circadian rhythms.

The finding that glia show not only slow integration of behavioral activity (Figs. 1, 2 and 3a–d), but also calcium peaks at activity onset after extended bouts of immobility (Fig. 3g), suggests that a more accurate controller model should also include a differential component. Such a differential control element, which anticipates future changes, would be consistent with the anticipatory metabolic activity observed in neurons^42^.

Different homeostatic drives, such as for feeding and rest or sleep, or in different brain compartments, can be combined in frameworks such as homeostatic reinforcement learning^58^. Our finding that glia around different brain compartments show different calcium dynamics could be due to locally different activity and corresponding sleep need, consistent with the idea of local sleep^19,20,26,59^.

The mechanism by which glia act on neurons likely involves the calcium-dependent release of gliotransmitters^16^. The modulation of neural circuits across brain areas then controls rest and sleep behavior. In mice, microglia, which share some of the functionalities of EG in flies, generate inhibitory feedback using adenosine^60^; EG in flies similarly release adenosine with increasing calcium levels^61^. The action of EG could also include the regulation of glutamate in the enclosed circuits^7,33^. Further, AL release d-serine^62^, which is also important for sleep in flies^63^.

Together, our data suggest that EG and AL are homeostatic controllers of the short activity epochs typical of the fly; although they sense local rest and sleep need, as they are distributed across the entire brain, they control behavior globally, likely by regulating neural circuits across different brain compartments. In the future, it will be of great interest to use different behavioral paradigms, including learning, to gain a better understanding of the different roles of rest and sleep for the brain.

## Methods

### *Drosophila* preparation

All flies were reared in an incubator at 22 °C with a 12-h light–dark cycle. For experiments with freely moving flies, we used 7-day-old females expressing jGCaMP8m^64^ in EG (UAS-jGCaMP8m;56F03-GAL4)^65^. Flies were anesthetized on ice and placed individually in rectangular chambers with food (Fig. 1a).

The same 12-h light–dark cycle as in the incubator was used in VR during imaging experiments. All imaging experiments were performed with female flies between 3 and 8 days old at the beginning of the experiment. To record calcium activity of EG, of AL and in neurons, we expressed jGCaMP8m using the GAL4 lines 56F03 (UAS-jGCaMP8m;56F03-GAL4), 86E01 (UAS-jGCaMP8m;86E01-GAL4) and pan-neuronally using 57C10-GAL4 (UAS-jGCaMP8m;57C10-GAL4)^65,66^, respectively. Experiments for monitoring extracellular glutamate were performed by expressing the glutamate sensor iGluSnFR in EG (UAS-iGluSnFR;56F03-GAL4). To monitor activity of R5 ring neurons, which also depends on the circadian rhythm^67^, we used two different GAL4 lines: 58H05-GAL4 expressing jGCaMP8m (UAS-jGCaMP8m;58H05-GAL4), and 88F06-GAL4 expressing jGCaMP8m, 8f or 7f (UAS-jGCaMP8m;88F06-GAL4, UAS-jGCaMP8f;88F06-GAL4 and UAS-jGCaMP7f;88F06-GAL4), respectively, and imaging protocols with different time resolutions to potentially detect faster oscillatory dynamics^68^. Activity of dFB neurons was monitored by expressing jGCaMP8m or jGCaMP8f in 23E10-GAL4 (UAS-jGCaMP8m;23E10-GAL4, UAS-jGCaMP8f;23E10-GAL4)^36^. To record pH in EG, AL and neurons during gas exposure experiments, we expressed pHluorinSE^69,70^ (Bloomington stock no. 82176) in the respective cell types (UAS-pHluorinSE;56F03-GAL4, UAS-pHluorinSE;86E01-GAL4 and UAS-pHluorinSE;57C10-GAL4). For optogenetic experiments, we expressed both jGCaMP8m and CSChrimson-mCherry^71^ (Bloominton stock no. 82180) in EG (UAS-CSChrimson, UAS-jGcAMP8m;56F03-GAL4) and AL (UAS-CSChrimson, UAS-jGcAMP8m;86E10-GAL4).

For imaging, flies were dissected using laser surgery as described^72,73^ to insert a transparent window into the cuticle^72,74^. The cut cuticle and air sacs were removed under a dissection microscope using forceps, either manually or with a microrobotic arm^72^. The opening was sealed with a drop of transparent UV glue (Detax 02829, freeform fixgel 3 g syringe, purchased before 2023 (ref. ^72^) or Norland Optical Adhesive NOA 68)^74^. Between two and five flies were dissected at a time and were left to recover in vials with food for 1 to 3 days before imaging. Flies were then glued to a glass slide using UV glue and transferred to the long-term imaging setup or to a second microscope modified for gas experiments. Flies were selected for imaging based on the optical access to the structures of interest, which varied depending on dissections. Only data recorded at least 48 h after surgery were included in the analysis.

For optogenetic experiments, flies expressing CSChrimson and jGcAMP8m were initially reared on regular food, with dissections carried out as previously described. After dissection, control flies were transferred to a vial containing regular food, while non-control flies were placed in a vial with retinal food. Both control and non-control flies were left to recover in darkness for 2 days before imaging experiments. Retinal food was prepared by adding 50 μl of a solution containing 100 mg of all-trans-retinal dissolved in 3.52 ml of 96% ethanol to vials containing regular food.

### Setup for experiments with freely walking flies

Flies were placed in transparent chambers of 70 × 4 mm (Fig. 1a) and activity was recorded in 15 chambers using a camera from above with a resolution of 1,920 × 1,080 pixels at 30 Hz. A rectangular grid of IR light-emitting diodes (LEDs) illuminated the chambers from below through a diffuser. A long-pass filter (Thorlabs, FGL780S) in front of the camera rejected white light from LEDs, which generated the same 12-h light–dark cycle as in the incubator. Because flies needed to adapt to the new environment, the first day of the experiment was discarded, and we only considered the subsequent 2 days for the following analysis.

Tracking of fly positions was done offline, using color segmentation in OpenCV and Python, by extracting the dark color of the flies from the bright background (Supplementary Video 8). The position and velocity of each fly were smoothed using a Kalman filter. Figure 1b shows examples of the position along the chamber and the velocity of one fly. Supplementary Figs. 1a,b show the mean velocity of 15 flies with a light–dark cycle and in darkness over 48 h, respectively. Under both conditions, flies displayed circadian activity.

To find epochs of immobility, we set the velocity of each fly to zero if a fly did not move for at least 0.25 body lengths in a second (a body length was defined as 2.5 mm; Fig. 1c). We then computed each fly’s ‘stop’ binary profile over time from the velocity (Fig. 1c). To compute sleep bouts, we used different temporal filters with a size of 1, 2, 5, 8 and 10 min (Fig. 1c) and convolved them with the binary ‘stop’ signal (Fig. 1c). These temporal filters removed small bouts of movement of the fly (depending on the filter size) between stop epochs. Sleep was then obtained by thresholding the convolved ‘walk’ signal at the value of 0.5 (Fig. 1c). The distribution of continuous sleep bouts was computed for each filter size during the day and night (Fig. 1d), and during darkness (Supplementary Fig. 1c), and the 90% quantile of the distribution is highlighted. A filter size of 2 min, which already removes up to 1 min of walking in between stop epochs, produces a distribution where 90% of sleep bouts are below 50 min (Fig. 1d). Sleep bouts were even shorter in constant darkness, where even using a filter size of 10 min, which removes 5 min of walking in between stop bouts, led to a distribution where 90% of sleep bouts were below 40 min (Supplementary Fig. 1c).

To compute how frequently flies ate in these behavior experiments, we determined all occasions where the fly was close to the food (at around 7 mm in the chamber; Fig. 1b). As before, we used temporal filters with different sizes (30, 60, 90, 120 and 150 s) to filter out small movements of the fly entering and leaving the food. We then obtained the distribution of time intervals between consecutive feeding events during the day and night depending on filter size (Supplementary Fig. 1d). Using a filter size of 120 s, 90% of the times between consecutive feeding events were around 26 min during both day and night. Similar rates of feeding were used in some of the imaging experiments, where flies were fed every 26 min (Fig. 1g and Supplementary Fig. 14) or every 16 min (Supplementary Fig. 15).

### Imaging setup

The two-photon^75^ imaging setup for long-term imaging was as described in refs. ^72,73^. Flies walked on an air-supported ball in a projector-based VR setup with a dark stripe in front of a blue background during the day (Fig. 1h), and in darkness during the night. Flies were fed in an automated way every 4 h with a needle approaching the fly to deliver food for 2 min (as shown in Supplementary Video 4 in ref. ^72^). For recording volumetric calcium activity and to reduce brain motion artifacts, two axially offset focal planes were recorded at the same time with beams with an extended focal length, with temporally multiplexed acquisition^72^. This setup allowed motion correction at high time resolution in all three dimensions as described in ref. ^73^. VR projection and closed-loop behavior were implemented as previously described^72,76^.

In addition, airflow to the ball could be interrupted using an automated valve for sleep deprivation at preset frequencies. Switching the airflow on and off typically induced fast walking activity. For blocking ball movement and to induce persistent behavioral activity (pushing and pulling on the ball, but not coordinated walking), a remote-controlled brush mounted on a motorized three-axis micromanipulator was used. Ball movement was blocked by positioning the tip of the brush on top of the ball in front of the fly and by pushing the ball against the ball holder. If the fly stopped moving, the brush was slightly repositioned, and due to the induced perturbation, the fly resumed active behavior.

### Data acquisition

At the start of each long-term imaging experiment, a *z*-stack of 100-μm depth with an axial step size of 0.25 μm was recorded, which was used for *z*-motion correction in post-processing^73^ (see next section). Additionally, an automated robotic feeder was configured at the beginning of the experiment, for example, to feed flies every 4 h for 2 min^72^. Imaging data were recorded at a resolution of 256 × 256 pixels at 60 Hz at the same time in two axially offset focal planes^72,73^. We typically recorded for 1 s with a wait time of 60 s between consecutive recordings, resulting in a total imaging time of 1.6% of the duration of the experiment, which helped to reduce photobleaching and phototoxicity. We additionally used a different imaging protocol in four other flies, where we recorded in trials of 30 s with a wait time of 5 min between trials^72^, which resulted in a total imaging time of 11% of the duration of the experiment (Supplementary Figs. 39g and 34f–h).

The VR, implemented as described previously^35,76^, displayed a dark stripe on a bright background using a blue laser (488 nm), which was switched off during the night for 12 h. The VR night and day cycle was the same as the one used in the incubator. Ball movement was tracked at 200 Hz and a calibration factor was determined for calculating ball velocities. The water temperature at the objective was maintained at 22 °C with a perfusion system^72^.

Flies were left to recover for at least 2 days after surgery and before imaging started. In some flies, imaging started after 1 day of recovery and the first day was excluded from data analysis. Recordings of calcium activity in EG are shown in Fig. 1i and Supplementary Figs. 3, 17, 14 and 15 for EB and FB, Supplementary Fig. 4 for LAL, and Supplementary Figs. 5 and 10 for MBs and ML. Recordings for AL are shown in Fig. 2a and Supplementary Fig. 12. Recordings of glutamate activity in EG are shown in Extended Data Fig. 4a and Supplementary Fig. 26. Recordings of calcium activity in R5 neurons using 58H05-GAL4 are shown in Extended Data Fig. 7a and Supplementary Fig. 30, and recordings using 88F06-GAL4 are shown in Supplementary Figs. 33 and 34. Finally, recordings of calcium activity in dFB neurons are shown in Extended Data Fig. 7b and Supplementary Figs. 38, 44 and 45.

For imaging experiments during gas exposure or optogenetics, flies were allowed to recover for 1–2 days after dissections. Imaging data were recorded at a resolution of 256 × 256 pixels at 60 Hz, simultaneously recording in two axially offset focal planes for 1 s every 5 s. *z*-motion correction was not applied in these experiments, since the brain was sufficiently stable throughout the 2-h experiment. Recordings with gas exposure are shown in Supplementary Figs. 49–54, while recordings with optogenetics are shown in Supplementary Figs. 56 and 57.

### Data post-processing

Imaging data were corrected for lateral motion by alignment with respect to a template (average of the first 60 frames) using cross-correlation (Fig. 1g). The *z*-stack was also aligned with respect to this template. Then, 24 ROIs were defined for recordings in EG in different brain areas (the 24 ROIs merged into a single one are shown in Fig. 1g for EB and FB, Extended Data Fig. 1b for LAL and Extended Data Fig. 1c for MBs and ML). ROIs were also defined for the case of R5 and dFB neurons (the combined 24 ROIs are shown in Extended Data Fig. 1d,e for R5 neurons, and Extended Data Fig. 1f for dFB neurons). The intensity of each ROI was computed by summing all pixel values within each ROI per frame as well as in each frame in the z-stack. The intensity of the ROIs was then used to estimate the *z*-motion of the sample for each imaging frame as described^73^. The resulting *z*-motion over the time of the experiment was filtered using a median filter with 1,000 points to discard high-frequency motion, and maximum displacements of around 10 μm along the *z* axis were detected. The filtered *z*-motion was used to estimate the fluorescence of each ROI corrected for *z*-motion^73^. Differences between corrected and uncorrected fluorescence were generally low, with maximum differences of 0.1 *ΔF*/*F* due to the extended focal volume used for imaging^73^.

The 200 Hz used to track ball motion underestimated ball displacements in the three axes by a factor of 0.50 compared to using 500-Hz tracking as in ref. ^76^. This factor was experimentally measured by rotating the ball by 3,600 degrees. Ball displacements during long-term imaging were corrected with this calibration factor in post-processing and used to compute the absolute velocity of the fly in bins of 1 s using the ball radius (3 mm).

For fitting models to fluorescence traces (see sections below), we first smoothed the fluorescence signal using a low-pass filter with a cutoff period of 30 min. Then, we normalized the filtered fluorescence, *F*(*t*), by remapping the 10% and 90% quantiles of *F*(*t*) (*Q*_10%_ and *Q*_90%_, respectively) to the values 0 and 1, respectively, with equation (1):

1 $$\,\text{Normalized}\,\,F(t)=\frac{F(t)-{Q}_{10 \% }}{{Q}_{90 \% }-{Q}_{10 \% }}.$$

This was done to reliably compare glia and neurons in Extended Data Fig. 7e–i, as different recordings have different fluorescence dynamic ranges and different noise levels. By smoothing and normalizing fluorescence traces from all experiments, we ensured that correlations (Extended Data Fig. 7e,f,i) and errors from model fittings (Extended Data Fig. 7g and Fig. 5i) were correctly compared across recordings.

### Behavior classification

Fly behavior was monitored during the entire experiment using a camera (Supplementary Fig. 16a) under IR illumination at ten frames per second. Based on blocks of ten consecutive frames (1-s time resolution), we classified the following seven behaviors (Supplementary Fig. 16a). (1) Stop: the fly does not move. (2) Proboscis: the fly does not move but extends and retracts the proboscis. (3) Walk: the fly walks on the ball. (4) Discomfort: the fly pushes or pulls the ball. (5) Grooming front: grooming of head and proboscis with front legs. (6) Grooming back: grooming of the abdomen and wings with hind legs. (7) Feeding: fly is fed with the feeding robot.

We used a three-dimensional (3D) convolutional neural network (3D CNN) to classify behavior based on ten frames at a time. The field of view (FOV) of the camera (with resolution 640 × 480 pixels) was cropped around the fly and was resized to a resolution of 256 × 256 pixels. The architecture of the 3D CNN is shown in Supplementary Fig. 16b, where each convolutional block consisted of a 3D convolutional layer with a 3 × 3 × 3 kernel size and rectified linear unit activation function, a dropout layer with a rate of 0.2 to prevent overfitting and 3D maximum pooling with size 2 × 2 × 1. The number of filters of the convolutional layer increased by a factor of 2 in each consecutive convolutional block. Finally, a dense layer with a SoftMax activation function assigned a value to each class. The 3D CNN had a total of around 6.5 million trainable parameters.

A total of 45,610 manually labeled frames from ten different flies were used for training. The dataset was normalized to train the network with the same number of samples for each class. We further increased this dataset with data augmentation that randomly transformed the input frames by changing illumination, translation, rotation and/or scale during training. To train the network, we used TensorFlow with a binary cross-entropy loss function and Adam as the optimizer. After 150 epochs with a batch size of 50 samples, the loss function reached a minimum saturation value in about 2 h, using four QUADRO GPUs.

To test the performance of the network, we used 2,410 labeled frames, which were augmented to a total of 20,000 using augmentation transformations. The accuracy of the network to predict the right class of behavior was 98.6%, and the normalized confusion matrix for each class is shown in Supplementary Fig. 16c. An example of behavior classification is shown in Supplementary Video 7.

### Probing of arousal threshold

For probing the arousal threshold of the fly during long-term imaging, we used a total of six flies where we recorded calcium activity from EG using the same protocol as before. We recorded multiple trials of probing the arousal threshold in each fly. Trials were initiated automatically using custom software (see number of trials, *N*, for each fly in Extended Data Fig. 3b–g). At the beginning of each trial, the amount of expected sleep time for probing the arousal threshold was selected randomly, either 30 s or 5 min. Then, the fly was stimulated to walk, by closing and opening the valve controlling the airstream supporting the treadmill ball repeatedly three times during 6 s (1 s closed and 1 s open). This ensured that the fly was awake at the beginning of each trial. After this, the velocity of the fly on the ball (thresholded to remove tracking noise) was monitored in real time to detect epochs of walking or immobility. A timer was used to measure the time the fly was not walking since the end of the last walking bout (nonzero velocity). If this time reached the previously selected duration (30 s or 5 min), probing of the arousal threshold started. Probing was performed with an IR laser focused on the abdomen (Toptica, ibeam-smart-785-S-HP with pulse option, 785 nm) by ramping the power from 0 to 100 mW in steps of 1 mW every second. When the fly started to walk due to the heating effect of the IR laser, the trial finished and the IR laser was turned off. During probing with the IR laser, the shutter of the two-photon laser was closed and imaging data were not recorded, because the combined power of the two-photon and heating lasers would distort the detection of the threshold. After a trial had finished, the next trial started after 10 min of waiting. Experiments for each fly were performed for at least 1 day, resulting in several hundreds of probing trials in each fly.

We used the 3D CNN to classify the behavior in post-processing. For each trial, we computed the actual elapsed time that the fly was immobile (either in the ‘stop’ state or the ‘proboscis’ state) before the arousal threshold probing, to remove grooming events. Only trials with prior times of complete immobility larger than 5 s were considered; therefore, the resulting times of immobility ranged from 5 s to 5 min. During the probing period in each trial, we obtained the required laser power to awaken the fly. The fly was considered awake when it showed walking, discomfort or grooming behaviors (obtained from the 3D CNN classification). An example of a trial for arousal threshold probing is shown in Extended Data Fig. 3a.

We computed the distribution of IR laser powers required to awaken the fly before three different time intervals of immobility: [5, 35], [35, 200] and [200, 305] seconds. These distributions are shown in Extended Data Fig. 3b–g for individual flies, and in Fig. 2h for all flies. Statistical significance was assessed using a *t*-test (*P* value < 0.05). As shown for individual flies, as well as for all flies (Fig. 2h and Extended Data Fig. 3b–g), longer times of immobility required higher IR laser powers to awaken the flies, confirming that the arousal threshold increases with time of immobility during the long-term recordings.

### Proboscis extension during sleep

We used the trained CNN to classify the behavior of 21 flies during long-term imaging sessions, which included recordings from EG, dFB and R5 neurons. Based on this classification, we identified periods of immobility, defined as the fly either being in the ‘stop’ state or engaged in proboscis extension (‘proboscis’ state). We excluded bouts of immobility lasting less than 1 min and computed the distribution of proboscis extension events in 30-s intervals during sleep. Figure 2i shows the average distribution of proboscis extension events across all sleep bouts for the 21 flies. For each time bin, we required at least ten sleep bouts to be included in the average. Because we did not find more than ten sleep bouts with more than 20 min of uninterrupted immobility, the distribution of proboscis extension events in Fig. 2i is limited to the first 20 min of sleep. We also computed the inter-PE interval, defined as the time between two consecutive proboscis extension events (Fig. 2j), which shows a peak at around 3 s, as previously described^13^.

#### Fitting of fluorescence traces during ‘active’ and ‘rest’ states

Sleep is commonly identified in flies as bouts of immobility that last for at least 5 min, a threshold that has also been used for assessing sleep in tethered walking flies^77,78^. However, shorter bouts of quiescence already show many of the characteristics of sleep^79^. The distribution of sleep bouts, defined as bouts of at least 1 min of immobility during long-term recordings, in EG, AL, R5 neurons and dFB neurons is shown in Supplementary Fig. 2a,b for the day and night, including a total of 21 flies. We compared these distributions to sleep bout distributions in freely moving flies using a filter size of 0 min to define sleep (Fig. 1c,d). During the day, the distribution of sleep bouts in flies during imaging compared to freely moving flies are highly similar (statistically different as assessed with a Kolmogorov–Smirnov test (*P* value < 0.05; Supplementary Fig. 2a). During the night, they are not significantly different (Supplementary Fig. 2b). We also confirmed that around 80% to 90% of sleep bouts in flies during imaging and freely moving flies are shorter than 5 min (Supplementary Fig. 2c).

The amounts of sleep according to a 5-min threshold, only distinguishing between walking and stopping, and according to the ‘rest’ and ‘active’ states (used in Fig. 1i) are compared in Supplementary Table 3.

To find epochs of at least 10 min during which the fly was walking most of the time (active) or sleeping most of the time (rest), first, walking velocity on the ball (with a diameter of 3 mm) was averaged over 1 s. Then, velocities below a threshold of 0.25 body lengths per second (body length defined again as 2.5 mm) were set to zero to remove tracking noise, and we defined a binary walk state of the fly, *w*(*t*), when the thresholded velocity was nonzero (*w*(*t*) = 1). We defined active and rest states where the fly was walking or standing still most of the time. To calculate these states, we used a walk density, which was obtained by low-pass filtering of the walk state with a period of 0.1 h (6 min). Active or rest states were defined based on the walk density being above or below a threshold of 0.5, respectively (Figs. 1i and 2a and Supplementary Figs. 3, 4, 5, 12, 26, 30, 33, 34, 38 and 39). We subdivided the normalized fluorescence of each recording (equation (1)) into *N*_*a*_ traces of at least 10 min of continuous active epochs, ${F}_{active}^{i}(t)$ for *i* = 1, …*N*_*a*_. At least four traces were used from each fly to obtain the normalized averaged fluorescence over active epochs, which was fitted with an exponential (Figs. 1j and 2b, Extended Data Fig. 7c and Supplementary Figs. 6a–e, 7a–e, 8a–e, 13a–d, 27a–d, 31a–d, 35a–h and 40a–g). The exponential was defined by equation (2):

2 $${\hat{F}}_{\mathrm{active}}(t)={A}_{\mathrm{active}}\left(1-{e}^{-t/{\tau }_{\mathrm{active}}}\right)+{C}_{\mathrm{active}}.$$

Here, *A*_active_ is the saturation level, *C*_active_ is an offset and *τ*_active_ is the time constant.

Fluorescence traces for the rest state were similarly selected as epochs of at least 10 min. At least four normalized fluorescence traces were used to obtain the normalized average fluorescence over rest epochs, which was fitted with an exponential (Figs. 1k and 2b, Extended Data Fig. 7d and Supplementary Figs. 6a–e, 7a–e, 8a–e, 13a–d, 27a–d, 31a–d, 35a–h and 40a–g). The exponential was defined by equation (3):

3 $${\hat{F}}_{\rm{rest}}(t)={A}_{\rm{rest}}{e}^{-t/{\tau }_{\rm{rest}}}+{C}_{\rm{rest}}.$$

Here, *A*_rest_ is the amplitude, *C*_rest_ is an offset and *τ*_rest_ is the time constant of the decay. The time constants during active and rest states for activity in EG, AL and extracellular glutamate are shown in Figs. 1l and 2c and Extended Data Fig. 4c.

### Circadian and homeostatic modulation of calcium in glia

We observed 24-h calcium fluctuations associated with circadian activity in AL and EG when analyzing the averaged normalized calcium activity over 24 h (Fig. 2d,e,g). To distinguish between homeostatic and circadian contributions in calcium activity, we first applied a 48-h low-pass filter to eliminate bleaching effects. Next, we used a 12-h high-pass filter to capture fast calcium modulations related to sleep homeostasis and a 12-h low-pass filter to isolate circadian modulation. The contributions of each modulation type were calculated by comparing the ranges of the filtered signals to the range of the original signal, obtaining the contribution to calcium activity as a percentage (Fig. 2f).

### GCaMP sensor saturation

Calcium activity follows exponential saturation dynamics during walking behavior. To determine if this saturation is caused by glia calcium dynamics or if it is an artifact of GCaMP sensor saturation, we analyzed calcium activity during active bouts in the EB and FB of EG (Fig. 1i and Supplementary Fig. 3) and AL (Supplementary Fig. 12). We used the rising exponentials fitted to the normalized fluorescence data of active bouts (see section above) and calculated the saturation levels (Supplementary Fig. 9a). Next, we computed the distribution of saturation levels across all active bouts for EG (Supplementary Fig. 12b) and AL (Supplementary Fig. 12c). The distributions show that a large fraction of saturation levels are below 1 (the maximum normalized fluorescence value), indicating that calcium saturates during some active periods at values lower than the maximum value of the sensor. This confirms that the saturation observed is due to calcium dynamics, not sensor limitations.

#### Velocity modulation of EG activity

To assess the influence of fly velocity at short timescales on calcium activity of EG in the EB and FB, we computed the mean velocity of the fly during each 1-s imaging epoch, and used a high-pass filter to calculate fluorescence oscillations below periods of 0.5 h. Supplementary Fig. 19a shows the mean velocity of fly 1 in each trial (black) as well as the high-pass-filtered fluorescence in the EB (green) and FB (blue). Each data point in Supplementary Fig. 19a was separated by the time difference between consecutive imaging recordings of 60 s. We computed the change in mean velocity and the change of *ΔF/F* in the EB and FB between consecutive epochs (Supplementary Fig. 19b–g). We fitted a linear regression (Supplementary Fig. 19b–g) and computed the Pearson’s correlation between the change in velocity and change in *ΔF/F* for the EB and FB, finding a positive correlation for each fly with *P* values lower than 0.05 (Supplementary Fig. 19i and Supplementary Table 4).

#### Fast calcium modulations in EG

To investigate calcium dynamics during the transition between wakefulness and sleep, we recorded calcium activity in EG from the FB at a high temporal resolution (60 Hz) while applying mechanical stimulation by opening and closing the ball air valve every second. Figure 3g shows two examples of this experiment—calcium increases during stimulation when the fly was already walking, and a rapid calcium peak when the fly was asleep before stimulation. This phenomenon was observed across ten trials from seven flies, with a significant positive correlation (*P* < 0.05) between the amplitude of the calcium peak and the duration of rest before stimulation (Fig. 3h).

#### Correlation between ‘convolved walk’ and calcium activity

To compare how EG calcium activity integrates wake time in the different compartments, we first computed the convolution of the binary ‘walk’ state of the fly, *w*(*t*) (assigned with a value of 1 for velocities higher than the threshold and 0 otherwise), with a temporal filter, *T*_f_(*t*). The temporal filter had a triangular shape (Extended Data Fig. 7h) defined by a filter size, *s*_f_, as given by equation (4):

4 $${T}_{f}\,(t)={\left[1-\frac{1}{{s}_{f}}t\right]}_{+}.$$

Here [⋅]_+_ represents a threshold-linear function to ensure only positive values. The convolution between the ‘walk’ state and the triangular filter produced the ‘convolved walk’ (Extended Data Fig. 7h), which exponentially increased when the ‘walk’ state was 1 and exponentially decreased otherwise. The rate of increase and decrease was the same, defined by the filter size *s*_f_. The ‘convolved walk’ can, therefore, be used to compute the Pearson correlation with the normalized fluorescence (as previously described; see equation (1) for different filter sizes). We used a total of 34 temporal filters, ranging from 0 to 60 min in steps of 6 min, and from 1 h to 25 h in steps of 1 h. The average correlation for EG in the EB, the FB, LAL, MBs and ML is shown with colored lines in Extended Data Fig. 2a, while the standard deviation of the correlation from each group is represented by semitransparent colored regions around the average in Extended Data Fig. 2a. Only filter sizes lower than 240 min were considered, as larger filter sizes produced close to zero correlation. We obtained the maximum value of the averaged correlation and its corresponding filter size (Extended Data Fig. 2a) and assessed the statistical difference between these maxima using a *t*-test (*P < 0.05, **P < 0.005). The same analysis was performed for glutamate recordings (Extended Data Fig. 2b).

#### Sleep deprivation

Sleep deprivation was performed for recordings in EG in the EB and FB. Mechanical sleep deprivation was performed in a total of five flies between 3.6 h and 8.2 h, by opening and closing the airstream of the ball repeatedly three times for 6 s (alternating between 1 s closed and 1 s open) every 20 s. This stimulated flies to walk and prevented sleep. Food deprivation was performed for a total of four flies between 4 h and 16 h. Individual recordings for both conditions are shown in Supplementary Fig. 20a,b, where exponentials were fitted for visualization (equation (2)).

For each fly, we computed the ‘stop’ state of the fly (zero velocity) in 1-s bins. We then averaged the ‘stop’ time for all flies over 1.5 h before sleep deprivation and over 8 h of sleep deprivation (Fig. 4b,d). We computed the average of the fluorescence in the EB and FB, which were first normalized through equation (1) (Fig. 4b,d). At least two flies were considered for the average over the sleep deprivation period. The average ‘stop’ time and fluorescence were also computed over 2 h after sleep deprivation (Fig. 4b,d). We then computed the distribution of the mean values of the normalized fluorescence in each fly over 1.5 h before sleep deprivation, over the first 2 h of sleep deprivation, after the first 2 h of sleep deprivation and over 2 h after sleep deprivation (Fig. 4c,e). Statistical significance between these distributions was assessed using a *t*-test (*P* value < 0.05).

We also calculated the distribution of ‘stop’ time after 2 h of sleep deprivation from the ‘stop’ state of each fly, as well as the duration of bouts of immobility that were larger than 5 min (Fig. 4f). For control flies, we used the recordings from Supplementary Fig. 3, which had identical conditions as sleep-deprived flies except for sleep deprivation. We then computed the distribution of ‘stop’ time over 2 h at the time of the day when sleep deprivation finished for the sleep-deprived flies, as well as the distribution of sleep bout duration for all flies (Fig. 4f). Statistical difference between distributions was assessed again using a *t*-test (*P* value < 0.05).

#### Homeostat model

The homeostat model was fitted to the activity of glia in EB, FB, LAL, MBs and ML, as well as the activity of R5 neurons, and dFB neurons. For model fitting, fluorescence was filtered and normalized as described before (equation (1)). In the homeostat model, we did not distinguish between immobility and sleep, but we distinguished two behavioral states based on the fly’s walking activity for charging and resetting of the homeostat: ‘walk’ and ‘stop’, respectively. The homeostat model was fitted over the time range of each experiment, defined by [*T*_min_, *T*_max_] for ‘stop’ and ‘walk’ periods of the fly. The ‘stop’ behavior of the fly, *s*(*t*), was set to 1 when the velocity of the fly was below a threshold of 0.25 body lengths per second (to remove tracking noise level) and to 0 otherwise. Conversely, the ‘walk’ state of the fly, *w*(*t*), was assigned a value of 1 for velocities higher than the threshold and 0 otherwise (Fig. 4h,i and Supplementary Figs. 21–23, 32–34, 42 and 43). The time resolution for distinguishing between ‘stop’ and ‘walk’ was 1 s. The model used for fitting is shown in equation (5):

5 $${\dot{h}}_{v}(t)=s(t)\frac{1}{{\tau }_{s}}\left(-{h}_{v}(t)+L(t)\right)+w(t)\frac{1}{{\tau }_{w}}\left(-{h}_{v}(t)+U(t)\right).$$

Here, *h*_*v*_(*t*) describes the fluorescence signal (*ΔF*/*F*) or homeostat, while *s*(*t*) and *w*(*t*) act as binary weights for each behavior. Therefore, only one behavioral state contributes to the homeostat at any given time. *τ*_*s*_ and *τ*_*w*_ are the time constants for the stop and walk states, and *L*(*t*) and *U*(*t*) are functions that describe the lower and upper bounds of the homeostat, respectively (Fig. 4h,i and Supplementary Figs. 21–23, 32–34, 42 and 43). These bounds were allowed to vary with time to take into account slow modulations in fluorescence that are unavoidable during long-term imaging recordings, such as photobleaching, circadian modulation or slow changes in calcium levels potentially due to phototoxicity. These changes affect both baseline levels as well as the dynamic range of fluorescence signals over time. Therefore, these upper and lower bounds allowed correction for dynamic range and baseline changes, and were defined as Bezier curves according to equation (6):

6 $$\left\{\begin{array}{l}U(t)=\mathop{\sum }\limits_{k}^{K}{C}_{k}{u}_{k}\quad \\ L(t)=\mathop{\sum }\limits_{k}^{K}{C}_{k}{l}_{k},\quad \end{array}\right.\quad \,\text{where}\,\quad {C}_{k}=\left(\begin{array}{r}K\\ k\end{array}\right){\left(\frac{{T}_{\mathrm{max}}-t}{{T}_{\mathrm{max}}-{T}_{\mathrm{min}}}\right)}^{K-k}{\left(\frac{t-{T}_{\mathrm{min}}}{{T}_{\mathrm{max}}-{T}_{\mathrm{min}}}\right)}^{k}$$

These curves were fitted at the same time as the parameters of the model. The Bezier curves were parameterized by points separated by at least 6 h, which defined the number of parameters *K* for each experiment as shown in equation (7):

7 $$K=\left\lfloor \frac{{T}_{\mathrm{max}}-{T}_{\mathrm{min}}}{6\,\,\text{h}\,}\right\rfloor ,$$

where ⌊ ⋅ ⌋ indicates the floor division operation. For each experiment, we fitted the two time constants *τ*_*s*_ and *τ*_*w*_, and the *K* upper and lower bound parameters, *u*_*k*_ and *l*_*k*_, as shown in equation (8):

8 $$\left\{{\tau }_{s},{\tau }_{w},{u}_{1},\ldots ,{u}_{K},{l}_{1},\ldots ,{l}_{k}\right\}.$$

The fitting procedure is described in the next section. The fitted homeostat model for ensheathing glia activity is shown in Fig. 4h and Supplementary Fig. 21 for EB and FB, Supplementary Fig. 22 for LAL and Supplementary Fig. 23 for MBs and ML; for AL in Fig. 4i and Supplementary Fig. 24; for R5 neurons in Supplementary Figs. 32–34; and for dFB neurons in Supplementary Figs. 42 and 43. All the fitted time constants for stop and walk states and their estimated errors are shown on the left side of all the mentioned Supplementary Figures, and the combined time constants for glia are shown in Fig. 4k for EB and FB and Extended Data Fig. 2c for EB, FB, LAL, MBs and ML, where statistical significance (*P* values < 0.05) between the walk and stop states in EB (green) and FB (blue) was assessed using using a *t*-test.

#### Three-state homeostat model

This model was only fitted for glial activity in the EB and FB, where fluorescence was filtered and normalized as described before (equation (1)). In this model, we distinguished two states for resetting the homeostat: a ‘stop’ state where the fly was at rest (as assessed by ball velocity) for less than 5 min, and a ‘sleep’ state, where the fly was at rest for epochs lasting more than 5 min, as shown in equation (9):

9 $$f(t)=\left\{\,\text{Stop}\,(t),\,\,\text{Sleep}\,(t),\,\,\text{Walk}\,(t)\right\}$$

The model used for fitting is shown in equation (10):

10 $$\dot{{h}_{s}}(t)=\mathop{\sum }\limits_{i}^{{N}_{s}}{f}_{i}(t)\frac{1}{{\tau }_{i}}\left(-{h}_{s}(t)+F({w}_{i},t)\right).$$

Here, *h*_*s*_(*t*) describes the fluorescence signal (*ΔF*/*F*) or homeostat, and *f*_*i*_(*t*) acts as a mask for each state; therefore, only the assigned behavior contributes to the homeostat at any given time (similar to the two-state model). *τ*_*i*_ is a time constant for each behavior *i*, and *w*_*i*_ is the weight with which each behavior contributes to the homeostat, −1 for ‘stop’ and ‘sleep’ states and +1 for ‘wake’. *F* is a function that describes the upper and lower bounds of the homeostat, as shown in equation (11):

11 $$F\left[{w}_{i},t\right]=\left\{\begin{array}{ll}U(t)\quad &\,\text{if}\,\,{w}_{i}=1\\ L(t)\quad &\,\text{if}\,\,{w}_{i}=-1,\end{array}\right.$$

where functions *U*(*t*) and *L*(*t*) are the upper and lower bounds defined in equation (6), similarly to the previous homeostat model.

For each model we fitted the *N*_*s*_ time constants *τ*_*i*_ and the *K* upper and lower bound parameters, *u*_*k*_ and *l*_*k*_, as shown in equation (12):

12 $$\left\{{\tau }_{1},\ldots ,{\tau }_{{N}_{s}},{u}_{1},\ldots ,{u}_{K},{l}_{1},\ldots ,{l}_{k}\right\}$$

An example of model fitting for glial activity in the EB and FB is shown in Supplementary Fig. 28a. The fitted time constants for each of the states, as well as their estimated errors in the EB and FB, are shown in Supplementary Fig. 28b. The distribution of time constants for each state in the EB and FB is shown in Supplementary Fig. 28c. Only time constants with an estimated error lower than 0.2 times its fitted value were included to discard estimated time constants with high error. In Supplementary Fig. 28c, statistical significance (*P* < 0.05) between different behaviors in EB (green) and FB (blue) was assessed using a *t*-test.

We also asked if the homeostat would reset differently after 5 min of immobility. For this purpose, we defined a sleep state starting only after the fly was stopped for 5 min and we fitted equation (10) again. Supplementary Fig. 29a shows an example of glial activity in the EB and FB, together with the fitted time constants in Supplementary Fig. 29b. The distributions for time constants with an estimated error lower than 0.2 times its value are shown in Supplementary Fig. 29c for the EB and FB. We did not find statistical significance between the time constants of the ‘sleep’ and ‘stop’ states using a *t*-test.

#### Seven-state homeostat model

Activity of glia in the EB and FB, which was filtered and normalized as described before (equation (1)), was fitted to a homeostat model using the classified behaviors (an example is shown in Extended Data Fig. 5b). We fitted the dynamics of the homeostat with a model taking into account the *N*_*b*_ = 7 classified behaviors from the 3D CNN, as shown in equation (13):

13 $$\begin{array}{l}b(t)=\left\{\,\text{Stop}\,(t),\,\,\text{Proboscis}\,(t),\,\,\text{Walk}\,(t),\,\,\text{Discomfort}\,(t),\,\,\text{Grooming front}\,(t),\,\,\right.\\\left.\qquad\quad\text{Grooming back}\,(t),\,\,\text{Feeding}\,(t)\right\}.\end{array}$$

Each behavior, *b*_*i*_(*t*), was assigned a binary value, *b*_*i*_(*t*) ∈ {0, 1}, and only one behavior had value 1 at any given time, corresponding to the maximum class value predicted by the 3D CNN. The time resolution for the classification of behavior was 1 s.

We used the following model to fit the data, as shown in equation (14):

14 $$\dot{{h}_{b}}(t)=\mathop{\sum }\limits_{i}^{{N}_{b}}{b}_{i}(t)\frac{1}{{\tau }_{i}}\left(-{h}_{b}(t)+F({w}_{i},t)\right).$$

Here, *h*_*b*_(*t*) describes the fluorescence signal (*ΔF*/*F*) or homeostat, and *b*_*i*_(*t*) acts as a mask for each behavior; therefore, only the assigned behavior contributes to the homeostat at any given time (similar to the two-state model). *τ*_*i*_ is a time constant for each behavior *i*, and *w*_*i*_ is the weight with which each behavior contributes to the homeostat, which can take only two values: *w*_*i*_ ∈ { −1, 1}. When a weight is *w*_*i*_ = −1, the homeostat decreases while the fly performs the behavior, while with *w*_*i*_ = 1, the homeostat increases. *F* is a function that describes the upper and lower bounds of the homeostat, given by equation (11), similarly to the previous two-state and three-state models.

Fitting this differential equation requires determining for each behavior whether it charges or resets the homeostat, that is, contributes with a negative or positive weight to the model. To determine the weight of each behavior in the model, we used fluorescence traces from flies that performed one of the seven behaviors for at least two consecutive imaging epochs (120 s). For ‘discomfort’, we used fluorescence traces recorded when the ball was stopped (Fig. 3c and Supplementary Fig. 17), as this behavior was not observed continuously for 120 s when the ball was free to rotate. For the rest of the behaviors, we used fluorescence traces from the recordings of EG in six flies. For each behavior, all traces were aligned at the origin (Extended Data Fig. 5a for FB) and linear regression was used to determine the slope. If the slope was positive for a behavior, the weight was set to 1 and otherwise to −1 (negative slope). We repeated the same procedure for the FB and found the same weights as in the EB (Extended Data Fig. 5a). For each model, we fitted the *N*_*b*_ time constants *τ*_*i*_ and the *K* upper and lower bound parameters, *u*_*k*_ and *l*_*k*_, as shown in equation (15):

15 $$\left\{{\tau }_{1},\ldots ,{\tau }_{{N}_{b}},{u}_{1},\ldots ,{u}_{K},{l}_{1},\ldots ,{l}_{k}\right\}.$$

Models were fitted independently to EB and FB data.

The fitting procedure is explained below. Since some behaviors were very rare in some flies, the estimation error for model fitting was large for some of the time constants. Therefore, only time constants with an estimated error lower than 0.2 times its fitted value were included in the distribution of the time constants for each behavior in Supplementary Fig. 25a. In Supplementary Fig. 25a, statistical significance (*P* < 0.05) for behaviors in EB (green) and FB (blue) was assessed using a *t*-test.

#### Hunger–walk model

We analyzed the dynamics of dFB activity with respect to feeding by fitting exponentials (see below for a more detailed model). The average fluorescence traces were divided into two epochs, one where the fly was hungry (defined as 3.5 h before the next feeding event) and one where the fly was fed (defined as 30 min after feeding; Fig. 5a,b, Supplementary Fig. 46 and Methods). The time constants during epochs where the fly was hungry were in the order of hours (Fig. 5c). The time constants for resetting after feeding were in the range of minutes, but they might not reflect the actual dynamics of dFB neurons after feeding since resetting depended also on the fly behavioral state (Extended Data Fig. 8).

For a more detailed model-based analysis, fluorescence traces were first filtered and normalized as described before (equation (1)). The model contained two different components that contributed differently: a hunger component, and a walk–stop component. The hunger component was fitted based on the time relative to the feeding events. We defined a ‘hungry’ variable, hungry(*t*), which described whether the fly was hungry or not, and was set to 0 between a feeding event and a time after feeding, called satiated time *t*_satiated_, and set to 1 after the satiated time and the next feeding event (equation (16)).

16 $$\,\text{hungry}\,(t)\,=\left\{\begin{array}{ll}0\quad &\,\text{if}\,\quad t-{t}_{\rm{feeding}} < {t}_{\rm{satiated}}\\ 1\quad &\,\text{if}\,\quad t-{t}_{\rm{feeding}} > {t}_{\rm{satiated}}\end{array}\right.$$

The complementary variable, fed(*t*), was defined as 1 after feeding and before the satiated time, and as 0 otherwise. Therefore, fed(*t*) = 1 − hungry(*t*). The satiated time, *t*_satiated_, interpreted as the time during which the hunger component resets and stays low, was fitted as a model parameter. The hunger homeostat, or hunger component, *h*_hunger_, was, therefore, fitted using the differential equation (17):

17 $$\begin{array}{l}\dot{{h}_{\mathrm{hunger}}}(t)=\,\text{hungry}\,(t)\frac{1}{{\tau }_{\mathrm{hunger}}}\left(-{h}_{\mathrm{hunger}}(t)+U(t)\right)\\\qquad\qquad+\,\text{fed}\,(t)\frac{1}{{\tau }_{\mathrm{fed}}}\left(-{h}_{\mathrm{hunger}}(t)+L(t)\right).\end{array}$$

Here, hungry(*t*) and fed(*t*) act as a mask and, therefore, only the assigned variable contributes to the hunger component at any given time (similarly to the two-state model). *τ*_hungry_ and *τ*_fed_ are the time constants that describe the increase and decrease in the hunger component, respectively. The hunger component and its parameters are shown in Fig. 5f. *U*(*t*) and *L*(*t*) represent the upper and lower bounds, defined in equation (6), which were fitted together with the parameters. The walk–stop component, *h*_*v*_(*t*), was defined as in the homeostat model (equation (5), but the upper and lower bounds were set constant, *U*(*t*) = 1 and *L*(*t*) = 0.

Finally, the hunger–walk model was defined as the weighted sum of the hunger and walk–stop components as shown in equation (18):

18 $${h}_{\mathrm{hunger-stop}}={h}_{\mathrm{hunger}}+{w}_{v}{h}_{v}(t)$$

where *w*_*v*_ was a weight that indicated the contribution of the walk–stop component, which was also fitted. In summary, for this model we fitted the following six parameters, as shown in equation (19):

19 $$\left\{{\tau }_{\mathrm{hungry}},{\tau }_{\mathrm{fed}},{t}_{\mathrm{satiated}},{\tau }_{w},{\tau }_{s},{w}_{v}\right\},$$

as well as the parameters that defined the upper and lower bounds of the hunger component, *u*_*k*_ and *l*_*k*_ from equation (6). The fitting procedure is explained in the next section, and the fitted models are shown in Fig. 5g and Supplementary Figs. 47 and 48. The fitted time constants of all flies are shown in Fig. 5h, where only parameters with an estimated error lower than 0.2 times their fitted value were included. In Supplementary Fig. 5h,i, statistical significance (*P* < 0.05) for behaviors in EB (green) and FB (blue) was assessed using a *t*-test.

#### Model fitting

Fitting of models was performed using the function ‘curve fit’ in SciPy^80^ in Python. Given a set of initial values of the parameters, we integrated each model with the corresponding equation, for example, using equation (5) for the two-state model, equation (10) for the three-state model and equation (14) for the seven-state model, from their corresponding behavioral states (‘stop’, ‘walk’, …) over the experiment timeline using Euler integration (with 1-s steps). Since the fluorescence data were not recorded continuously, we then interpolated the values of the model in agreement with the times of the recordings. Finally, we obtained an *L*_2_error function between the interpolated model values and fluorescence data. The parameter values were updated iteratively from the Jacobian of the error function, and the minimum was found using the trust region reflective algorithm as the optimization method.

To prevent parameters from taking forbidden values during optimization, we used parameter bounds. For the homeostat model, as well as the three-state and seven-state homeostat models, the allowed range for time constants was [0, 2] hours. For the hunger–walk model, time constants in the range of [0, 2] hours were allowed, except for the time constant for ‘hungry’, *τ*_hungry_, where the allowed range was [0, 6] hours. The allowed range for the weight *w*_*v*_ in the hunger model was [0, 1], and the allowed ranges for the upper and lower bound parameters, *u*_*k*_ and *l*_*k*_, were [− 1, 0] and [0, 2] (normalized *ΔF*/*F*), respectively.

We computed the error or variance of each parameter from the covariance matrix between all parameters, which was returned by the function ‘curve fit’. This error indicated one standard deviation error of the parameter, obtained as the square root of the corresponding diagonal element in the covariance matrix. In all models, parameters with errors lower than 0.2 times the magnitude of the fitted parameter were considered for distribution analysis (Figs. 1h and 4k and Supplementary Figs. 25a, 28 and 29).

#### Comparison between models

We asked whether the seven-state homeostat model fitted the glial activity better than the homeostat model. In this case, the homeostat model is a nested model, the seven-state homeostat model, which means that the homeostat model and its parameters are included in the seven-state homeostat model and parameters. Therefore, we asked if a null hypothesis holds, which establishes that the more complex (seven-state homeostat) model does not fit fluorescence data significantly better than the simpler model (homeostat model). For this purpose, we used an *F*-test^81^. Because the more complex model has different numbers of parameters than the simpler model, we can compute the *F*-statistic of the *F*-test as shown in equation (20):

20 $${F}-{\mathrm{statistic}}\,=\frac{\left({R}_{\mathrm{simple}}-{R}_{\mathrm{complex}}\right)/\left({N}_{\mathrm{complex}}-{N}_{\mathrm{simple}}\right)}{{R}_{\mathrm{complex}}/\left.\right(\!{N}_{F}-\left({N}_{\mathrm{complex}}\right)},$$

where *R*_simple_ and *R*_complex_ are the residual sum of squares for the simpler and the more complex model, respectively (same as *L*_2_error), *N*_simple_ and *N*_complex_ are the number of parameters for the simpler and more complex model, respectively (in this case *N*_complex_ − *N*_homeostat_ = 5) and *N*_*F*_ is the number of fluorescence points that was used for fitting both models. The *F*-statistic value can then be used to generate a *P* value, rejecting the null hypothesis stated above if it is lower than 0.05. If the null hypothesis is rejected, we can conclude that the more complex model fits the data significantly better than the simpler model.

The *L*_2_errors between each pair of models for each fly in the EB and FB are shown in Supplementary Fig. 25b, while the *L*_2_errors, *F*-statistic values and *P* values comparing each pair of models in the EB and FB are shown in Supplementary Tables 5 and 6. This analysis concludes that the seven-state homeostat model provides a better fit for the glia data than the homeostat model.

We performed a similar analysis to compare the homeostat model with the hunger–walk model fitted to dFB neurons. As before, the homeostat model was a nested model of the hunger–walk model. We computed the *F*-statistic between the two models (being the homeostat model the simpler model, while the hunger–walk model is the more complex model) from equation (20) and obtained the corresponding *P* values. The *L*_2_errors between each pair of models fitted for each fly is shown in Fig. 5i, while the *L*_2_errors, *F*-statistic values and *P* values are shown in Supplementary Table 7. Because all the *P* values were lower than 0.05, we conclude that the hunger–walk model fits the activity of dFB neurons better than the homeostat model.

#### Piecewise regression of calcium data during immobility

Calcium dynamics can be described with an exponential decay while the fly is at rest (Fig. 1k and Supplementary Fig. 6). We asked whether the rate of calcium reset during rest could help distinguish between rest and sleep, with sleep typically defined as periods of immobility lasting more than 5 min. For exponential functions, the slope is steepest at the beginning and decreases exponentially to zero.

To investigate this, we approximated the exponential calcium dynamics of ensheathing glia in the EB and FB using a piecewise regression with a single breakpoint. This approach fits the data with two linear segments with different slopes and identifies the breakpoint, which is the time at which calcium activity transitions between these two segments.

We used the velocity of the fly at 1-s resolution to identify periods of immobility lasting at least 3 min. We then extracted the corresponding calcium activity for these time periods. To standardize the calcium traces, we subtracted the 0.95 quantile, aligning the traces to approximately zero at their starting point. Extended Data Fig. 6a,b illustrates the fluorescence data over these immobility periods. Linear piecewise regression was fitted to all fluorescence data points using the Python package ‘piecewise regression’^82^. The results are shown in Extended Data Fig. 6a,b, where the red line represents the fitted regression model, and the average fluorescence over time is shown as a black line. The breakpoint is indicated by the orange vertical line. The shaded orange region around the breakpoint represents the 95% confidence interval for its estimation.

Extended Data Fig. 6c shows the distributions of slopes for the first and second segments in the EB and FB, respectively. Asterisks indicate statistically significant differences between the slopes (*t*-test, *P* value < 0.05), with the first segment being more negative than the second. This indicates that calcium resets more rapidly before the breakpoint, as expected for an exponential decay. The distribution of breakpoints in the FB and EB is shown in Extended Data Fig. 6e. These results reveal that calcium resets more quickly during the first 4–6 min of immobility, as defined by the breakpoint, and slows down afterwards. The estimated breakpoint at 4–6 min of immobility defines a transition in the rate of calcium reset, potentially corresponding to the a transition between different sleep states (see ref. ^22^ for a recent discussion).

#### Comparison between activity of EG and neurons

The dynamics of activity in EG, R5 and dFB neurons were compared with respect to how well they described a sleep homeostat. For this, we used three different methods. In a first approach, we reasoned that a homeostatic signal should increase over active epochs, producing a positive correlation with the time during which the fly was active, and decrease over epochs of rest or sleep, thus producing a negative correlation with time spent resting or sleeping. Therefore, we computed the Pearson correlation between the averaged normalized traces over 30 min of active and rest epochs (Fig. 1j,k, Extended Data Fig. 7c,d and Supplementary Figs. 6, 31, 35 and 40). We distinguished between EB and FB neuropils and compared the distributions of correlation values in the EB between glia and R5 neurons and the distribution of correlation values in the FB between glia and dFB neurons (Extended Data Fig. 7e,f). To assess whether the distributions of correlation values were statistically different, we used a *t*-test.

Alternatively, we compared the normalized average traces to a linear fit (Fig. 1j,k, Extended Data Fig. 7c,d and Supplementary Figs. 6, 31, 35 and 40) during the first 30 min of rest and activity. The slope of this fit for each fly (Supplementary Fig. 41) represents how fast activity increases toward a maximum value during active epochs (Supplementary Fig. 41) and how fast activity decreases during epochs of rest to baseline (Supplementary Fig. 41). Statistical differences in the slope distributions were again assessed using a *t*-test and are represented by asterisks (*P* value < 0.05; Supplementary Fig. 41). The slope for a homeostatic signal that increases and decreases linearly over 30 min of activity or rest should be 1 and −1, respectively. Glial activity was very close to these values (Supplementary Fig. 41), while the slope for dFB neurons during rest was close to zero.

As a second approach, we calculated the *L*_2_error of the homeostat model fitted for glia and R5 neurons. We again distinguished between neuropils and compared the *L*_2_error of glia in the EB and of R5 neurons, as well as glia in the FB and dFB neurons (Extended Data Fig. 7g). Statistical significance was assessed using a *t*-test (*P* < 0.05).

As a third approach, we computed the Pearson correlation between the normalized fluorescence and the ‘convolved walk’ (Extended Data Fig. 7h) for different filter sizes (equation (4)), as described in ‘Correlation between ‘convolved walk’ and calcium activity’. A total of 34 temporal filters were used, ranging from 0 to 60 min in steps of 6 min, and from 1 h to 25 h in steps of 1 h. The average correlation for glia in the EB and R5 neurons, and for glia in the FB and dFB neurons, is shown in Extended Data Fig. 7i, while the standard deviation of the correlation from each group is represented by semitransparent colored regions around the average in Extended Data Fig. 7i. Filter sizes were limited to 240 min, because they produced close to zero correlation beyond that size. The maximum value of the averaged correlation was obtained together with its corresponding filter size (Extended Data Fig. 7i). Statistical significance between these maxima was assessed using a *t*-test, indicated by asterisks in Extended Data Fig. 7i (*P* values < 0.05).

#### Fitting of fluorescence traces during ‘hungry’ and ‘fed’ states

This analysis was only performed for dFB neurons and was similar to the analysis using active and rest epochs. We defined ‘hunger’ and ‘fed’ epochs to characterize the trend of activity of dFB neurons. Fed epochs were defined over the first 30 min after a feeding event, while hungry epochs were defined as starting 30 min after a feeding event and lasting until the next feeding. At least four normalized fluorescence traces were used to obtain the normalized average fluorescence over hungry or fed states, which was fitted with a rising exponential (equation (2)) or a resetting exponential (equation (3)), respectively (Fig. 5b and Supplementary Fig. 46). The time constants of the fitted exponentials are shown in Fig. 5c, where the asterisk indicates statistical significance (*P* value < 0.05) using a *t*-test.

#### Change in body size before and after feeding

This analysis was only performed for dFB neurons. We asked whether the reset of activity of dFB neurons was linked with how much food flies were ingesting at each feeding event. Therefore, we used the side view of the fly in the video recorded during the experiment 10 min before feeding and 10 min after feeding. Using color segmentation in OpenCV, we obtained a mask of the fly that only included the abdomen and the wings (Fig. 5d). We did not include the legs or the head in this mask, as flies could, for example, move the legs or extend the proboscis, producing an enlarged body size. We computed the area of the mask before and after feeding, *A*_before_ and *A*_after_, respectively, and calculated the relative body size change between feeding events, *ΔA*_*r*_, as given by equation (21):

21 $$\Delta {A}_{r}=\frac{{A}_{\rm{after}}-{A}_{\rm{before}}}{{A}_{\rm{before}}}$$

The relative body size changes between feeding events were then compared to the corresponding differences in activity of dFB neurons before and after feeding. For this, we averaged the activity of dFB neurons over the last 10 min before a feeding event, < *F* *>*_before_, and over the next 10 min after feeding, < *F* > _after_, and obtained the difference, < *ΔF* > = < *F* > _before_ − < *F* > _after_. We obtained a total of 107 pairs of relative body size changes and fluorescence changes from eight flies (Fig. 5e). We computed the Pearson correlation between all pairs of points, as well as during the first half of the lowest relative body size changes (50% quantile) and the second half (Fig. 5e). While the correlation was positive and statistically significant (*P* value < 0.05), this correlation was stronger for lower relative body size changes (Fig. 5e, see Supplementary Data 1 for details on statistics).

#### Electron microscopy data analysis

We downloaded a portion of the raw electron microscopy (EM) data from the hemibrain dataset^40,83^. The analyzed data, containing the EB and FB, was a cube with dimensions of 136 μm, centered at the coordinates (25711, 27000, 19798). Although the hemibrain dataset contains the segmentation of glia matter and tracheal tubes, we noticed that a custom glia mask for each slice, obtained by selecting those parts of the image where neurons were not segmented, resulted in more reliable results. Therefore, we used the available segmentation of neurons in each slice and defined the glia matter as the non-segmented regions. An example of this approach for the segmentation of glia matter for one slice is shown in Fig. 6a.

Tracheal tubes appear in a different color, and we used a simple color threshold segmentation method. Once all slices were segmented, mislabeled non-tracheal structures were manually removed using the software Blender. Figure 6a shows the segmentation of the tracheal tubes for one slice. Finally, we used the available hemibrain-defined neuropil boundaries to get EB and FB masks (Fig. 6a).

To see whether tracheal tubes were always found inside glia, we computed the overlap between each of the tracheal tubes and the glia matter for each slice. If the overlap was nonzero, the tracheal tube was considered to be inside the glia mask. Figure 6b shows the amount of tracheal tubes inside (green) and outside (red) glia for each slice (left side), and the distribution of tracheal tubes across all slices inside and outside glia (right side). Statistical significance between tracheal tubes inside and outside glia was addressed using the Kolmogorov–Smirnov test.

To compute the distance between tracheal tubes and the boundaries of EB and FB neuropils, we generated a 3D distance transform for each neuropil using Python’s ‘edt’ library. This process produced a 3D distance map for each neuropil, which is a 3D image where the voxel values indicated the distance to the nearest neuropil boundary. Voxels located outside the neuropil had positive distance values, while those inside had negative values. Next, for each slice, we identified the center of each tracheal tube using the tracheal mask and determined its distance to EB and FB neuropils based on its position in the 3D distance map. After calculating the distances of the tracheal tubes for each slice, we grouped them into 1-μm bins to create a distribution of the number of tracheal tubes for each binned distance. The bottom panel of Fig. 6c shows this distribution, with slices along the *y* axis and the binned distances along the *x* axis for both EB (left) and FB (right) neuropils. For slices that did not intersect with the neuropil (above or below its boundaries), negative distances (representing positions inside the neuropil) are shown in white. This indicates that negative distances are not possible in these slices, as no part of the neuropil is present. Figure 6c shows the average distribution of tracheal tubes across slices for the EB (left) and the FB (right) that contain at least one tracheal tube. Specifically, for each distance bin, we calculated the average number of tracheal tubes, considering only the slices where tracheal tubes are present. This approach offers a clearer view of the number of tracheal tubes near the boundaries by focusing on slices that contain tracheal tubes. Tracheal tubes were rarely observed inside the neuropil (at negative distances) and were primarily concentrated around its boundary (at zero distance; Fig. 6c). This suggests that the tracheal tubes are embedded in EG, which are located at the neuropil boundary.

#### Gas exposure during imaging

To deliver gas to flies during imaging, we used an outlet at a distance of approximately 1 cm from the fly positioned to the side (Extended Data Fig. 10e). The outlet delivered air, oxygen (100% concentration), CO_2_ (at three different concentrations: 10%, 50% or 100% in air) or ammonia. For ammonia^84^, air was humidified with a solution that contained a concentration of 5% ammonia diluted in water. The CO_2_ concentration was obtained by mixing different volumes of air with CO_2_. A suction outlet running at 1 liter per minute was positioned opposite the inlet, on the other side of the fly and, at a distance of approximately 1 cm from the fly (Fig. 6e). The outlet was connected through a tube to a sensor (SPRINTIR-WF-20 sensor) to measure the concentration of CO_2_ (Fig. 6f).

Switching between the different gases was performed through a system of automated air valves, which were controlled using an Arduino MEGA board. The experiment consisted of delivering first oxygen (for 5 min), then CO_2_ (for 10 min for each different concentration) and then ammonia (for 5 min). Between these gases, air was delivered (Fig. 6f). All gases were delivered at a rate of 0.5 liters per minute.

Flies expressing either jGCaMP8m or pHluorinSE on EG (56F03-GAL4), AL (86E01-GAL4) or neurons (57C07-GAL4) were used during gas exposure experiments (sample size is shown in Fig. 6f). All data are shown in Supplementary Figs. 49–54.

We calculated the percentage of immobile flies during gas exposure in 10-s bins from ball velocity averaged over 1-s bins recorded at 200 Hz (Fig. 6f). We also obtained proboscis extensions per minute from video recorded at 10 Hz (Fig. 6f). The average fluorescence traces from jGCaMP8m and pHluorinSE in neurons, AL and EG are shown in the last three rows of Fig. 6f for FB and Extended Data Fig. 10a for EB (black line for pHluorinSE, green line for jGCaMP8m). We analyzed the distribution of immobility in all flies during and after gas exposure. Immobility was defined as the percentage of time stopped (measured from the ball velocity recorded at 200 Hz and averaged over 1-s bins) during the last 5 min of gas exposure (Fig. 6g) and the first 5 min following gas exposure (Fig. 6h). Immobility was significantly higher during 100% CO_2_ exposure, and after all CO_2_ concentrations and ammonia. Proboscis extension significantly increased during 50% and 100% CO_2_ exposure (Fig. 6i). Statistical significance in Fig. 6h,i was assessed using *t*-tests (*P* < 0.05).

We also computed the average pHluorinSE fluorescence during gas exposure in neurons, AL and EG in the FB (Fig. 6j) and the EB (Extended Data Fig. 10b). The drop in pHluorinSE was largest in EG, followed by AL, and smallest in neurons, reflecting a layered hierarchy where glia are most affected due to proximity to the gas. We also computed the pHluorinSE overshoot 5 min after exposure, showing that both CO_2_ and ammonia induced significant overshoots in EG and AL (Fig. 6k and Supplementary Fig. 6c).

Next, we measured the jGCaMP8m calcium overshoot (Fig. 6l and Supplementary Fig. 6d), revealing significant differences between ammonia and 50% CO_2_. This calcium overshoot is different from the pH overshoot, showing that calcium activity during CO_2_ exposure is not simply a reflection of pH changes. This difference motivated the use of ammonia, as both CO_2_ and ammonia raise pH similarly. However, ammonia did not trigger the strong calcium responses observed with CO_2_, confirming that the jGCaMP8m signal after CO_2_ exposure reflects actual calcium overshoot dynamics in AL and EG. Statistical differences between neurons, AL and glia were assessed using a one-way analysis of variance (Fig. 6i–l and Extended Data Fig. 10b–d).

Finally, we calculated the time constants for the decay of pHluorinSE and jGCaMP8m signals following CO_2_ exposure in EG and AL, both in the EB (Extended Data Fig. 10e–h) and the FB (Extended Data Fig. 10i–l) by fitting an exponential using the ‘curve fit’ function from SciPy^80^. The time constants for both cell types were similar, averaging around 2 min. GCaMP8m corrections were applied to separate calcium-related fluorescence changes from pH-related effects in EG and AL (Fig. 6f). Details of this correction are provided in Supplementary Results 1.5.

#### Detection of proboscis extension during gas exposure and optogenetics

The behavior of flies during optogenetics and gas exposure experiments was recorded using a different camera and different lighting conditions compared to those used in long-term imaging and, therefore, we did not used the previously trained CNN. Instead, we calculated the intensity within a rectangular area near the fly’s proboscis. When the proboscis extended, the intensity in this area increased. We measured this intensity for every video frame during the optogenetic and gas exposure experiments and set a threshold for each fly to determine when the proboscis was extended. Using this approach, we identified proboscis extensions for each fly (Supplementary Figs. 49–54, 56 and 57).

#### Optogenetics during imaging

Optogenetic activation is achieved by illumination of the select areas of the brain with computer-generated holograms, projected with a red laser (640 nm, Toptica, iBEAM-SMART-640-S-HP) and phase SLM (Meadowlark Optics, HSP1920-1064-HSP8). The activation laser beam is expanded, modulated and multiplexed into the scanned fluorescence illumination path with a dichroic mirror. The phase SLM, displaying the hologram, is located in the plane conjugate with the back focal plane of the microscope objective (Fig. 7a).

We used the classic Gerchberg–Saxton algorithm^85^ with 20 iterations, where the desired activation intensity distribution in the FOV serves as the amplitude constraint to find phase modulation in the Fourier plane to be displayed on the SLM. We integrated a custom graphical user interface with ScanImage^86^, allowing the user to mark areas of activation by painting over them in the two-photon FOV. To schedule the activation, the laser was controlled by an Arduino UNO board to switch it on and off automatically.

To allow for precise activation, a correspondence needs to be established between the two-photon FOV and the area illuminated by the SLM. This calibration was done with an auxiliary area scan camera (Basler acA640-750um) imaging reflected light and fluorescence from the sample with beads through the objective (the procedure is shown in Supplementary Fig. 55).

#### Optogenetic protocol during imaging

We crossed flies expressing EG and AL GAL4 lines 56F03-GAL4 and 86E01-GAL4 with UAS-CSChrimson and UAS-jGCaMP8m. We also crossed 56F03-GAL4 with UAS-CSChrimson and UAS-pHluorineSE. Flies were raised on regular food and, after surgery, were placed on retinal food, while control flies remained on regular food for recovery. After a 1-day or 2-day recovery period, experiments were conducted using a power of 0.3 mW in the optogenetic laser. For flies expressing CsChrimson and GcAMP8m, we used a total of six and nine flies for EG and AL optogenetic activation, and eight and nine flies for EG and AL controls, respectively. For flies expressing CsChrimson and pHlourinSE, we used six flies for EG optogenetic activation and six flies for EG controls.

The shape of the pattern used for activation was chosen similar to a bump of activity as observed in columnar neurons in the CX^87^, and the site in the EB most distant from the FB was selected to see whether local activation of the EB would also induce activity in the FB.

Trials involved continuous laser activation for varying durations (30, 60, 120, 300 and 600 s), followed by 20 min of recovery. Before each trial, we mechanically stimulated the flies for 10 s to ensure that they were awake. Optogenetic activation was performed on the lower edge of the EB (as defined in Fig. 7a; all data are shown in Supplementary Figs. 56 and 57). Figure 7b,c shows that flies were less immobile 10 s before activation due to mechanical stimulation. We also measured proboscis extension events per second during the trials (Fig. 7b,c). Calcium dynamics were recorded at the activation site in the EB and in the FB compared to control flies (Fig. 7b,c).

The percentage of immobile flies, calculated in 10-s bins from ball velocity averaged over 1-s bins recorded at 200 Hz during optogenetic activation in EG and AL is shown in Fig. 7b,c. Immobility was defined for all flies as the percentage of time stopped during optogenetic activation (Fig. 7d), and was higher than in control flies. Significant differences compared to control were determined by *t*-tests (*P* < 0.05). Additionally, proboscis extensions per second were significantly greater during optogenetic activation in these cell types (Fig. 7e). The inter-proboscis extension period during activation averaged around 1.5 s, faster than during spontaneous sleep (Supplementary Fig. 58a).

We analyzed calcium dynamics in the EB and the FB during optogenetic activation. Fluorescence signals recorded with GcAMP8m in both EG and AL showed a sharp drop at the beginning of optogenetic activation (Fig. 7b,c), which was produced by a drop in pH, as measured with the pHluorinSE in EG (Fig. 7b). For calcium drift or integration, we calculated the 0.95 quantile fluorescence value for EG and AL (Fig. 7f). We also measured the calcium and pH overshoot in the FB 5 min after activation, defined by the 0.95 quantile calcium value (Fig. 7g for calcium in EG and AL, and Fig. 7g for pH in EG). Both drift and overshoot were significantly different from control (Fig. 7f,g). The overshoot amplitude also increased with longer optogenetic activation times in EG and AL. The same analysis was performed for the EB (Supplementary Fig. 58b,c). Finally, we computed the time constants for calcium decay after the overshoot, revealing that calcium dynamics were faster in AL compared to EG (Supplementary Fig. 58d). Time constants were also similar to those obtained after CO_2_ exposure (Extended Data Fig. 10e–l).

GCaAMP8m correction for pH in EG was not applied during optogenetic experiments, different from gas exposure experiments. This is because GCaAMP8m and pHluorinSE sensors exhibit similar dynamics: both signals decrease during optogenetic activation and then overshoot afterwards. To better disentangle calcium from pH effects in the GCaAMP8m signal, a method would be needed to increase pH without also elevating calcium, as achieved using ammonia exposure in gas experiments. Ammonia exposure helped confirm that GCaAMP8m dynamics were not merely a reflection of pH. However, as ammonia exposure was not conducted alongside the optogenetic experiments, we did not correct the GCaAMP8m signal in this case.

### Statistics and reproducibility

For all experiments except for optogenetic experiments, we used MATLAB R2016b, ScanImage 2018b running on Windows 10, and ROS 1 Noetic with Python 2.7.17 in Ubuntu 18.04LTS. For optogenetic experiments, we used MATLAB R2021a and ScanImage 2021 running on Windows 10, and ROS 1 Melodic with Python 2.7.17 in Ubuntu 20.04LTS. Statistical analyses were conducted in Python 3.9.7 using the packages pingouin (v0.5.5)^88^ and SciPy (v1.7.3)^80^. No statistical method was used to determine the sample size in any of the experiments. Sample sizes were chosen that were similar to those in other studies that recorded large datasets from behaving animals over longer timescales^72,89^. For long-term imaging experiments, data recorded within 48 h after surgery were excluded. All included data are shown in the main figures and supplementary figures, with specific exclusions noted in Supplementary Figs. 14d and 45, along with explanations in the figure legends. For each experiment, flies were randomly selected for surgery. After recovery, they were chosen by the experimenters based on imaging quality in the brain at the focal plane, which varied depending on the quality of the dissection. Animals were assigned to the various experimental groups based on known genotypes. Randomization in the organization of the experimental conditions or stimulus presentation was not performed. Experimenters were blinded to experimental outcomes after the start of the experiment. Data collection and analysis were not performed blindly with respect to fly genotype, as the experimenters needed to select flies with a specific genotype for each experiment. Therefore, data collection and analysis were not performed blind to the conditions of the experiments. Individual data points and traces are shown in the figures. Statistical significance was considered for *P* values less than 0.05. For two-sided *t*-tests, data distributions were assumed to be normal, but this was not formally tested.

### Reporting summary

Further information on research design is available in the Nature Portfolio Reporting Summary linked to this article.

## Online content

Any methods, additional references, Nature Portfolio reporting summaries, source data, extended data, supplementary information, acknowledgements, peer review information; details of author contributions and competing interests; and statements of data and code availability are available at 10.1038/s41593-025-01942-1.

## Supplementary information

Supplementary Information Supplementary Figs. 1–59, Tables 1–7, results and references.

Reporting Summary

Supplementary Video 1 Video for experiment of fly 1. Top, time during experiment. Middle, right, imaging data (60 frames or 1 s average, recorded every 1 min). Rotating blue (during the day) or gray (during the night) arch represents stripe orientation in VR with respect to the fly (the fly’s head and abdomen are pointing toward the upper and lower axis of the video, respectively). Right, side view of the fly on the ball. Behavior classification is shown in white in the left corner. Bottom, velocity of the fly (first row), behavior classification (second row) and glia activity in EB (green) and FB (blue) over time. Gray areas in top and bottom rows indicate night (VR display is off). The time during the experiment at which the movie snippet was extracted is indicated by the green bar.

Supplementary Video 2 Same as Supplementary Video 1 for fly 2.

Supplementary Video 3 Same as Supplementary Video 1 for fly 3.

Supplementary Video 4 Same as Supplementary Video 1 for fly 4.

Supplementary Video 5 Same as Supplementary Video 1 for fly 5.

Supplementary Video 6 Same as Supplementary Video 1 for fly 6.

Supplementary Video 7 Classification of fly behavior using 3D CNN. The video shows examples of classification of different behaviors.

Supplementary Video 8 Example of tracking freely walking flies in behavior setup under IR illumination.

Supplementary Data 1 Statistical information.

## Data Availability

All the behavioral data, raw functional imaging data and associated behavioral data can be made available upon request. The hemibrain dataset is available at https://www.janelia.org/project-team/flyem/hemibrain/.

## Code availability

All the code for data processing, analysis and figure generation can be made available upon request.
