# Author Correction: Dynamics of glia and neurons regulate homeostatic rest, sleep and feeding behavior in *Drosophila*

**DOI:** 10.1038/s41593-025-01984-5

**Published:** 2025-05-06

**Authors:** Andres Flores-Valle, Ivan Vishniakou, Johannes D. Seelig

**Affiliations:** Max Planck Institute for Neurobiology of Behavior – caesar (MPINB), Bonn, Germany

**Keywords:** Sleep, Astrocyte, Microglia, Neural circuits, Feeding behaviour

Correction to: *Nature Neuroscience* 10.1038/s41593-025-01942-1, published online 21 April 2025.

In the version of the article initially published, in Fig. 5a, the velocity trace was shifted during figure preparation and therefore misaligned with the fluorescence trace. This has now been corrected in the HTML and PDF versions of the article.

