# Supplementary material for: Dynamics of glia and neurons regulate homeostatic rest, sleep and feeding behavior in Drosophila
Source: Nat Neurosci. 2025 Apr 21;28(6):1226–40. doi: 10.1038/s41593-025-01942-1 (PMC12148942; doi:10.1038/s41593-025-01942-1)
Supplement: Supplementary file 1 — Supplementary Figs. 1–59, Tables 1–7, results and references. [file 41593_2025_1942_MOESM1_ESM.pdf]

nature neuroscience

Supplementary information

https://doi.org/10.1038/s41593-025-01942-1

Dynamics of glia and neurons regulate homeostatic rest, sleep and feeding behavior in *Drosophila*

In the format provided by the authors and unedited

1 Supplementary Results

1.1 Comparison of homeostat activity in different brain areas

Glia activity in MB and midline showed in some cases weaker correlations with walking activity (Extended Data Fig. 2a and Methods) than the central complex. However, intermittently opening and closing the air stream supporting the ball with a valve every second, which induced fast walking activity, reliably induced increased calcium activity in MB and midline similar to the central complex (Supplementary Figs. S10 and S11). That activity in EB, FB, and LAL was better correlated with walking activity compared to activity in MB and midline (see Methods and Extended Data Fig. 2a), could be due to neurons in EB, FB and LAL being important for visual navigation, the behavioral paradigm used in these experiments.

1.2 Supplementary results for comparison of EG and neuron dynamics

We further analyzed whether a homeostat model as used for EG could fit activity of R5 and dFB neurons (Supplementary Fig. S32, S36, S37, S42, S43, and Methods). However, Extended Data Fig. 7g shows that the L2 error between the fitted model and normalized activity was larger for both neural populations than for glia.

Finally, we computed the convolution between walking activity with a triangular-shaped temporal filter, which produces an exponential increase during 'walk' bouts and a (symmetric) exponential decrease during 'stop' bouts at a rate defined by the filter size (Extended Data Fig. 7h, see Methods). We calculated the correlation between activity in neurons and glia with the corresponding signal resulting from the convolution of different temporal filter sizes with 'walk' (Extended Data Fig. 7i). The correlation with glia activity was high and peaked at a filter size of 24 min, similar to the dynamics obtained above. The correlation for R5 and dFB neurons was positive but lower compared to glia activity (Extended Data Fig. 7i, see Methods).

1.3 Glutamate changes faster than calcium

EG are important for glutamatergic signaling [95] and glutamatergic neurons show activity correlated with walking [96]. We therefore tested whether the slow calcium dynamics observed in glia corresponded to the accumulation of extracellular glutamate. The glutamate sensor iGluSnFR was expressed in EG and we performed long-term imaging experiments as before [97, 98].

Glutamate activity was correlated with walking (Extended Data Fig. 4a, S26, Extended Data Fig. 2b) and we again defined active and rest epochs based on the velocity of the fly and fitted exponential curves for EB and FB fluorescence traces (Extended Data Fig. 4b and S27). Extracellular glutamate increased during active epochs and decreased during rest (Extended Data Fig. 4b). However, time constants were faster than those obtained for calcium (compare Extended Data Fig. 4c and Fig. 1l, and Extended Data Fig. 2a and b), indicating that EG calcium levels do not simply reflect extracellular glutamate concentration.

1.4 Homeostat integrates multiple behaviors

We also tested whether a more detailed analysis, distinguishing multiple behaviors, could improve model fitting. Behavior on the ball, which was monitored with a camera throughout the experiments, was classified into the following 7 categories using machine learning with 1 s time resolution (similar to [99], Supplementary Fig. S16): immobility (stop), proboscis extension, walking, discomfort (where the fly was pushing or pulling the ball), grooming of the front, grooming of the back, and feeding (see Methods and Supplementary Video S7).

We used the intervals where flies performed one of the defined behaviors continuously over consecutive imaging epochs to assess whether they contributed to an increase or decrease of calcium activity. As expected, glia activity in EB and FB decreased when flies were stopped, but also during proboscis extension and grooming. Glia activity increased with walking, feeding (during which flies were often walking), and discomfort (Extended Data Fig. 5a).

We next fitted a model to activity in EB and FB to obtain the time constants for each of the seven behaviors (Extended Data Fig. 5b). This model predicts that resetting of the homeostat occurs faster during proboscis extension and back-grooming than during immobility, and also resets during front-grooming, although more slowly than during immobility. Proboscis extension and back-grooming was only observed in short bouts, and rarely over more than two consecutive imaging epochs (Supplementary Fig. S25a). Consistent with these observations, proboscis extension has been described to occur during a deep sleep state, and other brief movements have also been observed during sleep [100, 101, 102].

1.5 Correction of jGCaMP8 fluorescence for pH induced changes

During CO_2 experiments (Fig. 6f) fluorescence changes in jGCaMP8m can result from both calcium activity and pH changes. To estimate the pH dependence, we plotted jGCaMP8m fluorescence throughout the gas exposure experiment, including oxygen, CO_2, and ammonia exposure (Fig. 6f, green), against corresponding pH measurements from the pHluorinSE sensor (Fig. 6f, black). These data points, shown in Supplementary Fig. 9a in green, includes all recorded flies in EG (all recordings had identical gas exposure protocols). We calculated the median (0.5 quantile) GCaMP8m fluorescence in 50 bins along the pHluorinSE range (red dots, Supplementary Fig. 9a) and fit a quadratic equation (black line) to model the relationship between pH and GCaMP8m fluorescence. This equation provides an estimate of how GCaMP8m changes are related to pH changes. Data points in Supplementary Fig. 9a that correspond to high pHluorinSE values and low jGCaMP8m values reflect ammonia exposure, which populates the lower-right corner of the plot. As a result, the quadratic fit does not follow the trend of the overshoot observed after CO_2 exposure, seen in the upper-right corner where both pHluorinSE and GCaMP8m values are high.

Using the average pHluorinSE signal during the experiment (Fig. 6f, black line), we applied the fitted equation to compute the pH-dependent GCaMP8m component over time (black line, Supplementary Fig. 9b). We then subtracted this component from the averaged GCaMP8m signal during the experiment (green line) to obtain the calcium signal (blue line). The same correction method was applied to AL and neurons.

2 Supplementary Information

2.1 Supplementary information for sleep behavior observed during imaging

At the start of each trial of probing of the arousal threshold during tethered walking [103], we ensured that the fly was first awake by closing and opening the air stream to the air supported ball once per s for 3 s (see Methods), which stimulated the fly to walk. Then, using an automated control loop for monitoring ball velocity, we detected bouts of immobility. After either 30 s or 5 min of immobility, the power of the heating laser was gradually ramped up until the fly started to walk (see Methods). To ensure that flies were completely still during the detected epochs of immobility, we classified behavior recorded with a camera using machine learning in post-processing (see Methods and Supplementary Fig. S16). Removing those sections where the fly was grooming resulted in the final bouts of immobility between 5 s and 5 min.

2.2 Supplementary information for Hunger and Walking state encoded in dFB neurons

Fig. 5h shows the resulting model time constants, with slow calcium dynamics during the stop state (in the order of min), and feeding related time constants close to zero or the time resolution of our recordings, indicating that dFB activity resets immediately at feeding. Flies stayed satiated (with the hunger component not increasing) on average for around 40 min, although this varied between flies (Fig. 5h). The fitted 'hunger-walk' model (Fig. 5g, Supplementary Figs. S47, and S48) produced a lower error with dFB activity (Fig. 5i) and statistically significant better fits (see Supplementary table S7 and Methods) than a homeostat model (Supplementary Figs. S42, S43). The very fast reset at feeding is not consistent with the behavior of a sleep homeostat.

2.3 Controller glia model

We modeled glia (referring to both EG and AL) as a controller that tries to maintains an optimal pH for neuronal activity, given by H^s, assumed to be $-log(H^s) = 7$. The model includes two compartments: a neuronal or neuropil compartment, where protons are produced at rate r_N, and a glial compartment, where protons enter at a rate d_H and are converted to CO_2 through an equilibrium constant k_{eq} via carbonic anhydrase at rate k. The model schematic is illustrated in Fig. 8a and is governed by the following system of differential equations:

$$\begin{cases} \dot{[H]}_N = r_N - d_H\Big([H]_N - [H]_G\Big) \\ \dot{[H]}_G = d_H\Big([H]_N - [H]_G\Big) + k\Big([CO_2]_G - \Big(k_{eq} + [Ca]\Big)[H]_G\Big) \\ \dot{[CO_2]}_G = -k\Big([CO_2]_G - \Big(k_{eq} + [Ca]\Big)[H]_G\Big) + d_{CO_2}\Big([CO_2]_{(out)} - [CO_2]_G\Big) \\ \dot{[Ca]} = k_i\Big([H]_N - H^s\Big) \end{cases} \tag{22}$$

These differential equations describe Fick's laws of diffusion and are based on the diffusion equation to model diffusion of protons and CO_2 across compartments. Here, $[H]_N$ is the concentration of protons in the neuronal compartment, $[H]_G$ is the concentration of protons in the

glial compartment, and $[CO_2]_G$ is the concentration of CO_2 in the glial compartment. CO_2 is removed from glia at a rate d_{CO_2} through the tracheal system, where the external concentration is assumed to be $[CO_2]_{(out)} = 0$. Calcium in glia $[Ca]$ serves as a controller, modulating the proton-CO_2 equilibrium to maintain the neuronal proton concentration setpoint H^s. The response of the controller to pH deviations is defined by the integral gain constant k_i. Note that we did not include a potential CO_2 source from neuronal metabolic activity in our model. Although a more complex model incorporating CO_2 concentration in neurons is possible, we opted for a simpler approach with fewer differential equations.

The term $k([CO_2]_G - (k_{eq} + [Ca])[H]_G)$ in the second and third differential equation, $\dot{[H]}_G$ and $\dot{[CO_2]}_G$, represents the conversion between CO_2 and protons in the glial compartment. The factor k governs the rate of the conversion reaction. The equilibrium is set by the term $(k_{eq} + [Ca])$, which accounts for the relationship between CO_2 concentration, proton concentration, and calcium-modulated shifts in equilibrium.

To better understand this term, we can isolate the glial compartment and focus solely on the conversion between CO_2 and protons. This results in a simplified model, as shown in equation (23).

$$\begin{cases} \dot{[H]}_G = k\Big([CO_2]_G - \Big(k_{eq} + [Ca]\Big)[H]_G\Big) \\ \dot{[CO_2]}_G = -k\Big([CO_2]_G - \Big(k_{eq} + [Ca]\Big)[H]_G \end{cases} \tag{23}$$

This simplified model represents the reaction kinetics of CO_2 and proton conversion, where the system of differential equations would exponentially evolve towards a stable equilibrium setpoint where $[CO_2]_G = (k_{eq}+[Ca])[H]_G$. Thus, the term $(k_{eq}+[Ca])$ determines the final ratio between $[CO_2]_G$ and proton concentration, with this ratio being modulated by calcium in glia.

The full model described in equation (22) proposes that glia regulate the equilibrium between CO_2 and protons, based on previous findings that AL can influence this balance through pH bicarbonate buffers and ion channels [104, 105]. The model simplifies the various ion channels and buffering mechanisms, offering an abstract representation of glial regulation of pH by adjusting the equilibrium between CO_2 and protons. This conceptualization focuses on glial modulation of the equilibrium without detailing every contributing ion channel and buffer.

2.4 Controller glia model parameters and dynamics

The parameters used in simulations are provided in Table S1, except for r_N. This parameter was determined as a function of the other parameters. Since protons in the neuronal compartment are transferred to the glial compartment and subsequently are converted to CO_2, we defined a baseline proton production rate, r_N, such that the neuronal pH matches the setpoint H^s, ensuring $[H]_N = H^s$. This baseline proton production corresponds to a state of minimal neural activity, such as rest or sleep, where the glia controller does not need to correct pH deviations.

In that case, we can assume that the $[Ca] = 0$, and the model simplifies to the following equations:

H^s (a.u.)	d_H (s^{-1})	k (s^{-1})	k_{eq} (a.u.)	d_{CO_2} (s^{-1})	$[CO_2]_{(out)}$ (a.u.)	k_i (s^{-1})	
1e-7	3.3e-08	0.5	1e7	0.1	10	0	5e4

Table S1: Parameter values for the model simulations in Fig. 8b,e, and f

$$\begin{cases} \dot{[H]}_N = r_N - d_H\Big([H]_N - [H]_G\Big) \\ \dot{[H]}_G = d_H\Big([H]_N - [H]_G\Big) + k\Big([CO_2]_G - k_{eq}[H]_G\Big) \\ \dot{[CO_2]}_G = -k\Big([CO_2]_G - k_{eq}[H]_G\Big) + d_{CO_2}\Big([CO_2]_{(out)} - [CO_2]_G\Big) \end{cases} \tag{24}$$

This simplified model without a controller reaches a stable equilibrium towards a fixed point, given by:

$$\begin{cases} [H]^*_N = \frac{r_N}{k_{eq}}\left(\frac{1}{k} + \frac{1}{d_{CO_2}} + \frac{k_{eq}}{d_H}\right) \\ [H]^*_G = \frac{r_N}{k_{eq}}\left(\frac{1}{k} + \frac{1}{d_{CO_2}}\right) \\ [CO_2]^*_G = r_N/d_{CO_2} \end{cases} \tag{25}$$

From equation (25), we impose $[H]^*_N = H^s$, allowing us to determine the value of r_N required for the pH in the neuronal compartment to reach the setpoint:

$$r_N = \frac{H^s k_{eq}}{\frac{1}{k} + \frac{1}{d_{CO_2}} + \frac{k_{eq}}{d_H}}. \tag{26}$$

Substituting the other parameter values, $r_N \approx 3.3e-8$ s^{-1}. With this value and the parameters listed in Supplementary table S1, the complete model in equation (22) does not require calcium to correct the pH, as the neuronal pH in the compartment is already at the setpoint.

2.5 Controller glia model during wakefulness and sleep

We used the model to simulate calcium activity profiles in EG and EG during sleep and wake cycles (Fig. 8b). During sleep, the proton production rate is set to be r_N , reflecting lower metabolic states, such as those during sleep in the fly, when the glial controller does not need to compensate for any deviations from the pH setpoint. During wakefulness however, higher neural activity leads to CO_2 production and ATP hydrolysis and corresponding acidification [106, 107, 108, 109]. We therefore assume that the proton production rate is increased by 1.5 times during wakefulness (Fig. 8b).

During wakefulness, the pH in the neuronal compartment decreases and protons are transferred into the glial compartment. Calcium senses this deviation from the setpoint and begins to rise. As calcium levels increase, it shifts the equilibrium between CO_2 and protons, as indicated by the term $k_{eq} + [Ca]$ in equation (22). The chosen value of the equilibrium constant k_{eq} reflects the limited conversion of protons to CO_2, which also requires bicarbonate ions.. This

**Simulated 10% CO2** $[CO2]_{(out)}$	**Simulated 50% CO2** $[CO2]_{(out)}$	**Simulated 100% CO2** $[CO2]_{(out)}$
1e-7	5e-7	1e-6

Table S2: Values for $[CO2]_{(out)}$ during the simulated CO_2 exposure.

calcium-driven shift leads to a reduction in proton concentration and an increase in pH within the glial compartment during wakefulness (Fig. 8b, fourth row). The alkalinization of glia during neural activity aligns with previous findings in mammalian astrocytes [110, 111]. An alternative model could result in glial acidification if calcium aimed to maintain the pH setpoint in the glial compartment rather than the neuronal one. The converted CO_2 diffuses through the trachea, with d_{CO_2} chosen to reflect CO_2 diffusion in air. Additionally, the value of the integral gain of the calcium controller, k_i is manually adjusted to align the model dynamics with the observed data.

When calcium levels reach a high threshold, glial cells induce sleep by inhibiting neural activity, thus reducing proton production. In the model, the proton production rate is reset to the value of r_N in Supplementary table S1. This transition from wakefulness to sleep could helps prevent excessive energy expenditure on the glial controller (not modeled here), facilitating the replenishment of energy stores and restoration of pH bicarbonate buffers and other substrates. During sleep, calcium levels gradually decrease while pH returns to the setpoint (Fig. 8b).

2.6 Controller glia model during carbon dioxide exposure

The model discussed in the previous section can explain the calcium slow drift and overshoot during and after CO_2 exposure and optogenetics activation. To simulate CO_2 exposure in the model, we assume that the external CO_2 concentration, $[CO2]_{(out)}$ is nonzero. The values of $[CO2]_{(out)}$ during 10%, 50% and 100% of CO_2 exposure are shown in Supplementary table S2. Fig. 8d shows the response of the model to the three CO_2 concentrations, overlaid with recorded data from pHluorinSE in both neurons and EG, as well as the corrected jGCaMP8 data from EG (see section 1.5 and Extended Data Fig. 9). When $[CO2]_{(out)}$ is present, it enters the glial compartment, converting to protons and causing a decrease in pH within both the glial and neuronal compartments. To account for the observed slow drift of calcium and the overshoot following 50% and 100% CO_2 exposure, we assume that the significant drop in glial pH partially inhibits the calcium controller. Specifically, we assume that the integral gain parameter, k_i is influenced by the pH levels in the glial compartment, given by the relationship shown in Supplementary Fig. S59. This relationship is manually defined using sigmoid functions (equation (27)), one for left pH values lower than 7 (equation (28)), and one for right pH values greater than 7 (equation (29)). These two sigmoids are smoothly stitched together at the center to enable gradual changes in the gain parameter as pH varies (equation (30)).

$$\text{sigmoid}(x, x_c, \sigma) = \frac{1}{1 + e^{\frac{-(x-x_c)}{\sigma}}} \tag{27}$$

$$\text{sigmoid}_{left}(x, x_c, \sigma, y_{min}, y_{max}, x_{max}) = y_{min} + (y_{max} - y_{min}) \frac{\text{sigmoid}(x, x_c, \sigma)}{\text{sigmoid}(x_{max}, x_c, \sigma)} \tag{28}$$

$$\text{sigmoid}_{right}(x, x_c, \sigma, y_{min}, y_{max}, x_{min}) = y_{min} + (y_{max} - y_{min}) \frac{1 - \text{sigmoid}(x, x_c, \sigma)}{1 - \text{sigmoid}(x_{min}, x_c, \sigma)} \tag{29}$$

$$K_i(x) = \begin{cases} \text{sigmoid}_{left}(x, x_{c1}, \sigma_1, y_{min}, y_{max}, x_{min}) & \text{if} \quad x \leq x_{min} \\ y_{max} \quad \text{if} \quad x_{min} < x < x_{max} \\ \text{sigmoid}_{right}(x, x_{c2}, \sigma_2, y_{min}, y_{max}, x_{max}) & \text{if} \quad x \geq x_{min} \end{cases} \tag{30}$$

As pH decreases, k_i in the model decreases as well (Fig. 8c), indicating a reduction in the effectiveness of the controller under acidic conditions. This partial inhibition leads to a slow response to pH deviations, resulting in a gradual rise in calcium and a modest increase in pH during 50% and 100% CO_2 exposure (Fig. 8d).

At the end of 50% and 100% CO_2 exposure, respectively, the external CO_2 concentration, $[CO2]_{(out)}$ is set to zero, leading to a decrease in proton concentration in the glial compartment. As the pH returns to physiological levels, the partial inhibition of the calcium controller is lifted, restoring k_i to its normal value. For a brief period, the pH in the neuronal compartment is still low (since the protons have not been removed yet), causing the calcium controller to detect a significant deviation from the setpoint and respond strongly. This generates the observed overshoot dynamics after CO_2 exposure (Fig. 8d), similarly to an impulse response in control systems.

The calcium overshoot leads to an increase in pH in both the neuronal and glial compartments. This elevated alkaline pH inhibits the controller, as indicated by the brief decrease in k_i at the end of CO_2 exposure (shown in Fig. 8d). Consequently, this inhibition results in a delayed response in calcium dynamics, as seen in the different overshoot patterns observed after 50% and 100% CO_2 exposure. Once the glial pH returns to baseline, calcium levels also normalize. This model therefor explains the drift and overshoot dynamics during CO_2 exposure by assuming that the glia controller is pH-dependent and partially inhibited outside its physiological range. (For the biological rationale behind controller inhibition during low pH, see section 2.8.)

2.7 Controller glia model during optogenetics activation

Similar dynamics are observed during optogenetic activation and CO_2 exposure experiments. Since we express CSChrimson in glia, which acts as a proton channel[112], we simulate the effects of optogenetic activation by adding an additional term, r_G, into the glial compartment:

$$\dot{[H]}_G = d_H\Big([H]_N - [H]_G\Big) + k\Big([CO_2]_G - \Big(k_{eq} + [Ca]\Big)[H]_G\Big) + r_G \tag{31}$$

The term r_G represents the rate at which protons enter the glial compartment during optogenetic activation, and it is set at $r_G = 90r_N$ during this activation period. Because glial cells are negatively charged, the opening of proton channels allows protons to flow into the glial

compartment through the electric gradient, resulting in acidification of this compartment (Fig. 8e).

The model simulation during optogenetic activation for 120 s is displayed in Fig. 8f, overlaid with the recorded calcium patterns from EG in FB. The observed slow drift and overshoot during and after optogenetic activation can be explained by the partial inhibition of the controller due to acidification, similar to the mechanisms described in the previous section regarding CO_2 exposure.

2.8 Potential mechanisms for biological implementation of the proposed model

The proposed model is consistent with carbonic anhydrase observed specifically in glia (Fig. 6d), which can also contribute to calcium regulation in some species [113], and the close connection between trachea and glia facilitating gas exchange (Fig. 6). Further, neural activity leads to CO_2 production and ATP hydrolysis and corresponding acidification [106, 107, 108, 109, 114]. In the proposed model, alkalinization of glia during neural activity (Fig. 8b, fourth row), is consistent with previous data in mammalian astrocytes [110, 111]. However, a similar model with acidification in both compartments could similarly explain the data. The model is further consistent with homeostatic chemosensory function of glia described in mammals [115, 116, 117, 118, 119, 120]. Additionally, glia are known to regulate intracellular and extracellular pH through membrane-bound transporters and ion channels, activated by calcium signaling [104, 111]. Controller inhibition could for example result from inhibition of pH-regulatory ion channels under acidic conditions [107].

References

[95] Jonas Bittern et al. "Neuron–glia interaction in the Drosophila nervous system". In: *Developmental neurobiology* 81.5 (2021), pp. 438–452.

[96] Sophie Aimon et al. "Global change in brain state during spontaneous and forced walk in Drosophila is composed of combined activity patterns of different neuron classes". In: *eLife* 12 (2023), e85202.

[97] Florian G Richter et al. "Glutamate signaling in the fly visual system". In: *Iscience* 7 (2018), pp. 85–95.

[98] Jonathan S Marvin et al. "An optimized fluorescent probe for visualizing glutamate neurotransmission". In: *Nature methods* 10.2 (2013), pp. 162–170.

[99] James P Bohnslav et al. "DeepEthogram, a machine learning pipeline for supervised behavior classification from raw pixels". In: *Elife* 10 (2021), e63377.

[100] John E Zimmerman et al. "A video method to study Drosophila sleep". In: *Sleep* 31.11 (2008), pp. 1587–1598.

[101] Joan C Hendricks et al. "Rest in Drosophila is a sleep-like state". In: *Neuron* 25.1 (2000), pp. 129–138.

[102] Bart Van Alphen et al. “A deep sleep stage in Drosophila with a functional role in waste clearance”. In: *Science advances* 7.4 (2021), eabc2999.

[103] Melvyn HW Yap et al. “Oscillatory brain activity in spontaneous and induced sleep stages in flies”. In: *Nature communications* 8.1 (2017), pp. 1–15.

[104] Shefeeq M Theparambil, Gulnaz Begum, and Christine R Rose. “pH regulating mechanisms of astrocytes: A critical component in physiology and disease of the brain”. In: *Cell Calcium* 120 (2024), p. 102882.

[105] Shefeeq M Theparambil et al. “Astrocytes regulate brain extracellular pH via a neuronal activity-dependent bicarbonate shuttle”. In: *Nature Communications* 11.1 (2020), p. 5073.

[106] M Chesler and K Kaila. “Modulation of pH by neuronal activity”. In: *Trends in neurosciences* 15.10 (1992), pp. 396–402.

[107] Mitchell Chesler. “Regulation and modulation of pH in the brain”. In: *Physiological reviews* 83.4 (2003), pp. 1183–1221.

[108] Sachin Makani and Mitchell Chesler. “Rapid rise of extracellular pH evoked by neural activity is generated by the plasma membrane calcium ATPase”. In: *Journal of neurophysiology* 103.2 (2010), pp. 667–676.

[109] Rune Rasmussen et al. “Interstitial ions: A key regulator of state-dependent neural activity?” In: *Progress in neurobiology* 193 (2020), p. 101802.

[110] Mitchell Chesler and Richard P Kraig. “Intracellular pH transients of mammalian astrocytes”. In: *Journal of Neuroscience* 9.6 (1989), pp. 2011–2019.

[111] Alexei Verkhratsky, Verena Untiet, and Christine R Rose. “Ionic signalling in astroglia beyond calcium”. In: *The Journal of physiology* 598.9 (2020), pp. 1655–1670.

[112] Johannes Vierock et al. “Molecular determinants of proton selectivity and gating in the red-light activated channelrhodopsin Chrimson”. In: *Scientific Reports* 7.1 (2017), p. 9928.

[113] Ashok Aspatwar et al. “Carbonic anhydrases in metazoan model organisms: molecules, mechanisms, and physiology”. In: *Physiological reviews* 102.3 (2022), pp. 1327–1383.

[114] Joachim W Deitmer. “A role for CO2 and bicarbonate transporters in metabolic exchanges in the brain”. In: *Journal of neurochemistry* 80.5 (2002), pp. 721–726.

[115] Alexander V Gourine et al. “Astrocytes control breathing through pH-dependent release of ATP”. In: *Science* 329.5991 (2010), pp. 571–575.

[116] Plamena R Angelova et al. “Functional oxygen sensitivity of astrocytes”. In: *Journal of Neuroscience* 35.29 (2015), pp. 10460–10473.

[117] Vitaliy Kasymov et al. “Differential sensitivity of brainstem versus cortical astrocytes to changes in pH reveals functional regional specialization of astroglia”. In: *Journal of Neuroscience* 33.2 (2013), pp. 435–441.

[118] Egor Turovsky et al. “Mechanisms of CO2/H+ sensitivity of astrocytes”. In: *Journal of Neuroscience* 36.42 (2016), pp. 10750–10758.

Fly	Sleep using ball velocity (stop) (% per h)	Sleep using walk density (rest) (% per h)	Sleep with 5 min threshold (% per h)
1	50.0	49.1	18.7
2	56.8	51.5	28.8
3	68.1	61.0	33.6
4	70.8	51.6	30.4
5	57.6	46.0	19.1
6	76.4	61.2	31.0

Table S3: Percentage of time sleeping per h for each fly according to the different definitions used in the paper.

Fly	Number of points	Pearson's correlation (EB)	Pearson's correlation (FB)	p-value (EB)	p-value (FB)
**1**	1664	0.46	0.37	5.3e-86	3.8e-56
**2**	1543	0.31	0.25	1.7e-35	4.5e-23
**3**	680	0.12	0.16	1.2e-03	3.8e-04
**4**	1065	0.61	0.44	2.1e-108	3.6e-51
**5**	1283	0.37	0.25	2.3e-43	1.0e-19
**6**	904	0.28	0.28	1.2e-17	2.9e-18

Table S4: Pearson's correlation between the changes in high frequency fluorescence fluctuations (change in $\Delta F/F$) and changes in ball velocity.

[119] Alexei Verkhratsky and Maiken Nedergaard. "Physiology of astroglia". In: *Physiological reviews* 98.1 (2018), pp. 239–389.

[120] Ciaran Murphy-Royal, ShiNung Ching, and Thomas Papouin. "A conceptual framework for astrocyte function". In: *Nature neuroscience* 26.11 (2023), pp. 1848–1856.

Fly	L2 error in 2-state model (EB)	L2 error in 7-state model (EB)	F-statistic (EB)	p-value (EB)
1	3.479988	3.478012	0.32	0.9
2	3.063297	2.696925	70.74	1.1e-16
3	4.955734	4.186857	56.23	1.1e-16
4	4.281183	4.167001	13.01	1.6e-12
5	1.520076	1.658245	-40.54	1.0
6	11.306768	10.176129	61.61	1.1e-16

Table S5: Comparison between 2-state and 7-state models in EB

Fly	L2 error in 2-state model (FB)	L2 error in 7-state model (FB)	F-statistic (FB)	p-value (FB)
1	3.588580	3.246188	59.59	1.1e-16
2	0.784993	0.733569	36.50	1.1e-16
3	3.402890	2.818227	63.52	1.1e-16
4	1.604965	1.512774	28.95	1.1e-16
5	2.616210	2.554074	11.84	1.0
6	5.747171	5.153419	63.90	1.1e-16

Table S6: Comparison between the 2-state and 7-state models in FB

Fly	L2 error in homeostat model	L2 error in hunger-walk model	F-statistic	p-value
1	0.093624	0.041422	1395.11	1.110223e-16
2	0.131172	0.055818	790.07	1.110223e-16
3	0.084375	0.057946	233.75	1.110223e-16
4	0.075678	0.058143	90.18	1.110223e-16
5	0.101618	0.045378	635.48	1.110223e-16
6	0.065754	0.039686	84.41	1.110223e-16
7	0.066197	0.037298	498.03	1.110223e-16
8	0.101609	0.043907	1574.07	1.110223e-16

Table S7: Comparison between the homeostat model and hunger-walk model for dFB neurons

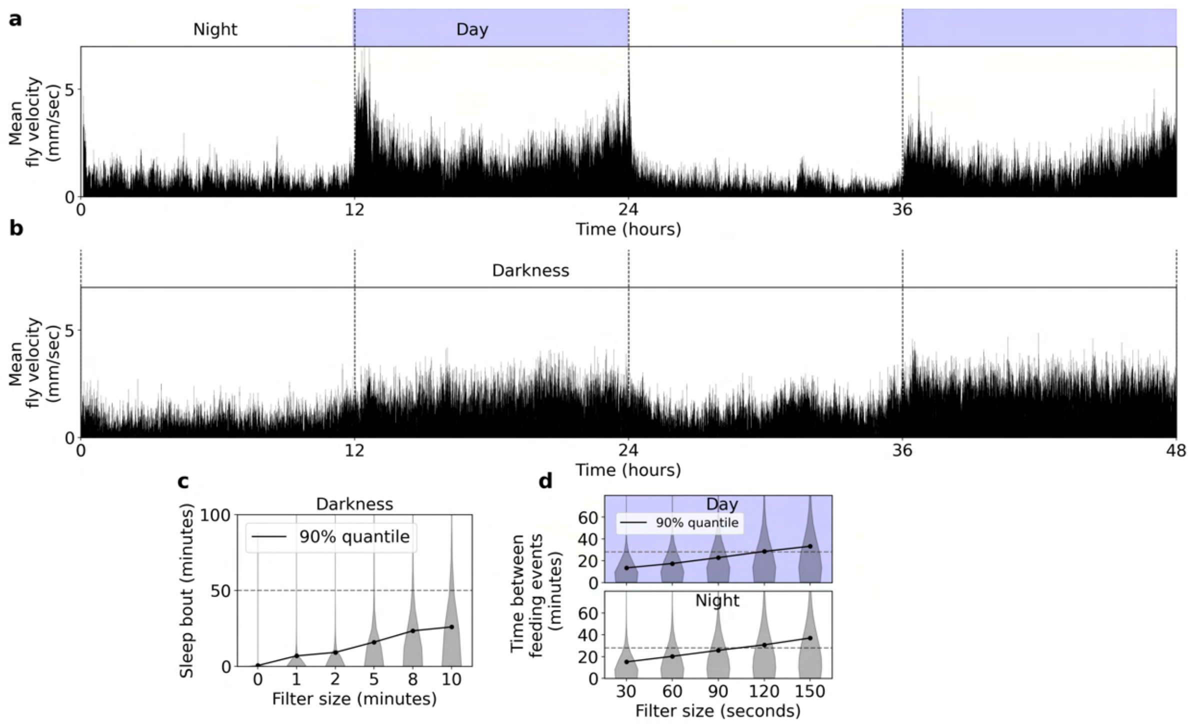

Supplementary Figure S1: Behavioral activity of freely moving flies. **a** Mean fly velocity in 1 s bins over 48 h with 12 h of light (day) and 12 h of darkness (night). **b** Same as in a, but in constant darkness. **c** Distribution of sleep bouts for a total of 15 flies in complete darkness (from b) as function of filter size. **d** Distribution of time between feeding events in freely moving flies as a function of filter size (see Methods).

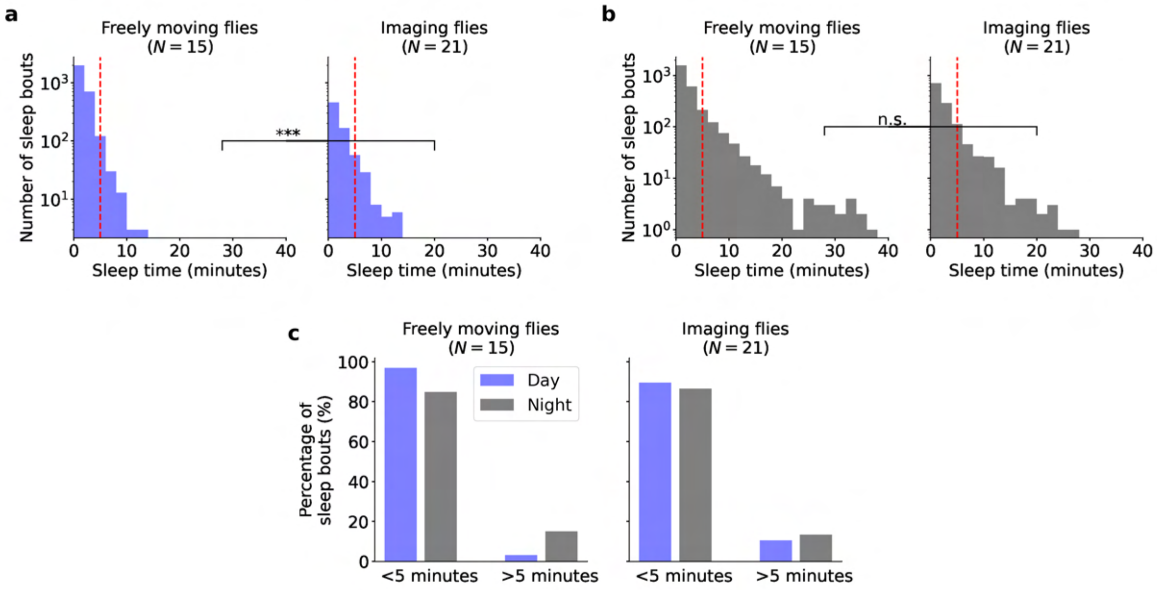

Supplementary Figure S2: Comparison of sleep distribution in freely walking flies (recorded as in Fig. 1a) and flies during long-term imaging experiments. **a** Distribution of sleep bouts in freely moving flies (left) and flies during imaging experiments (right) during the day. The three asterisk indicates statistical difference between the two distributions using the Kolmogorow-Smirnow test ($p < 0.00005$). **b** Same as a but during the night. No statistical significance is found between these two distributions (see statistics in Supplementary Data S1). **c** Left panel: percentage of sleep bouts shorter (left) and larger (right) than 5 min during the day (blue) and night (gray) in freely moving flies. Right panel: same as left panel, but for flies during long-term imaging experiments.

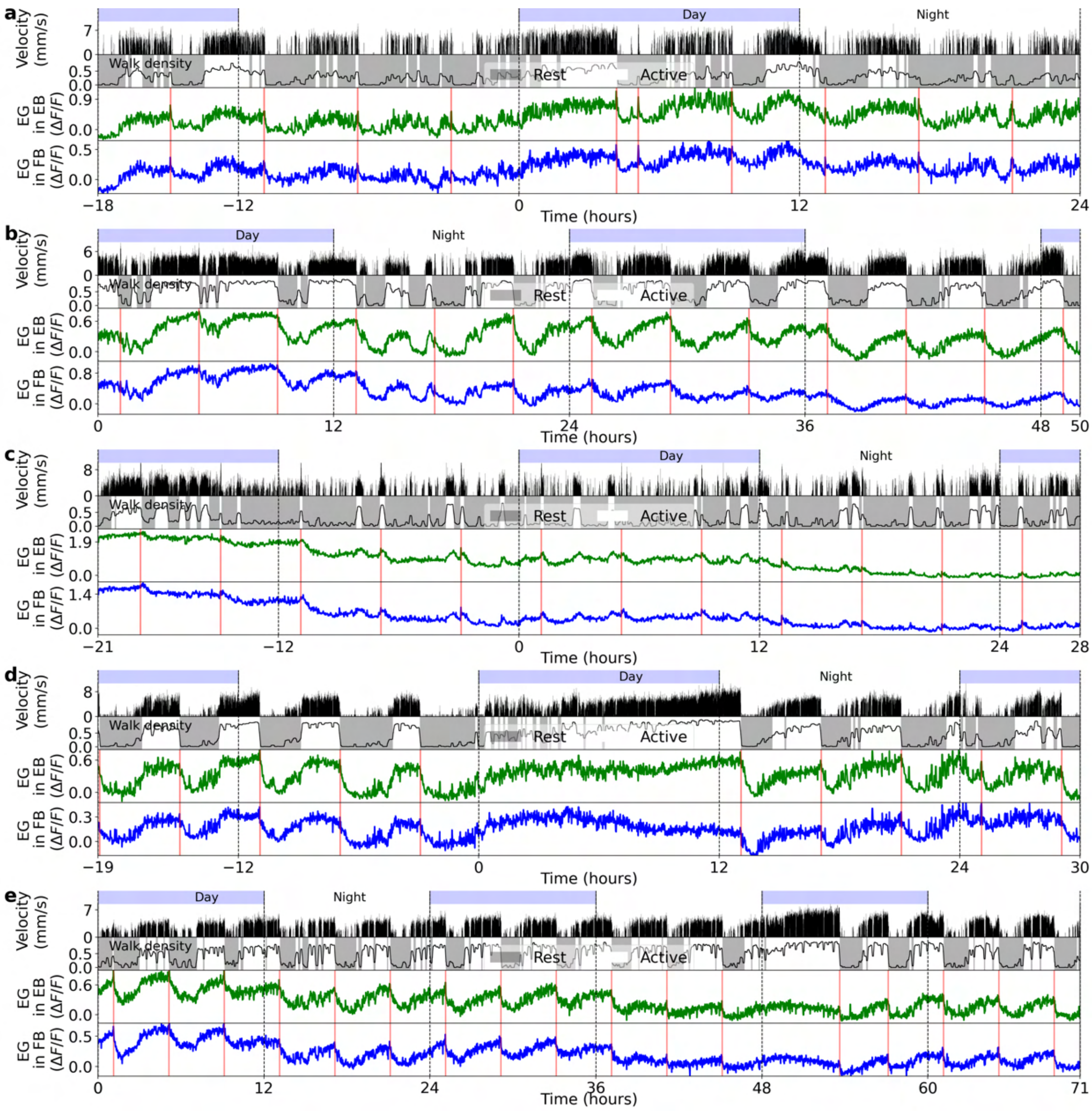

Supplementary Figure S3: Five different recordings in EG where flies are fed every 4 h. **a** Top row: day and night cycle in VR. Second row: velocity of the fly in 1 s bins. Third row: walk density (see Methods) and predominantly rest (gray region) and predominantly active (white region) epochs (see Methods). Fourth and fifth row: Calcium activity of EG in EB (green) and FB (blue), respectively. Thick lines indicate low-pass filter with a 0.1 h cut-off period, while vertical red lines represent feeding events. **b-e** Same as a. Each panel shows different flies.

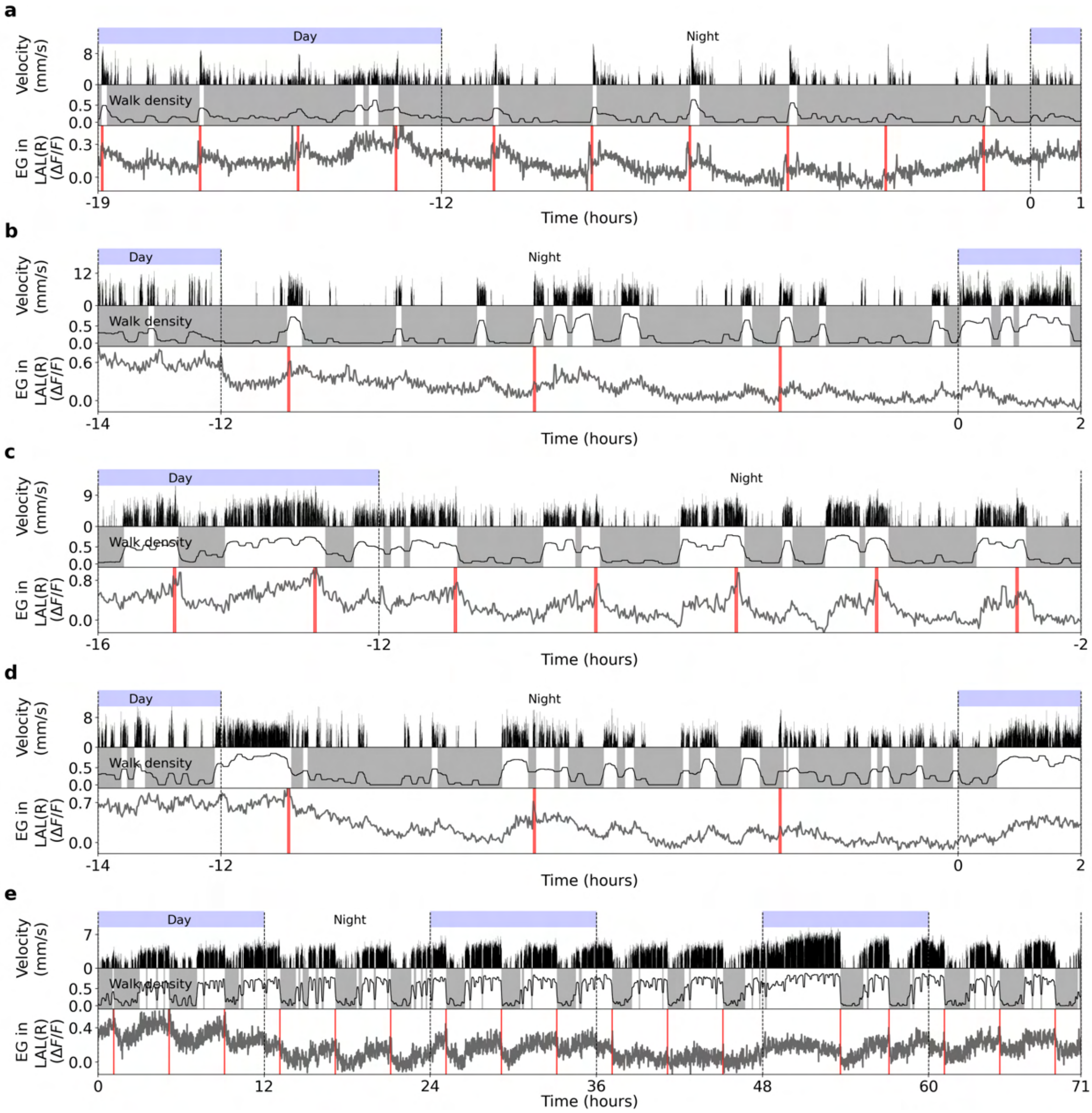

Supplementary Figure S4: EG calcium activity in the LAL for 5 flies fed every 4 h. **a** Top row: day and night cycle in VR. Second row: velocity of the fly in 1 s bins. Third row: walk density (see Methods) and predominantly rest (gray region) and predominantly active (white region) epochs. Fourth row: Calcium activity of EG in the LAL (gray). Thick lines indicate a low-pass filter with 0.1 h cut-off period, while vertical red lines represent feeding events. **b-e** Same as a. Each panel shows a different fly.

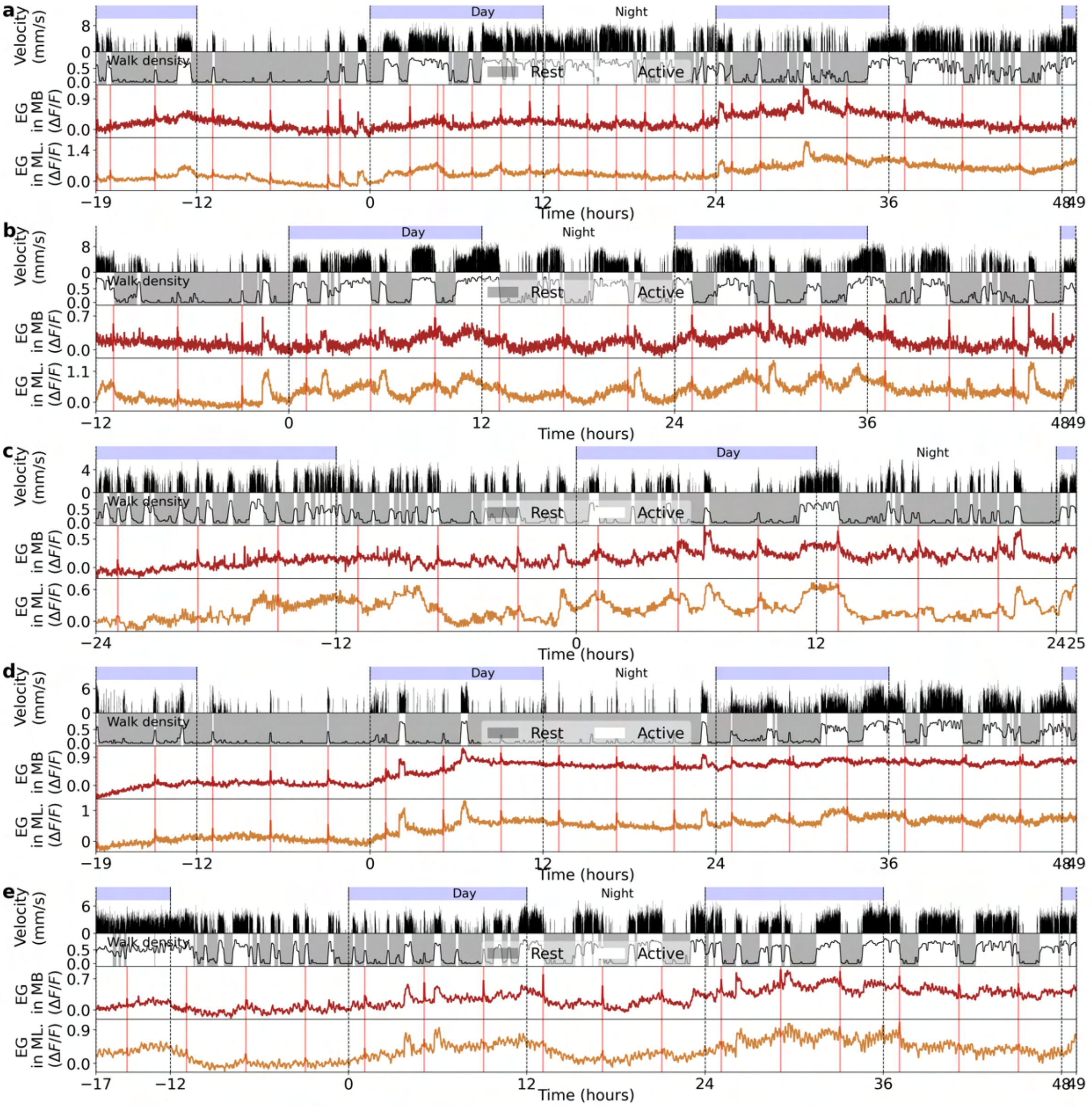

Supplementary Figure S5: EG calcium activity in MB and midline for 5 flies fed every 4 h. **a** Top row: day and night cycle in VR. Second row: velocity of the fly in 1 s bins. Third row: walk density (see Methods) and predominantly rest (gray region) and predominantly active (white region) epochs. Fourth and fifth row: Calcium activity of EG in MB (brown) and midline (dark orange). Thick lines indicate a low-pass filter with 0.1 h cut-off period, while vertical red lines represent feeding events. **b-e** Same as a. Each panel shows a different fly.

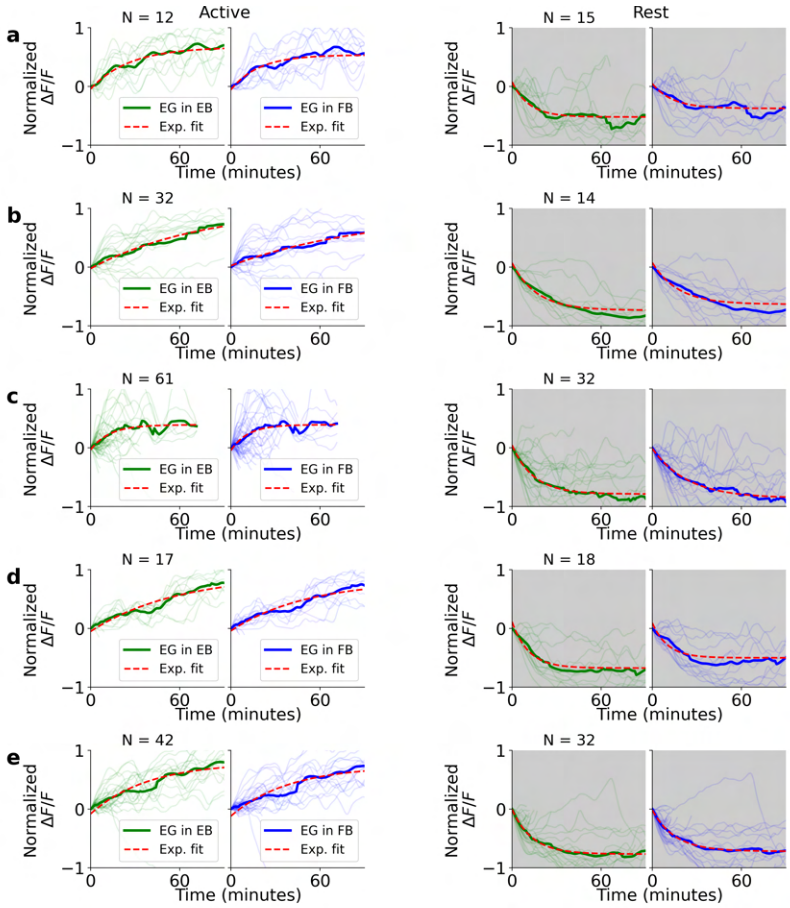

Supplementary Figure S6: Normalized fluorescence traces during active and rest epochs for 5 flies. **a** Left side: single (thin lines) and average (thick lines) normalized fluorescence traces in EB (green) and FB (blue) during active epochs. Red lines indicate exponential fit. Right side (gray background): same as left side, but during rest epochs. **b-e** Same as a. Each panel is from a different fly.

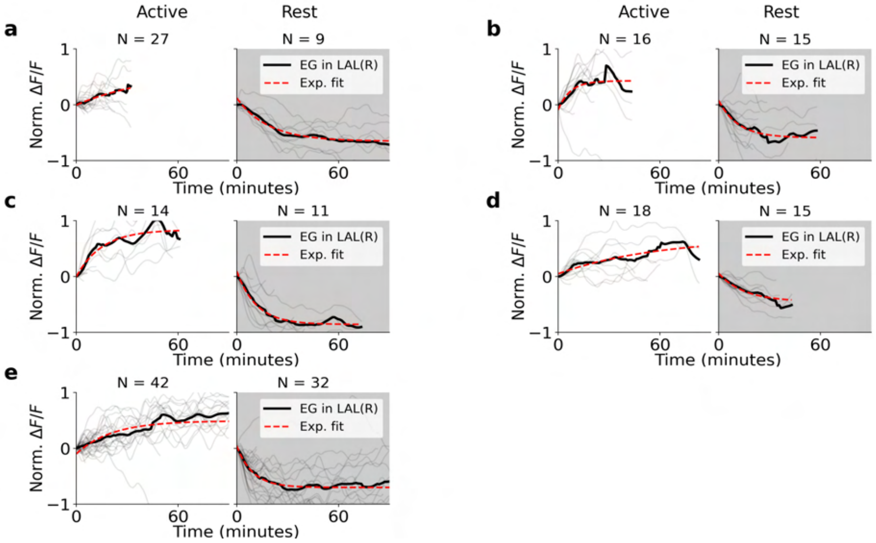

Supplementary Figure S7: Normalized fluorescence traces during active and rest epochs for 5 flies. **a** Left side: single (thin lines) and average (thick lines) normalized fluorescence traces in the LAL (gray) during active epochs. Red lines indicate exponential fit. Right side (gray background): same as left side, but during rest epochs. **b-e** Same as a. Each panel is from a different fly.

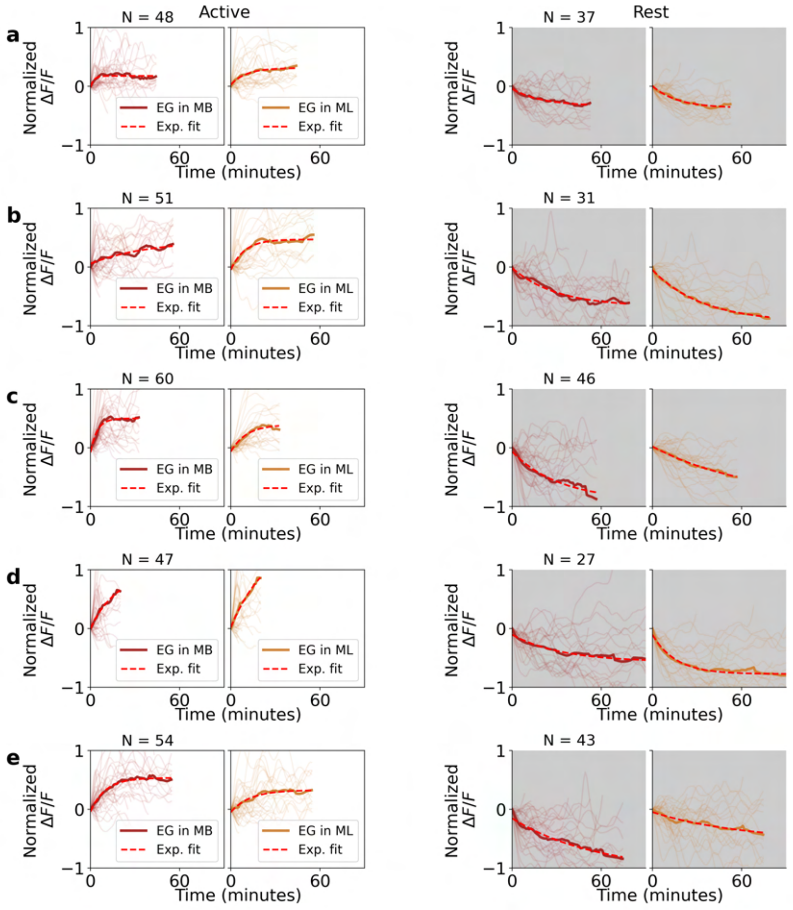

Supplementary Figure S8: Normalized fluorescence traces during active and rest epochs for 5 flies. **a** Left side: single (thin lines) and average (thick lines) normalized fluorescence traces in MB (brown) and midline (dark orange) during active epochs. Red lines indicate exponential fit. Right side (gray background): same as left side, but during rest epochs. **b-e** Same as a. Each panel is from a different fly.

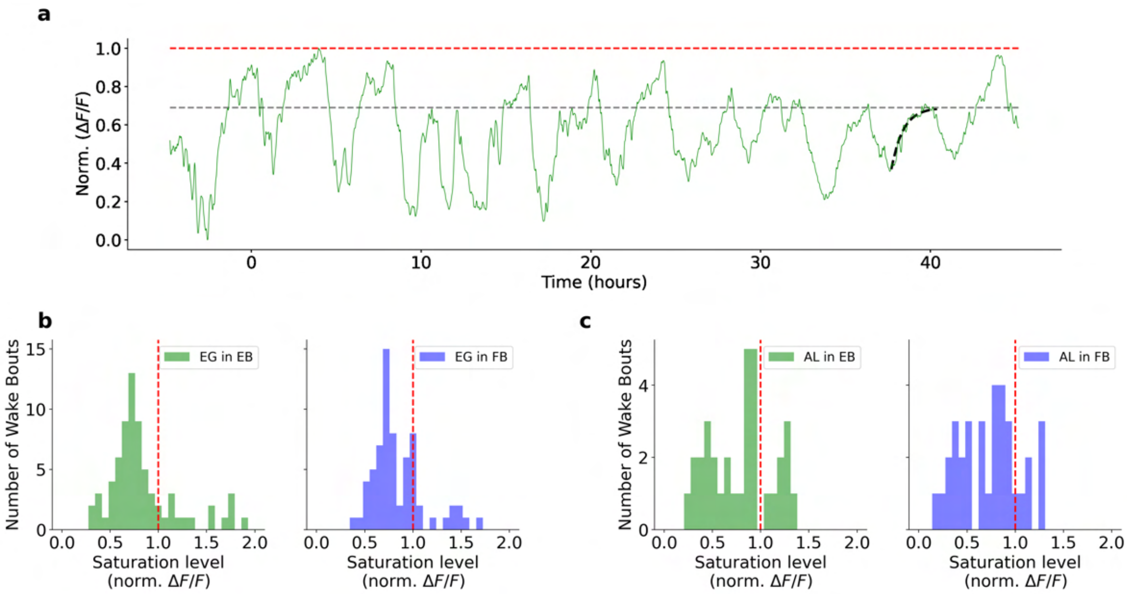

Supplementary Figure S9: Fluorescence saturates due to calcium dynamics and not due to sensor saturation. **a** Filtered and normalized calcium activity recorded in EB from EG (green) over a long-term imaging experiment. This example shows an exponential fit (black) when the fly was active, the saturation level (gray) of the exponential, and the maximum recorded signal, normalized at 1 (red). The saturation level was lower than the maximum value of fluorescence. **a** Distribution of saturation levels (from fitted exponentials) during active bouts in EB (left) and FB (right) for EG. The distributions reveal that the majority of saturation levels are below 1 (shown as the red vertical line as the normalized maximum fluorescence, indicating that calcium activity reaches saturation in most active periods before the sensor reaches its maximum during the experiment (for 6 flies shown in Fig. 1i and Supplementary Fig. S3). **c** Same as b but for AL (for 5 flies shown in Fig. 2a and Supplementary Fig. S12). Alternatively, this can be inferred from unfiltered raw data, where saturation with walking bouts is modulated additionally with circadian fluctuations (Fig 1i, 2a, and Supplementary Figs. S3, S12).

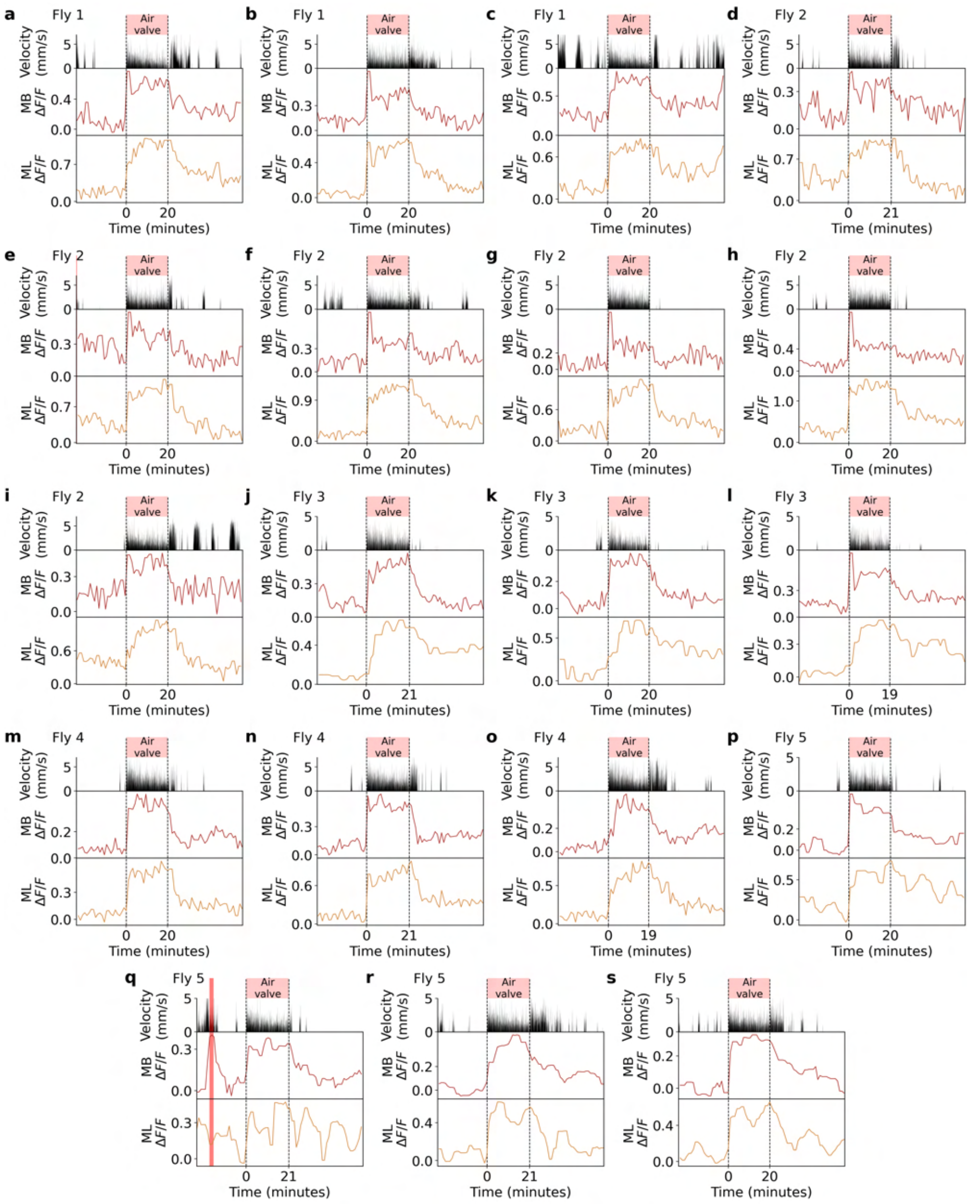

Supplementary Figure S10: Trials where the air stream supporting the ball was intermittently switched on and off every second to promote walking behavior, while glia calcium activity in MB and midline was recorded. **a** First row: time where the air stream to the ball was intermittently interrupted to promote walking activity (red region). Second row: velocity of the fly. Third and fourth rows: calcium activity of EG in MB (brown) and midline (dark orange). **b-s** Same as a. Vertical red line in q indicates feeding event. Each panel represents a different trial from a total of 5 flies. The fly from which each trial was obtained is shown in the top left corner of each panel.

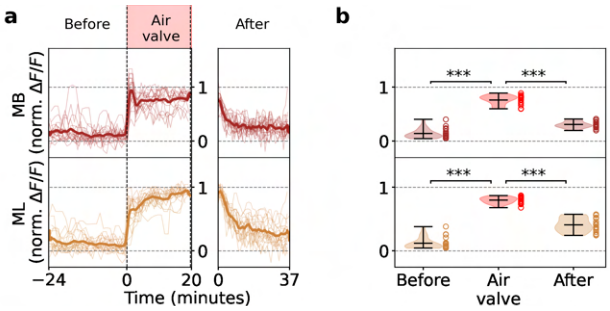

Supplementary Figure S11: Glia activity in MB and midline increases with walking behavior. **a** Normalized fluorescence traces of MB (brown) and midline (dark orange) of all trials ($n = 19$, from 5 flies, represented as thin lines, see Supplementary Fig. S10) before, during, and after the air valve was used to promote walking. Thick lines indicate average normalized fluorescence of all trials. **b** Distribution of mean fluorescence levels from panel a of all trials before, during, and after the air valve perturbation. Three asterisks indicate statistical significance using two-sided t-test ($p < 0.0005$, for detailed statistics information, see Supplementary Data S1).

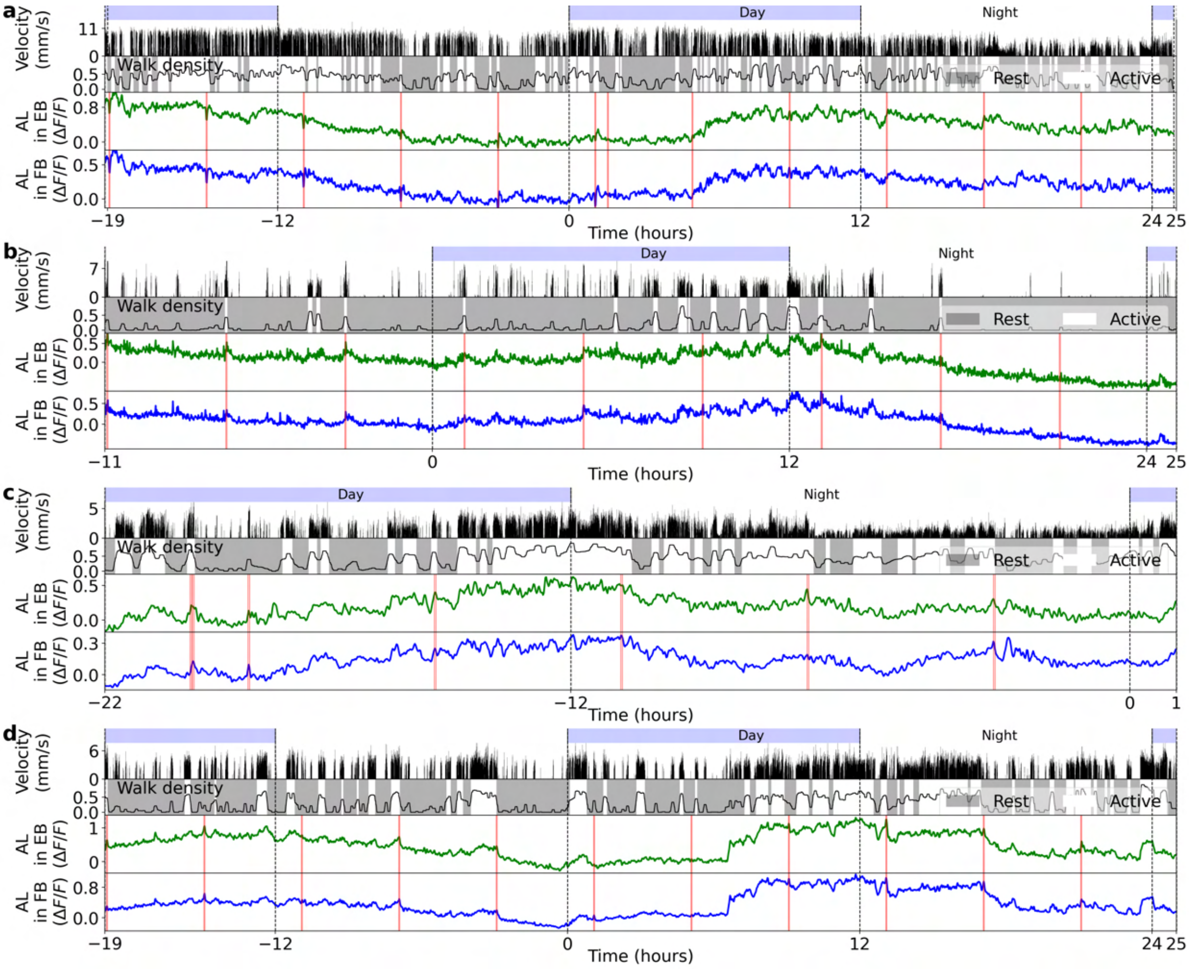

Supplementary Figure S12: AL calcium activity in EB and FB for 4 flies fed every 4 h. **a** Top row: day and night cycle in VR. Second row: velocity of the fly in 1 s bins. Third row: walk density (see Methods) and rest (gray region) and active (white region) epochs. Fourth and fifth row: calcium activity of EG in EB (green) and FB (blue). Vertical red lines represent feeding events. **b-d** Same as a. Each panel shows a different fly. See also Fig. 2a.

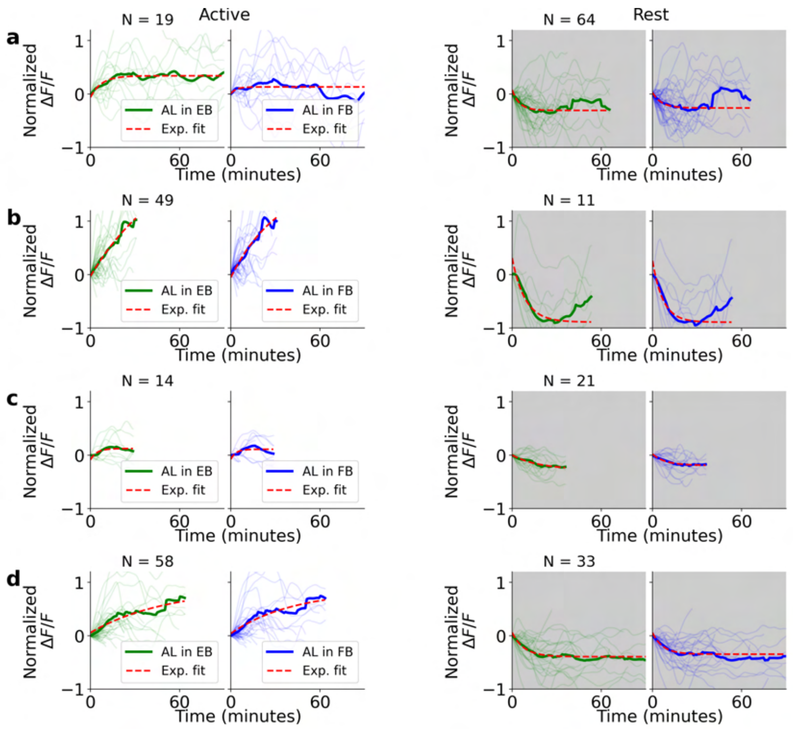

Supplementary Figure S13: Normalized fluorescence traces in AL during active and rest epochs for 4 flies. **a** Left side: single (thin lines) and average (thick lines) normalized fluorescence traces in EB (green) and FB (blue) during active epochs. Red lines indicate exponential fit. Right side (gray background): same as left side, but during rest epochs. **b-d** Same as a. Each panel is a different fly. See also Fig. 2b.

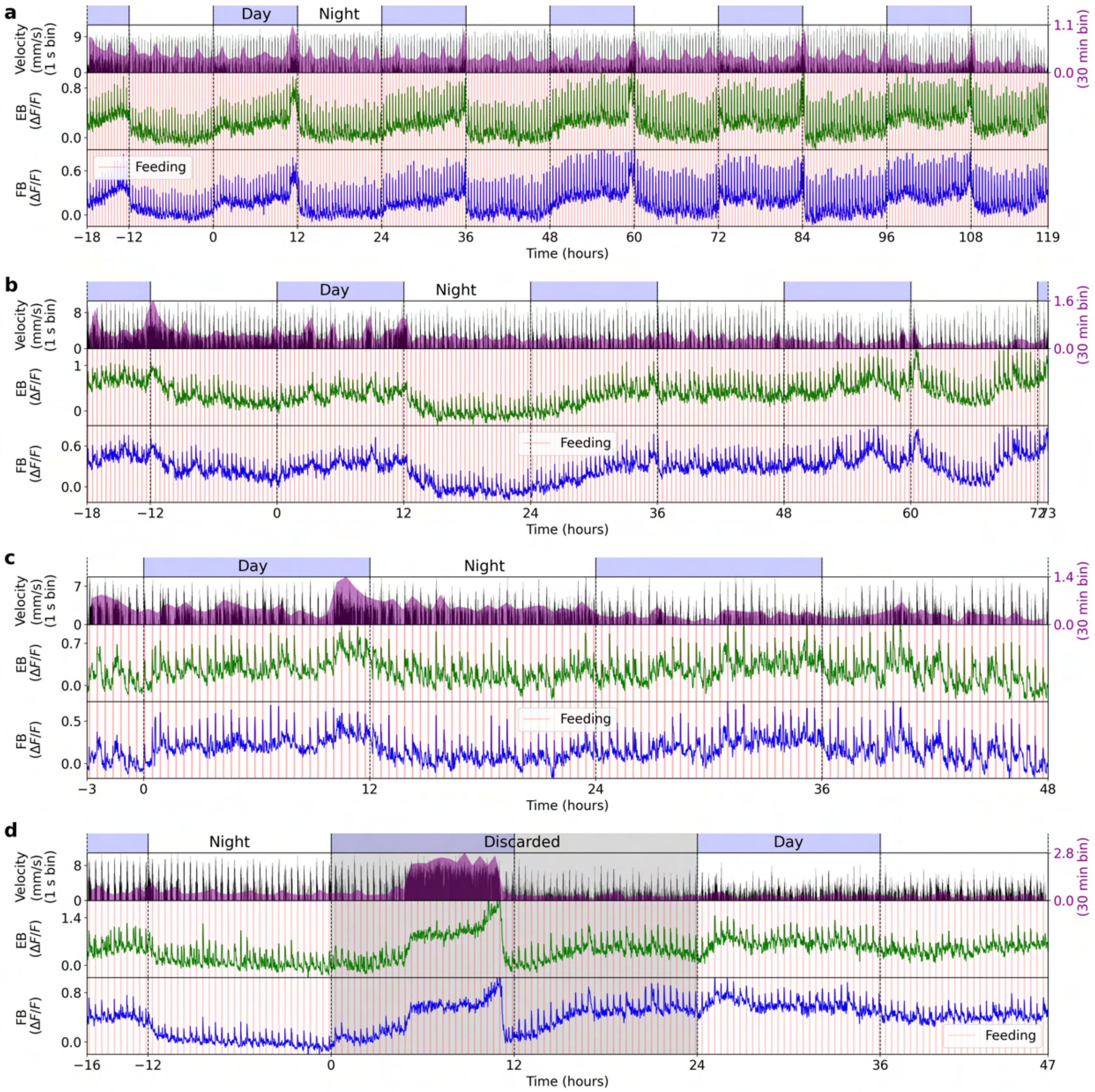

Supplementary Figure S14: Four different recordings in EG where flies are fed every 26 min. **a** Top row: day and night cycle in VR. Second row: in black, velocity of the fly in 1 s bins. black: velocity of the fly in 30 min bins. Third and fourth row: calcium activity of EG in EB (green) and FB (blue). Vertical red lines indicate feeding events. **b-d** Same as a. Each panel shows a different fly. In panel d, the gray area was discarded from the average in Fig. 5j, since there was an epoch of increased walking and calcium activity between h 5 and 11. The reason for this increase during the recording is unknown but was likely due to an unexpected event during the recording such as a sudden increase in two-photon laser power or temperature. We also discarded the subsequent 12 h of the experiment. See also Supplementary Fig. S15 .

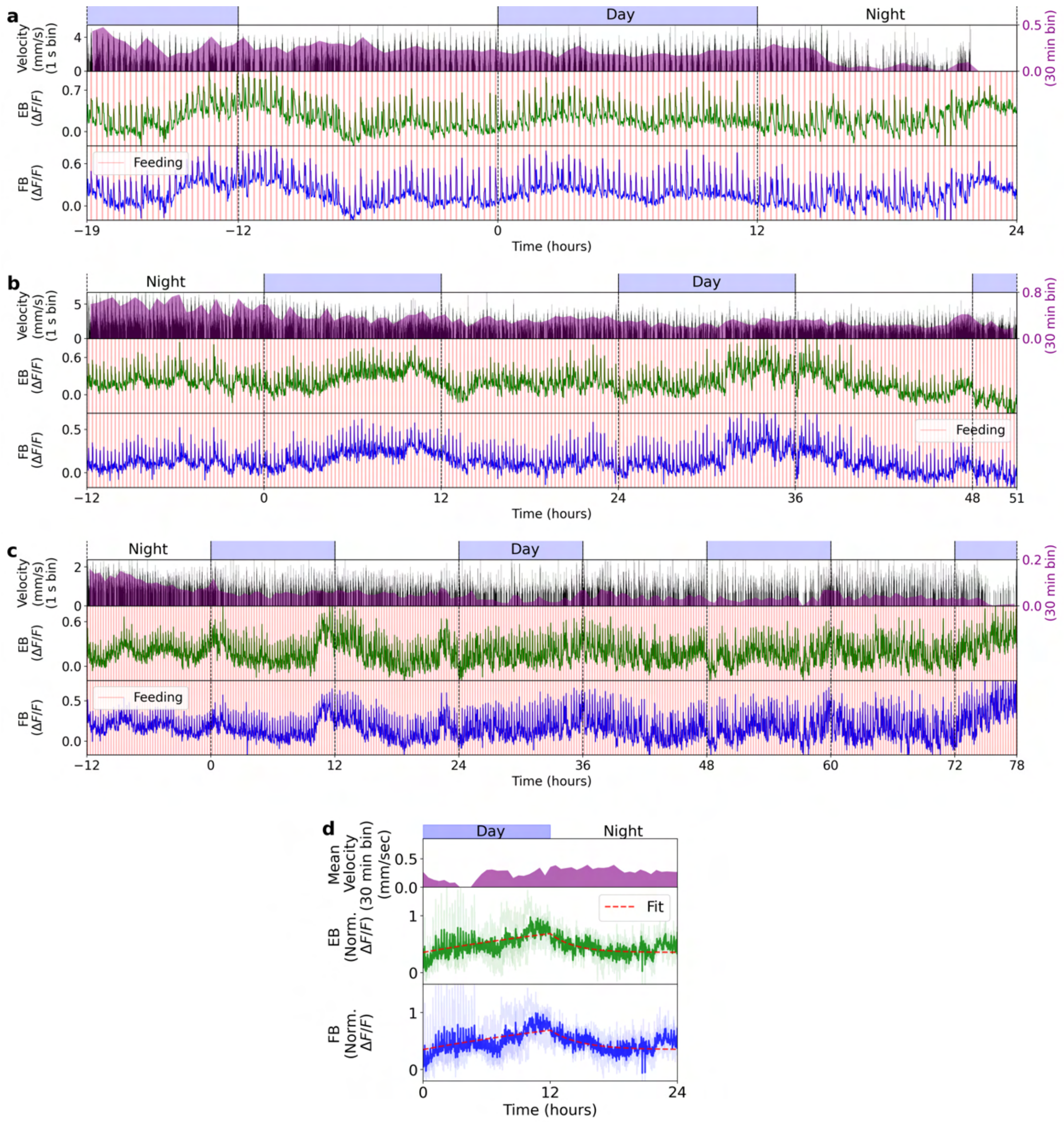

Supplementary Figure S15: Three different long-term imaging recordings in EG where flies are fed every 16 min. **a** Top row: day and night cycle in VR. Second row: in black, velocity of the fly in 1 s bins. black: velocity of the fly in 30 min bins. Third and fourth row: Calcium activity of EG in EB (green) and FB (blue), respectively. Vertical red lines indicate feeding events. **b, c** Same as a, but for different flies. **d** Average of previous recordings (in a-c) with all trials aligned to one 24 h period. Top row: day and night cycle. Second row: Mean velocity of all flies over 30 min bins. Third and fourth row: average glia activity in EB (green) and FB (blue). Red lines indicate exponential fits during the day and night (see Methods). See also Supplementary Fig. S14.

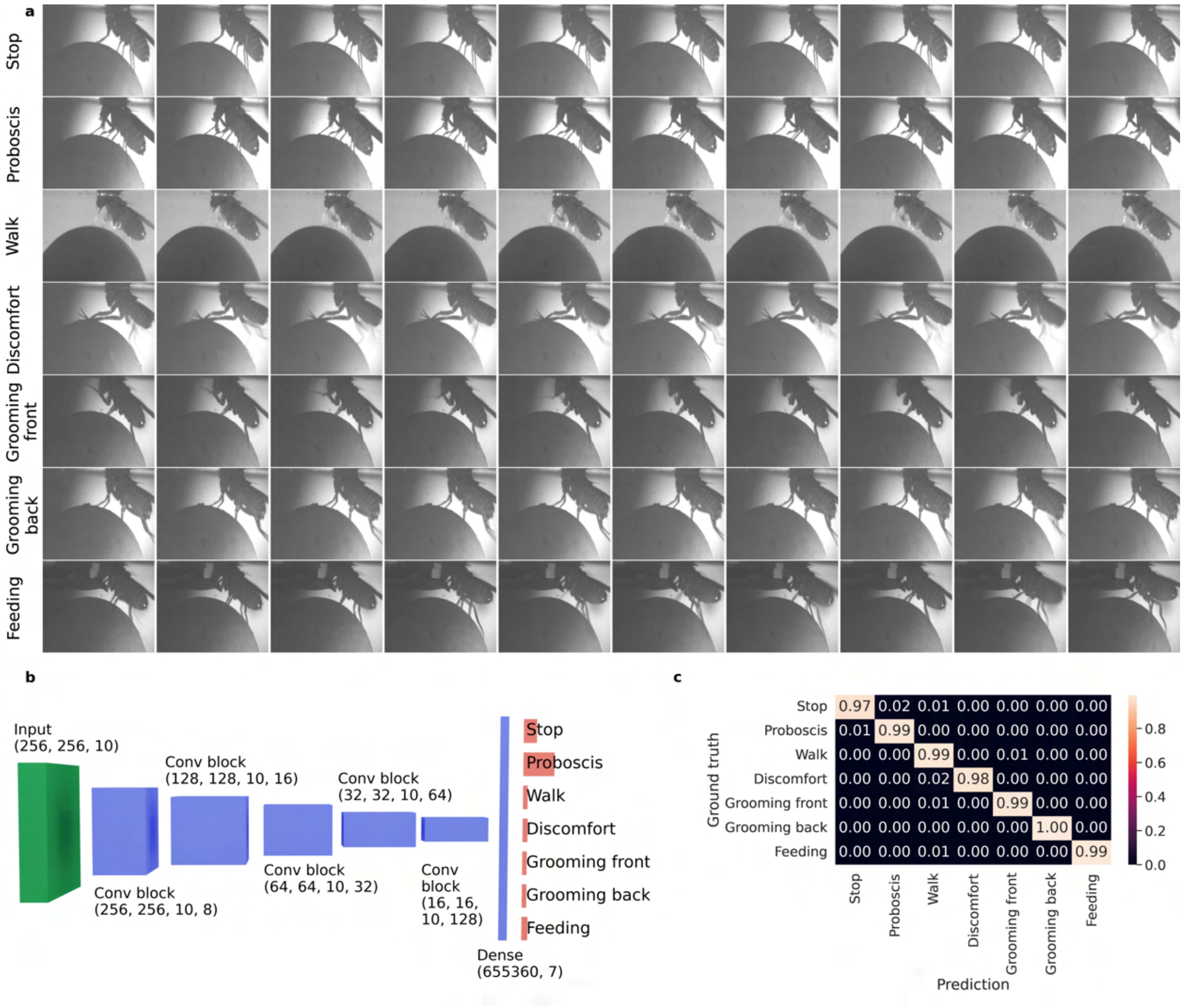

Supplementary Figure S16: Classification of fly behavior on the ball during long-term imaging. **a** A 3D CNN with 10 consecutive frames with a side view of the fly on the ball is used to classify 7 different behaviors recorded at 10 Hz (y-axis). Each row in the plot corresponds to 10 consecutive frames where the fly performs the labeled behavior. **b** Architecture of the 3D CNN. **c** Confusion matrix for each behavior for prediction of the 3D CNN (x-axis) on a test dataset that was manually labeled (ground truth along y-axis).

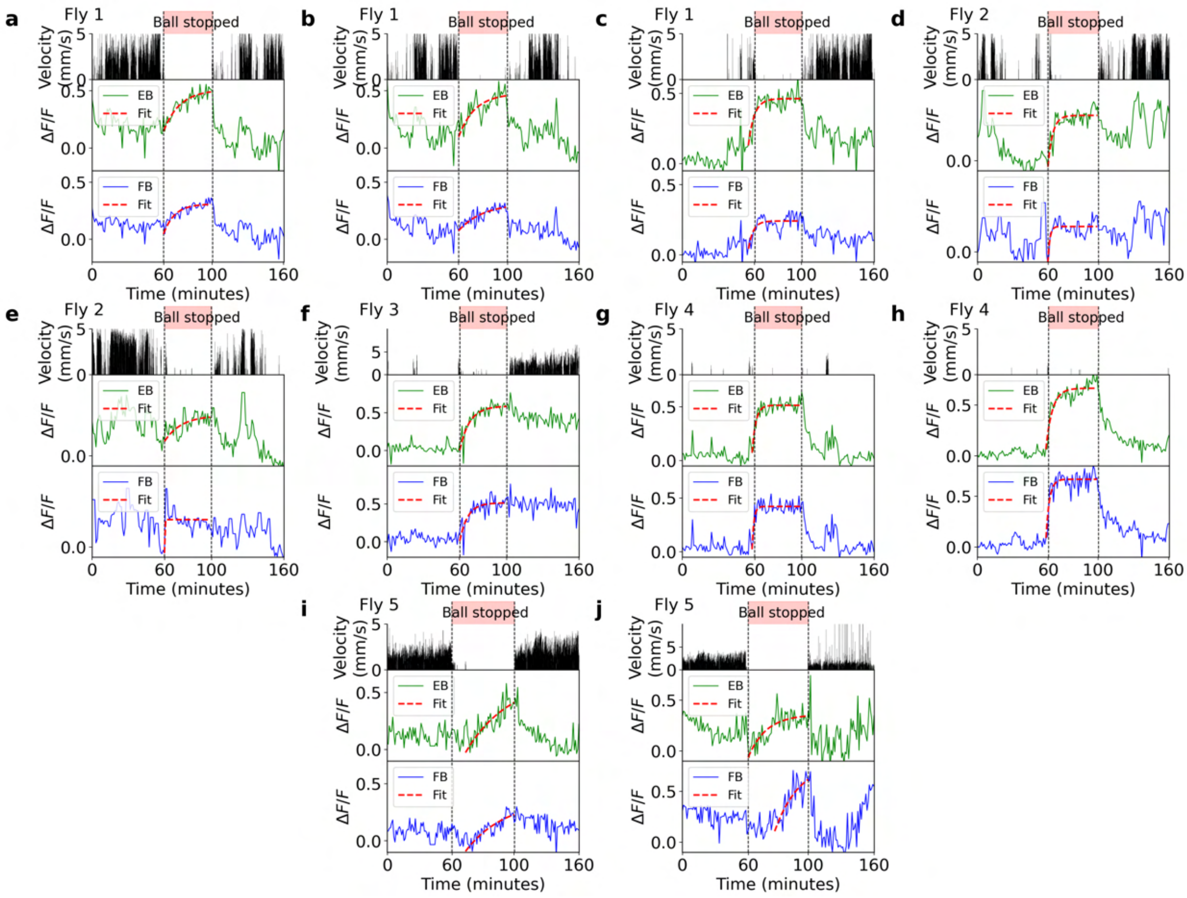

Supplementary Figure S17: Trials where the ball was blocked during recordings in EG. **a** First row: time where the ball was stopped (red region) to induce persistent behavioral activity (pushing and pulling of the ball, but not walking, see Methods). Second row: velocity of fly. Third and fourth row: Calcium activity of EG in EB (green) and FB (blue), respectively. Red lines are exponential fits. **b-j** Same as a. Each panel represents a different trial and the fly from which each trial was recorded is shown in the left top corner of each panel. See Fig. 1g for ROIs.

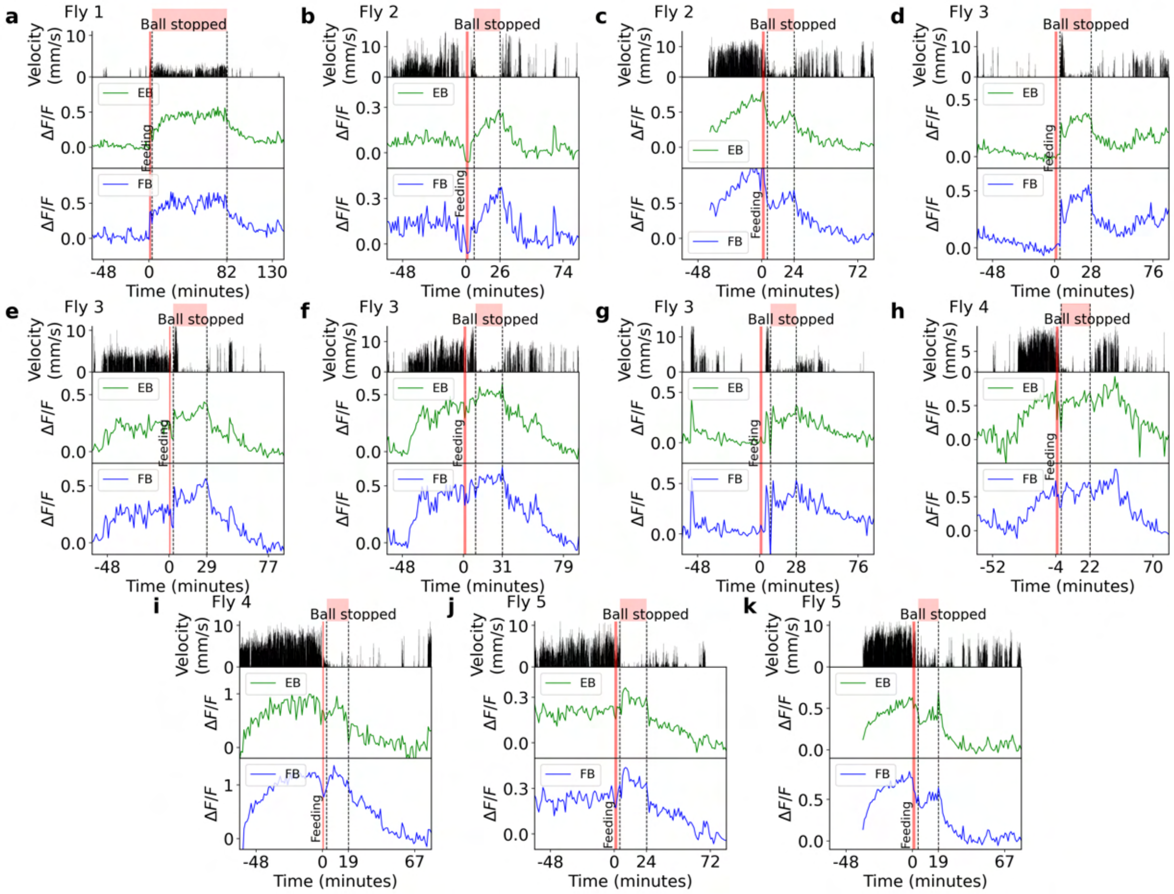

Supplementary Figure S18: Trials where the ball was blocked after feeding to induce persistent behavioral activity (pushing and pulling of the ball, but not coordinated walking, see Methods) while activity in glia was recorded. **a** First row: time where the ball was stopped (red region). Second row: velocity of the fly. Third and fourth row: Calcium activity of EG in EB (green) and FB (blue). The vertical red line indicates feeding. **b-k** Same as a. Each panel represents a different trial. The fly from which each trial was obtained is shown in the top left corner of each panel. See Fig. 1g for ROIs.

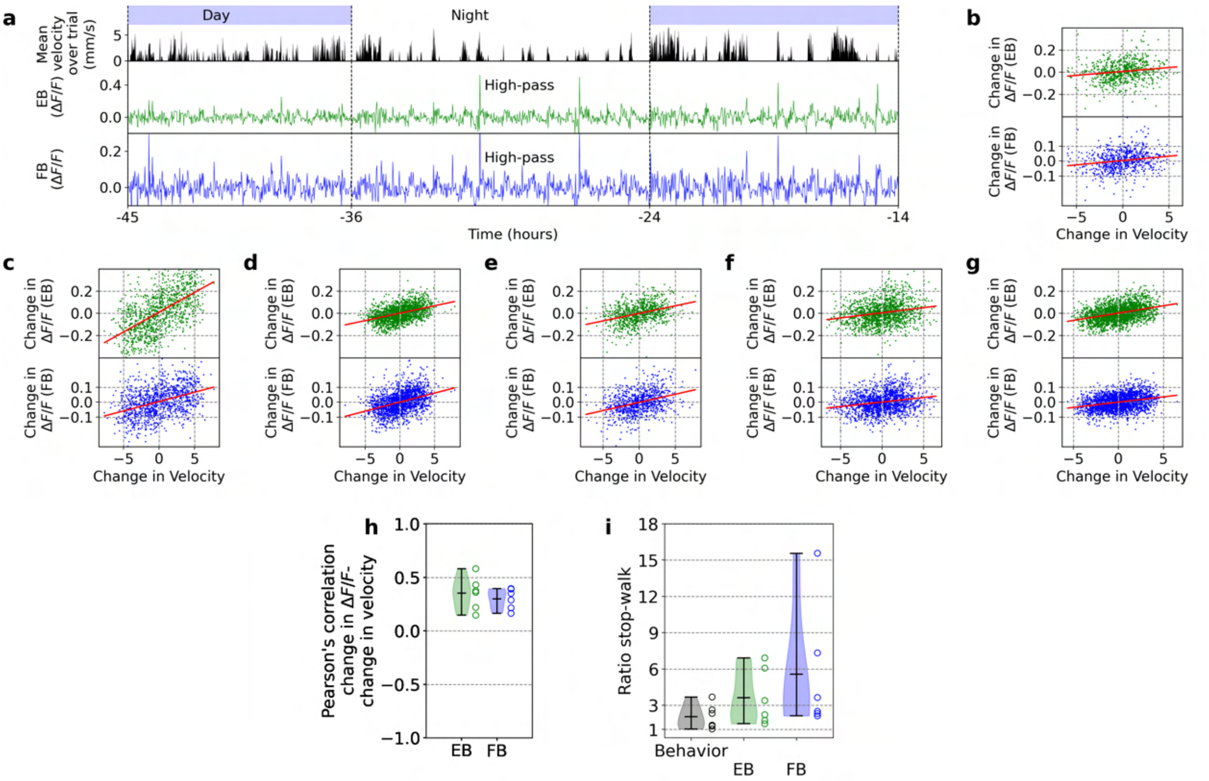

Supplementary Figure S19: **a** Top row: day and night cycle in VR over time. Second row: mean velocity during recording epoch (1 s). Third and fourth row: fluorescence signals in EB and FB, respectively. Fluorescence was filtered with a high-pass filter for periods higher than 0.5 h. **b** Correlation between mean velocity over trial and high-pass filtered fluorescence. **c-g** Same as b for 5 more flies. **h** Ratios of time in 'stop' and 'walk' states (black) and ratio of time constants between 'stop' and 'walk' states in EB (green) and FB (blue) for each fly ($n = 6$). Ratios were not significantly different, p-values were above 0.05 using t-test. **i** Correlation coefficients as in e for $n = 6$ flies. All flies had p-values lower than 0.05 (see Supplementary table S4.

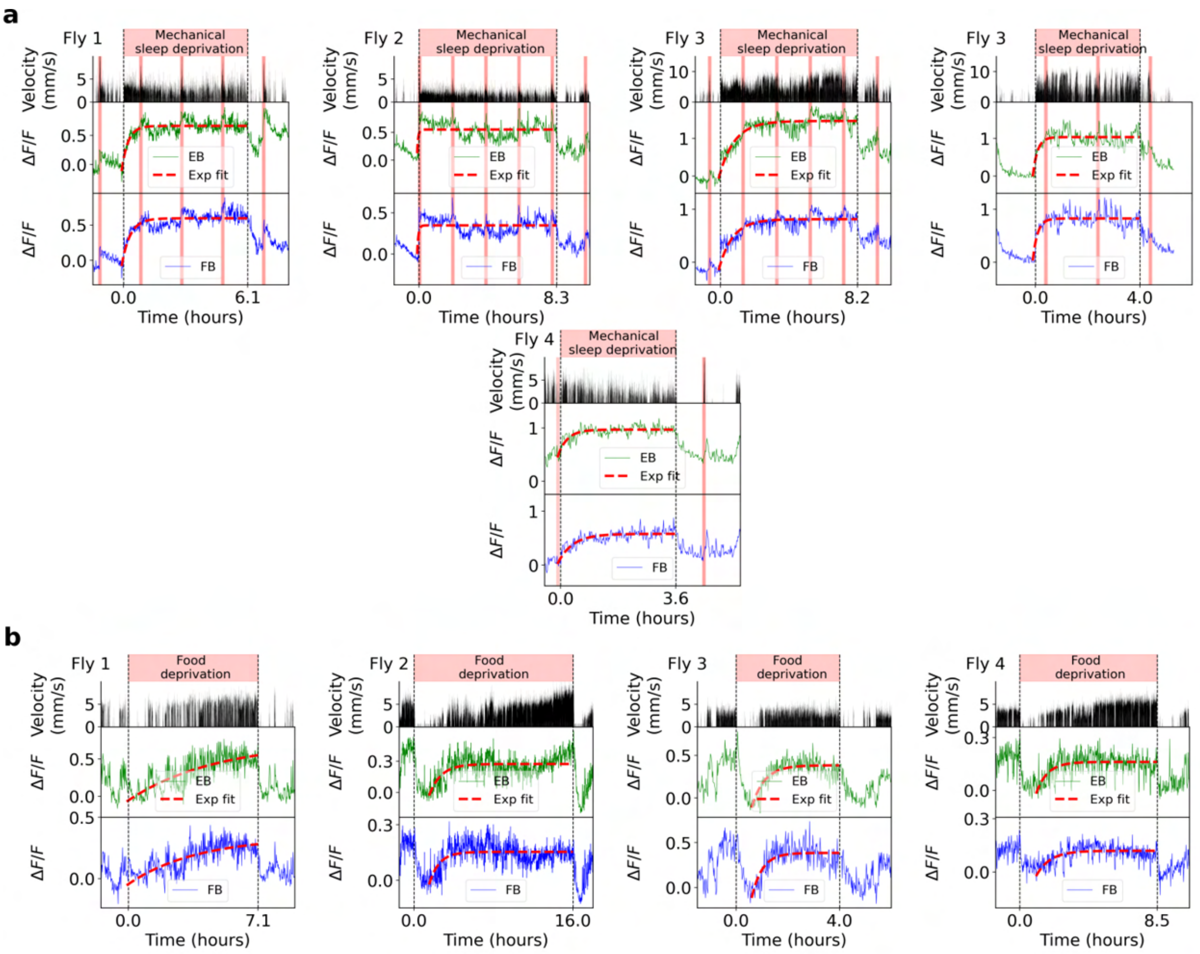

Supplementary Figure S20: Glia activity during mechanical sleep deprivation (a) and food deprivation (b) in each trial. **a** Each panel shows the velocity over time (second row), and glia activity in EB (third row) and FB (fourth row) during mechanical sleep deprivation (first row) for 5 trials. Vertical red lines indicate feeding events and exponential fits (red) are shown for visualization of saturation levels. The fly from which each trial was obtained is indicated in the top left corner of each panel. **b** Same as a, but during food deprivation (which leads to hyperactivity), with a different set of flies.

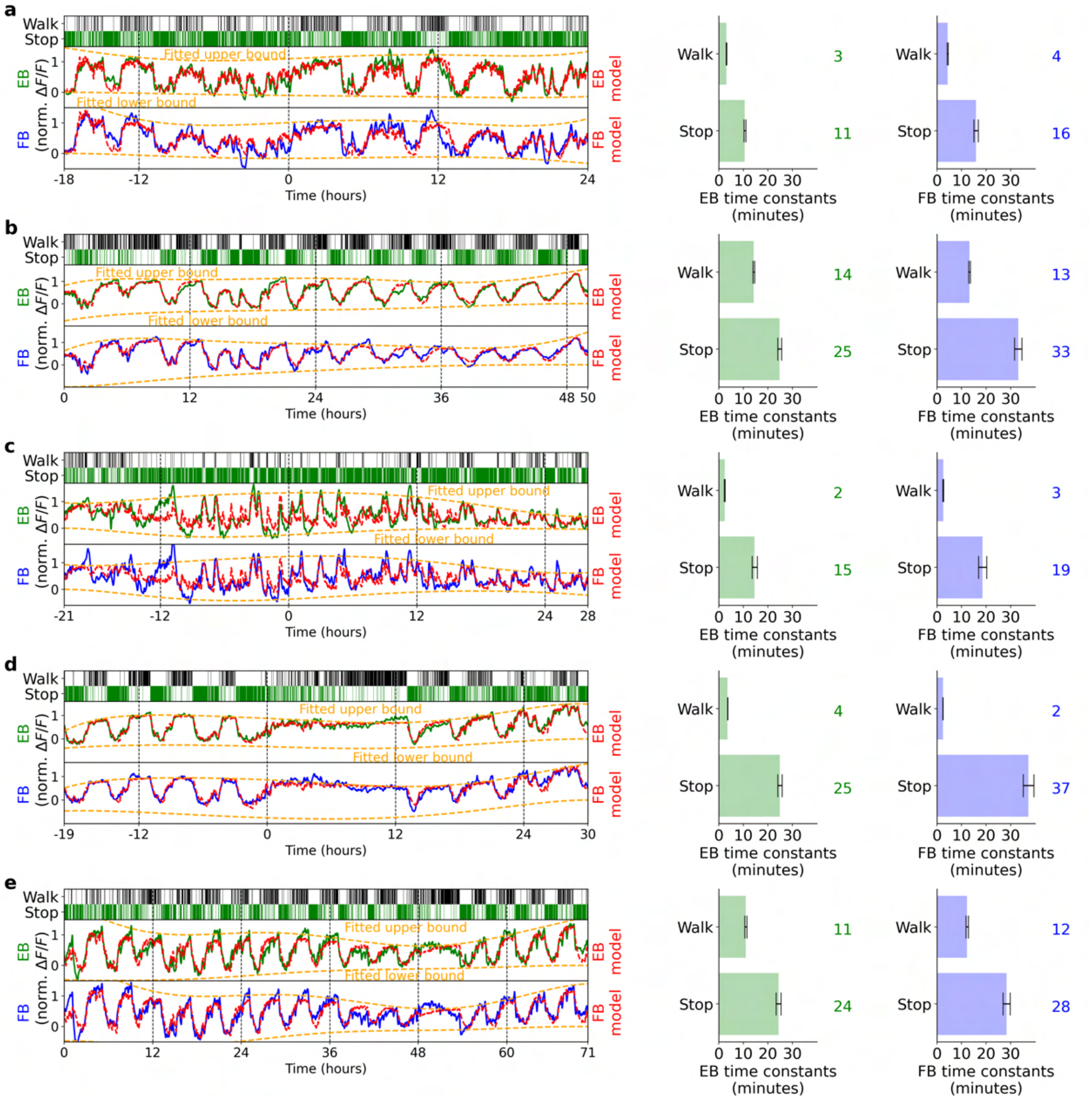

Supplementary Figure S21: Fitting glia activity in EB and FB with homeostat 2-state model. **a** Left side: top row shows walking and stopping activity of a fly (1 s bins). Second and third row: Normalized fluorescence in EB (green) and FB (blue). Red lines show fitted model, while orange lines represent fitted upper and lower bounds of the model. Right side: fitted time constants from EB (green) and FB activity (blue). gray lines indicate error bars of estimated time constants (see Methods). Green and blue numbers show rounded value of the fitted time constants. **b-e** Same as a. Each panel represents a fitted model for each fly.

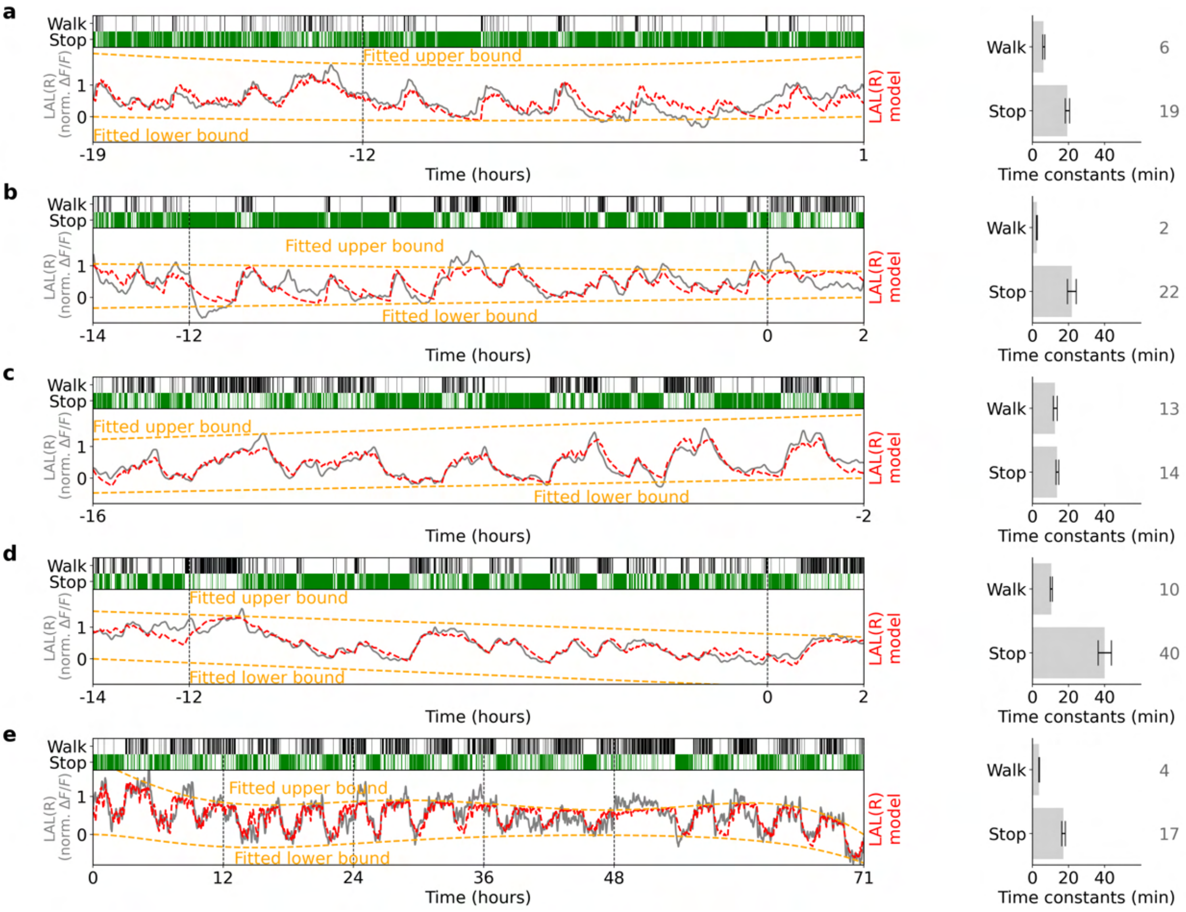

Supplementary Figure S22: Fitting EG calcium activity in the right LAL with homeostat 2-state model. **a** Left side: top row shows walking and stopping activity of a fly (1 s bins). Second and third row: Normalized fluorescence in the LAL (gray). Red lines show fitted model, while orange lines represent fitted upper and lower bounds of the model. Right side: fitted time constants in LAL (gray). Gray lines indicate error bars of estimated time constants (see Methods). Gray numbers show rounded value of fitted time constants. **b-e** Same as a. Each panel represents a fitted model for each fly. See Extended Data Fig. 1b for ROIs.

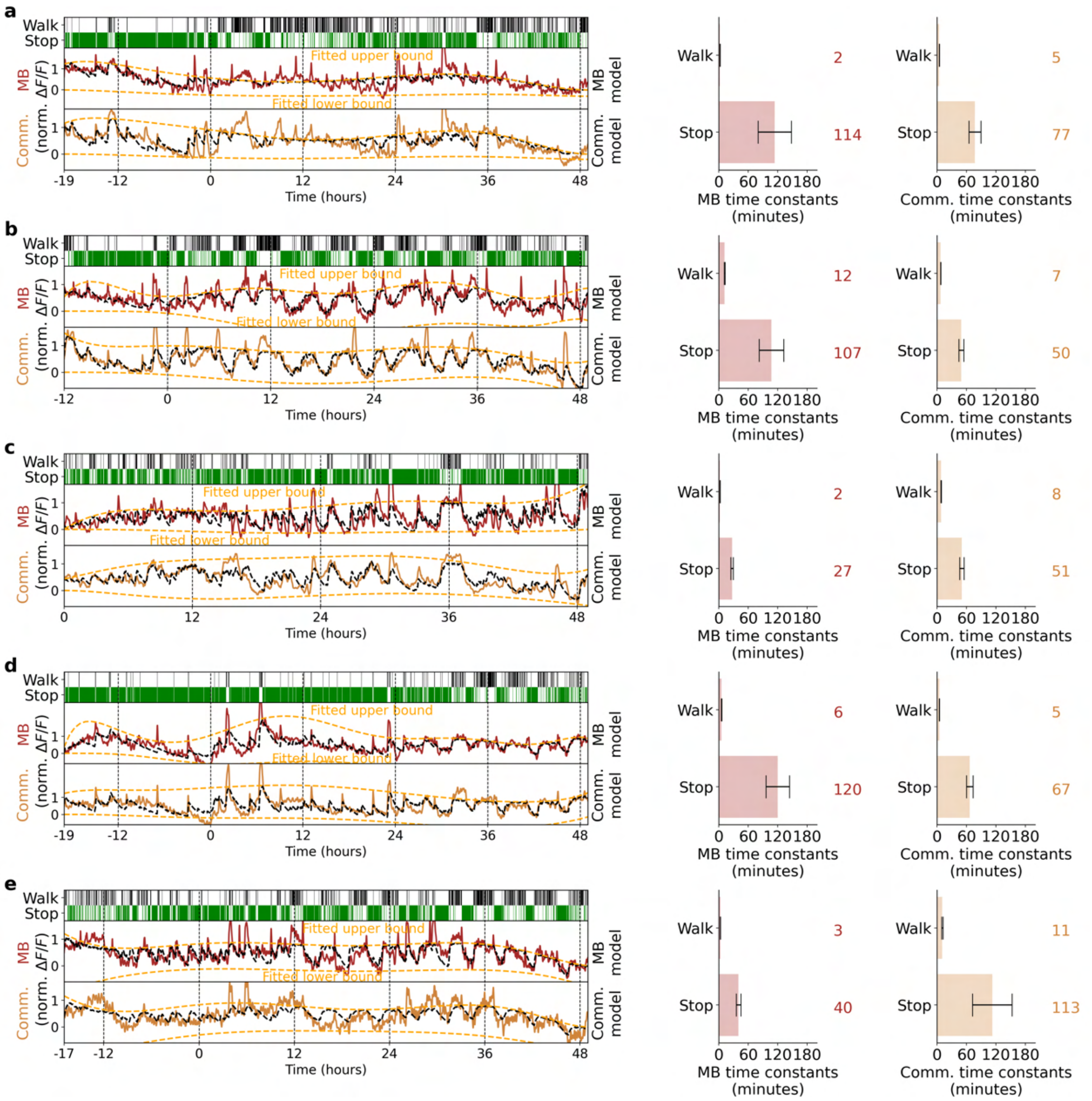

Supplementary Figure S23: Fitting EG calcium activity in MB and midline with homeostat 2-state model. **a** Left side: top row shows walking and stopping activity of a fly (1 s bins). Second and third row: Normalized fluorescence in MB (brown) and midline (dark orange). Black lines show fitted model, while orange lines represent fitted upper and lower bounds of the model. Right side: fitted time constants in MB (brown) and midline activity (dark orange). Gray lines indicate error bars of estimated time constants (see Methods). Brown and dark orange numbers show rounded value of the fitted time constants. **b-e** Same as a. Each panel represents a fitted model for each fly. See Extended Data Fig. 1c for ROIs.

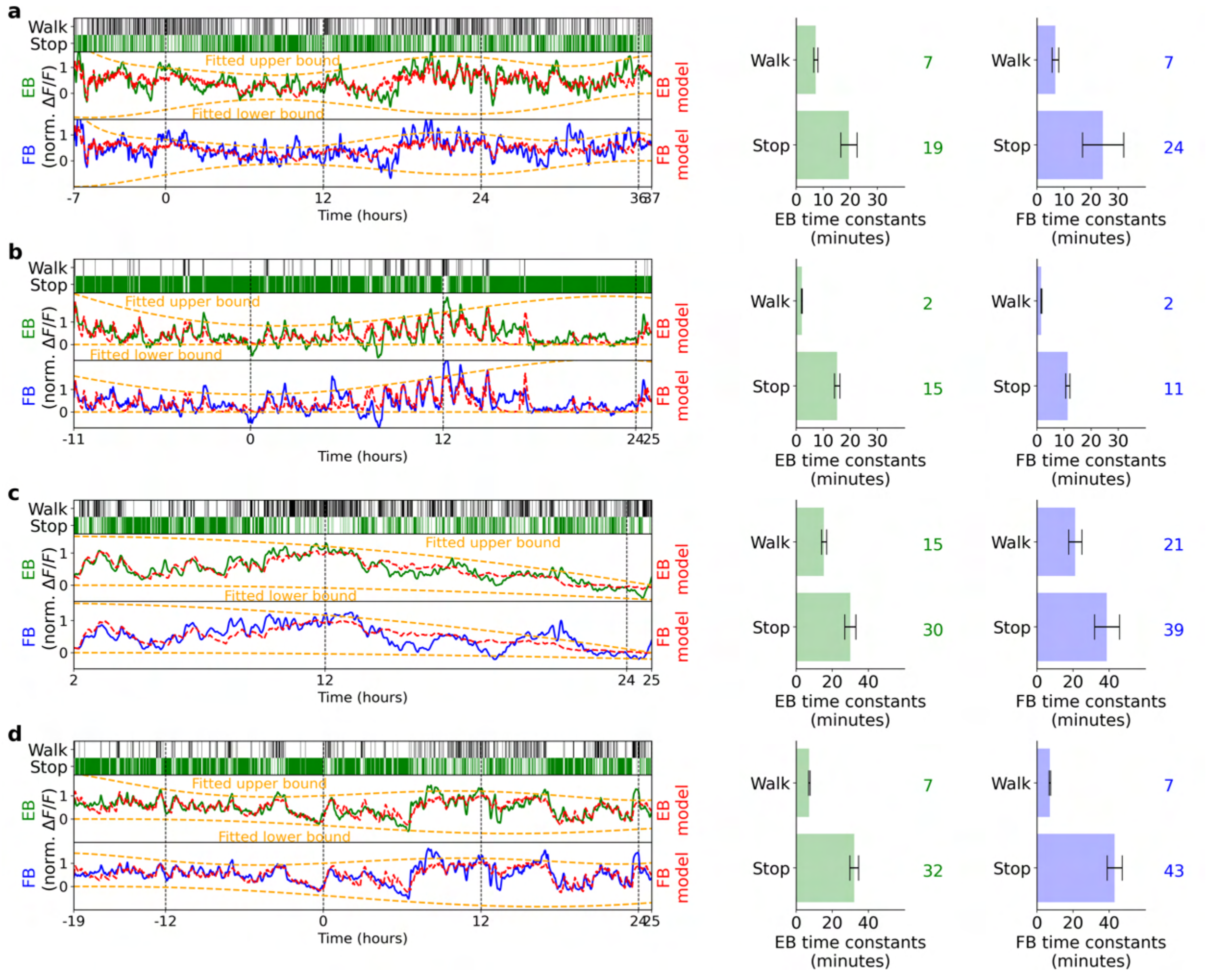

Supplementary Figure S24: Fitting AL activity in EB and FB with homeostat 2-state model. **a** Left side: top row shows walking and stopping activity in 1 s bins of a fly. Second and third row: Normalized fluorescence in EB (green) and FB (blue). Red lines show fitted model, while orange lines represent fitted upper and lower bounds of the model. Right side: fitted time constants from EB (green) and FB activity (blue). Gray lines indicate error bars of estimated time constants (see Methods). Green and blue numbers show rounded value of the fitted time constants. **b-d** Same as a. Each panel represents a fitted model for each fly. ROIs as in Fig. 1g.

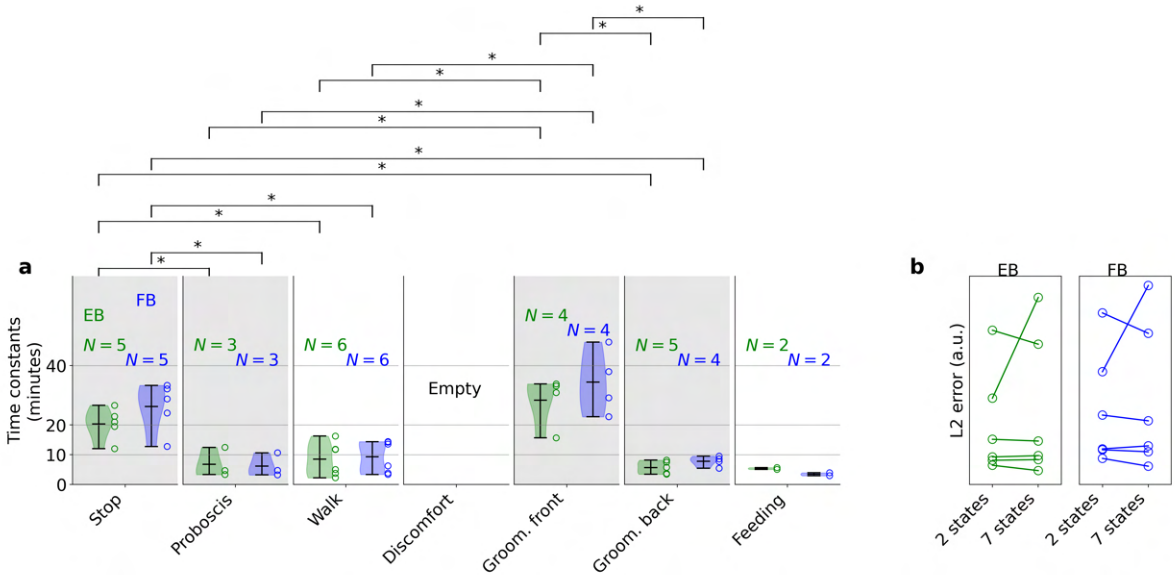

Supplementary Figure S25: Significance levels for behaviors in 7-state model. **a** Time constants of the 7 classified behaviors fitted in 7-state model for EB (green) and FB (blue) from 6 flies. See Extended Data Fig. 5b for an example of model fitting for a single fly. Only time constants with an error of less than 20% were included in the histograms (see Methods). Asterisks indicate statistical significance between all possible pairs of fitted time constants (two-sided t-test, $p < 0.05$). All p-values were corrected using Benjamini–Hochberg procedure (see Supplementary Data S1 for detailed statistics). **b** Comparison between the 2-state and 7-state models in EB (left) and FB (right). L2-error between fit and data for $n = 6$ flies.

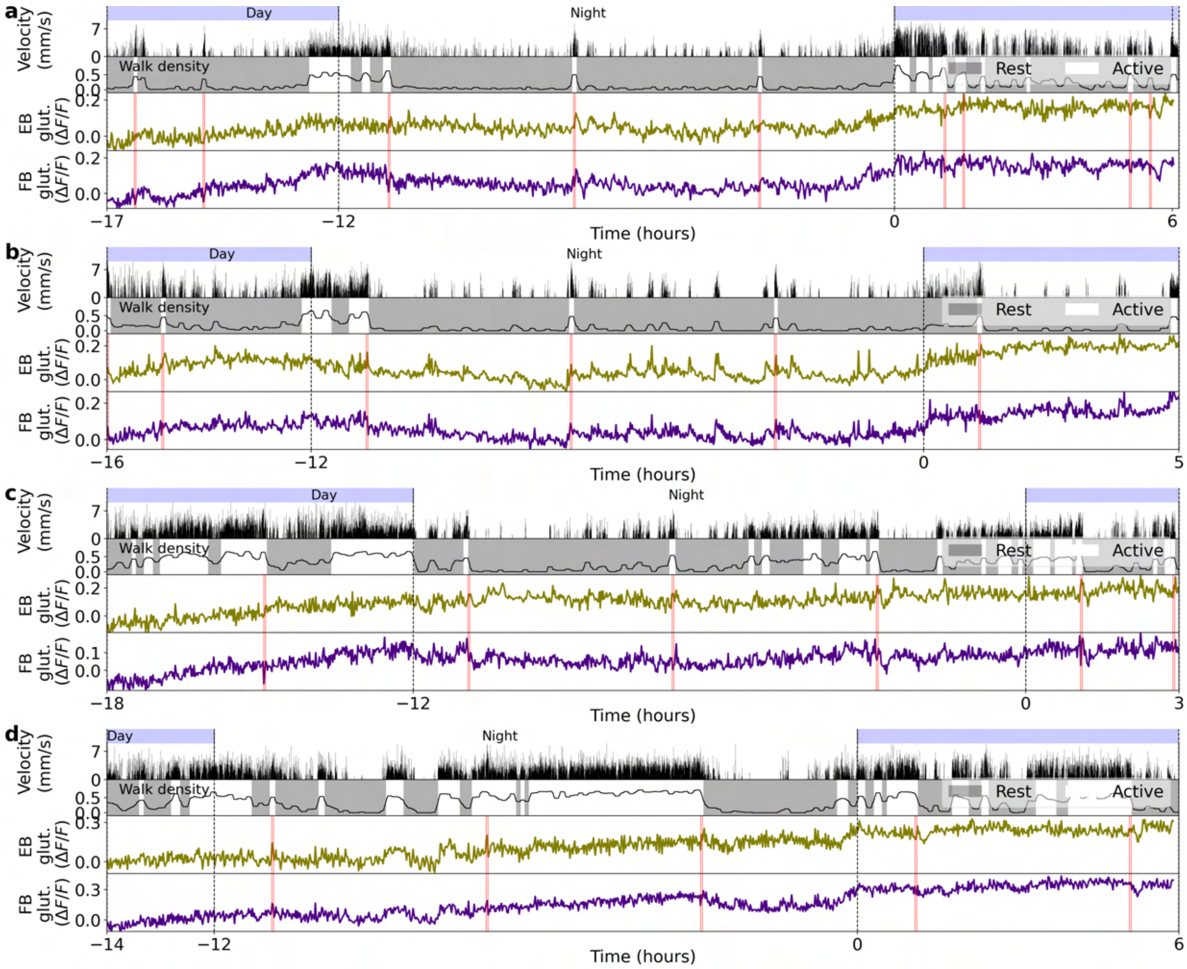

Supplementary Figure S26: Recordings using the glutamate sensor iGluSnFR expressed in EG in four more flies. **a** Top row: day and night cycle in VR. Second row: velocity of the fly in 1 s bins. Third row: walk density (see Methods) and rest (gray region) and active (white region) epochs. Fourth and fifth row: Calcium activity of EG in EB (green) and FB (blue). Thick lines indicate a low-pass filter with a 0.1 h cut-off period. Vertical red lines represent feeding events. **b-d** Same as a. Each panel shows a different fly. ROIs were selected similarly as for calcium imaging, see Fig. 1g.

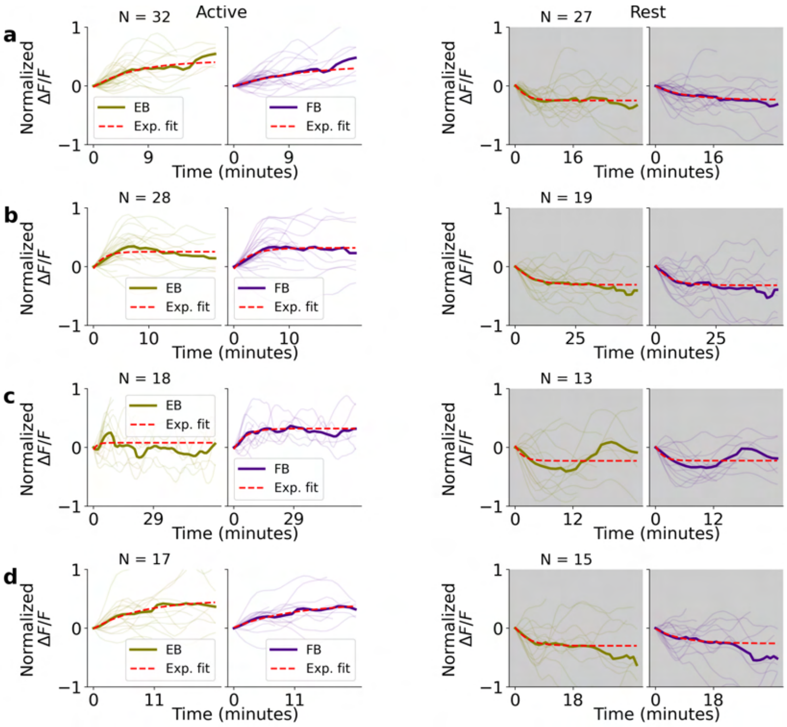

Supplementary Figure S27: Normalized fluorescence traces recorded with glutamate sensor during active and rest epochs for 4 flies. **a** Left side: single (thin lines) and average (thick lines) normalized fluorescence traces in EB (olive) and FB (indigo) during active epochs. Red lines indicate exponential fit. Right side (gray background): same as left side, but during rest epochs. **b-d** Same as a. Each panel is from a different fly, corresponding to Supplementary Fig. S26. ROIs were selected similarly as for calcium imaging, see Fig. 1g.

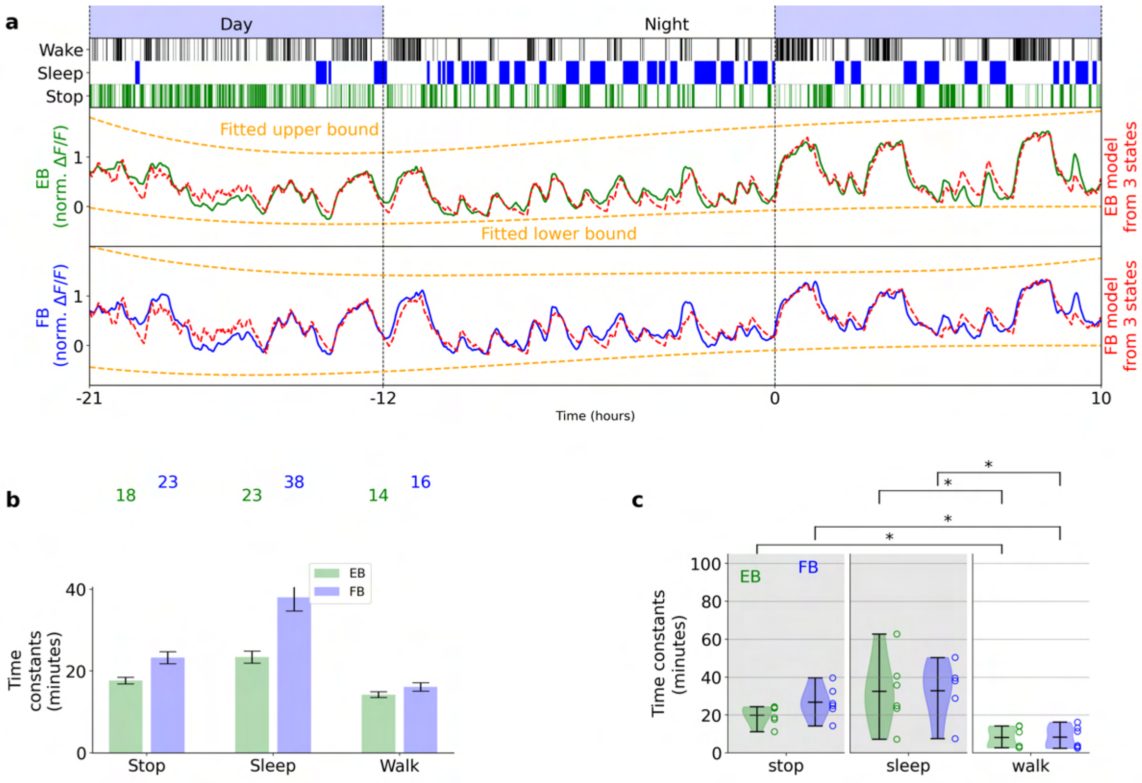

Supplementary Figure S28: Fitting glia activity with homeostat 3-state model defining a 'sleep' state as epochs where the fly was stopped for more than 5 min. **a** First row shows day and night cycle in VR, second row shows behavior of fly 1 extracted from ball velocity. Sleep is defined as epochs where the fly stops walking for at least 5 min. The third and fourth rows show fitting of the 3-state model (red line) and corresponding bounds (orange lines) in EB and FB, respectively. **b** Time constants of 3 states resulting from fitting in a for EB (green) and FB (blue) in a single fly. **c** Distribution of time constants for the 3 states for $n = 6$ flies. Asterisks represent statistical significance between states using two-sided t-tests ($p < 0.05$). All p-values are corrected using the Benjamini–Hochberg procedure (see Supplementary Data S1 for detailed statistics). See also Supplementary Fig. S29 for comparison.

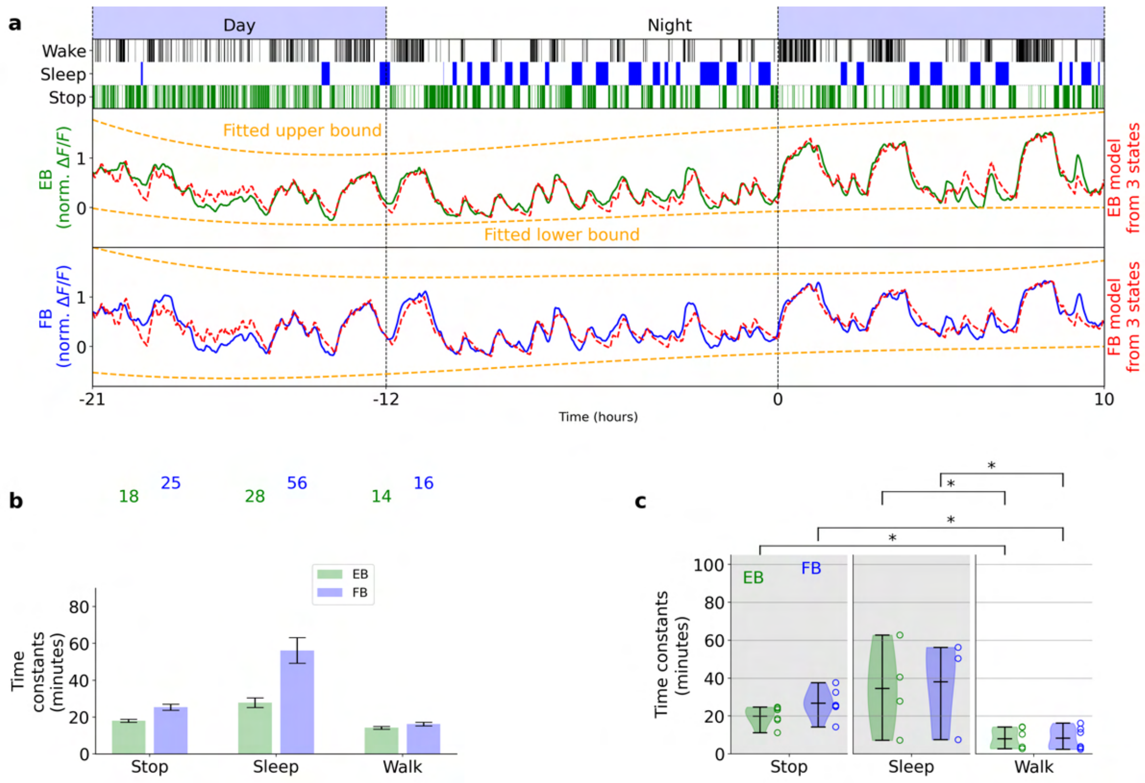

Supplementary Figure S29: Fitting glia activity with homeostat 3-state model with a 'sleep' state define as only after 5 min of immobility. **a-c** as in Supplementary Fig. S28. Different from Supplementary Fig. S28 the second state was defined to start only after 5 min of rest, not including the first 5 min of rest.

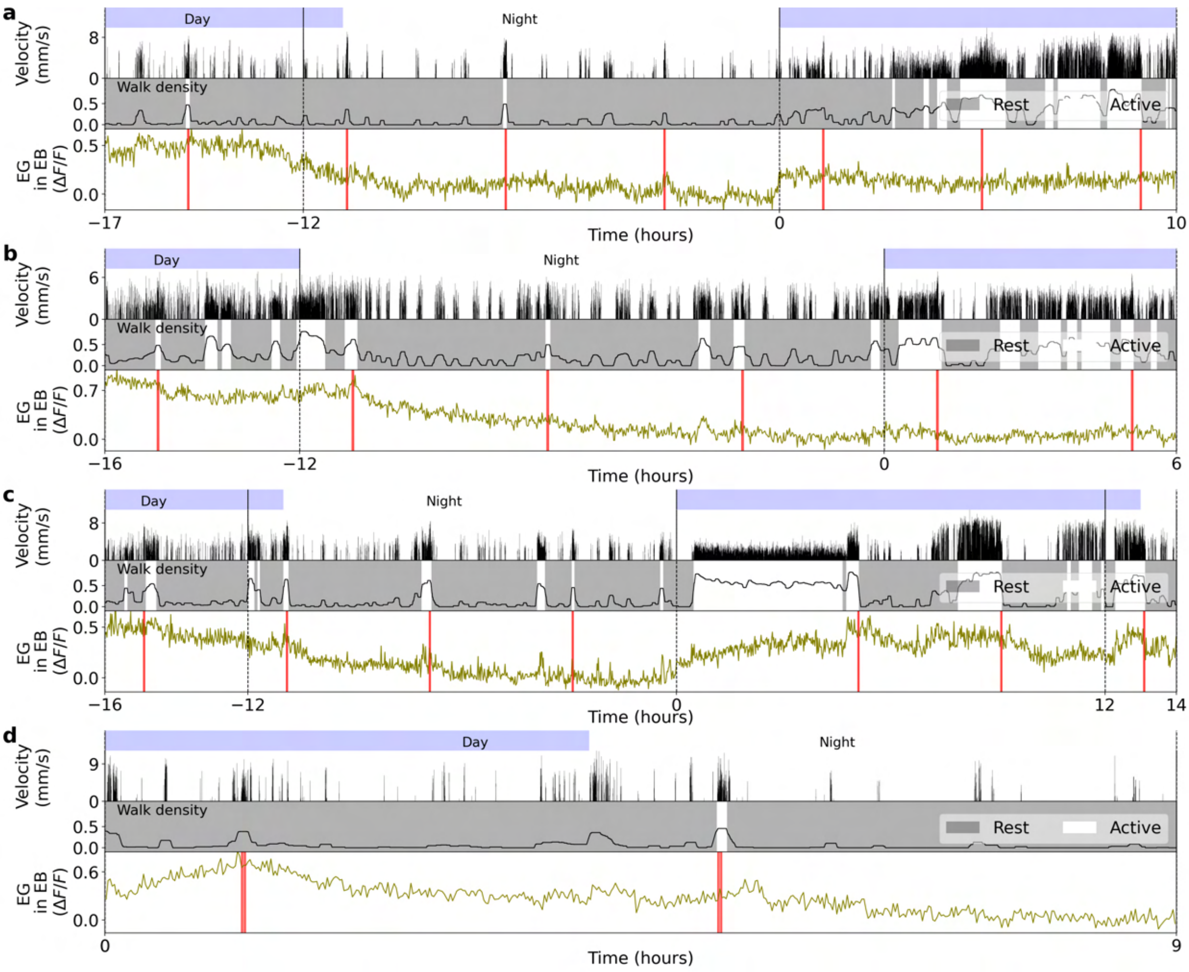

Supplementary Figure S30: Long-term imaging of calcium activity in R5 neurons labeled by 58H05-GAL4 in four flies. **a** Top row: day and night cycle in VR. Second row: velocity of the fly in 1 s bins. Third row: walk density (see Methods), rest (gray region), and active (white region) epochs. Fourth row: Calcium activity of R5 neurons. The thick line shows low-pass filtered data with a 0.1 h cut-off period. Vertical red lines represent feeding events. **b-d** Same as a. Each panel shows a different fly. See Extended Data Fig. 1d for ROIs of ring neurons.

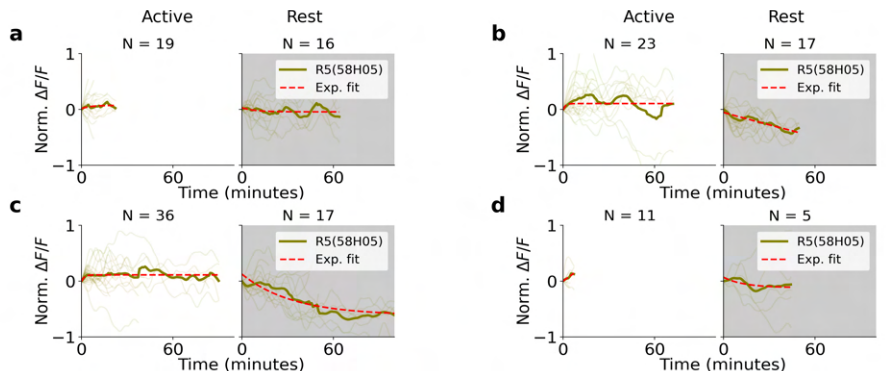

Supplementary Figure S31: Normalized fluorescence traces during active and rest epochs for four flies in R5 neurons (labeled by 58H05-GAL4). **a** Left side (gray background): single (thin lines) and average (thick lines) normalized fluorescence traces of activity in R5 neurons during active epochs. Red lines indicate exponential fit. Right side: the same as the left side, but during rest epochs. **b-d** Same as a. Each panel is from a different fly. See Extended Data Fig. 1d for ROIs of ring neurons.

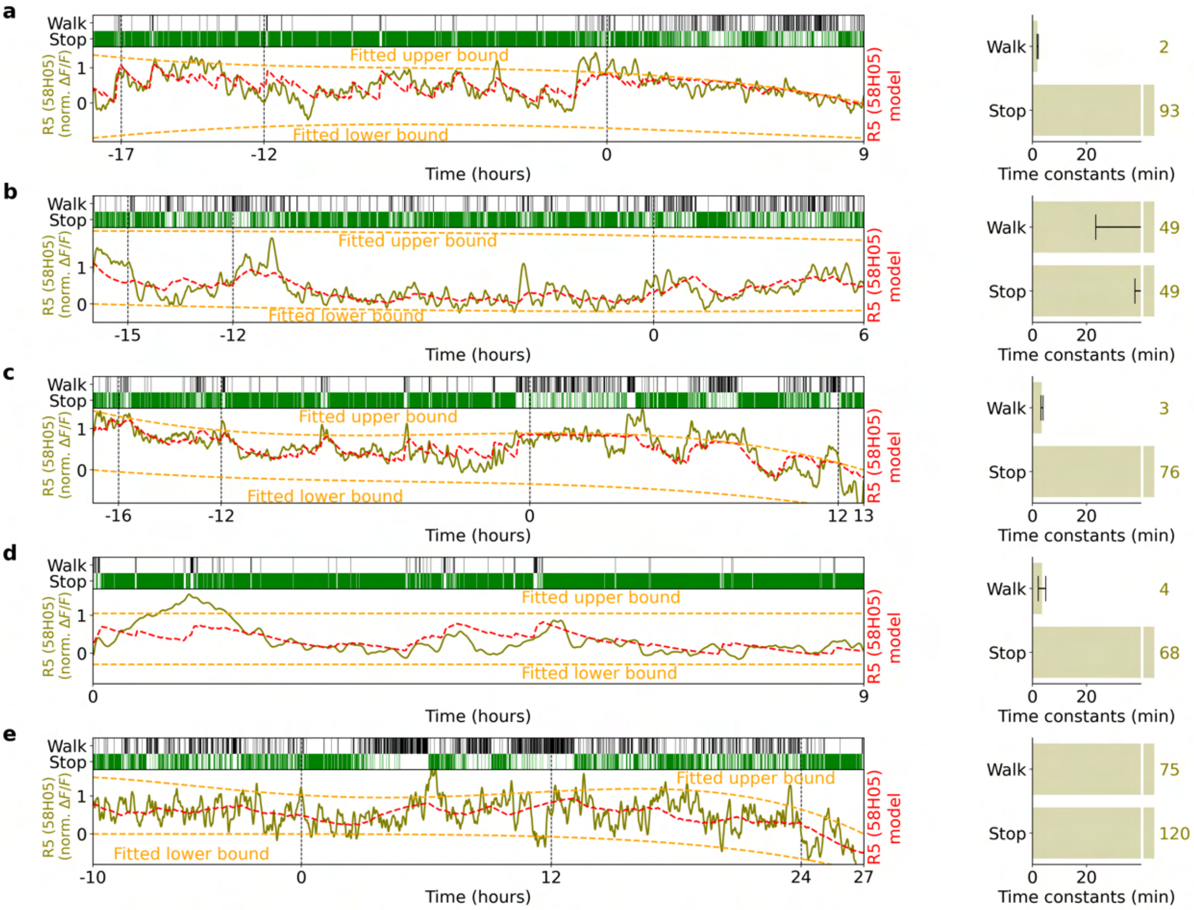

Supplementary Figure S32: Fitting calcium activity of R5 neurons (labeled by 58H05-GAL4) with homeostat 2-state model. **a** Left side: the top row shows walking and stopping activity of a fly in 1 s bins. Second row: Normalized fluorescence of R5 neurons. Red lines show fitted model, while orange lines represent fitted upper and lower bounds of model. Right side: fitted time constants from the model. Gray lines indicate error bars of estimated time constants (see Methods), while colored numbers show rounded values of fitted time constants. **b-e** Same as a. Each panel represents a fitted model for each fly. See Extended Data Fig. 1d for ROIs of ring neurons.

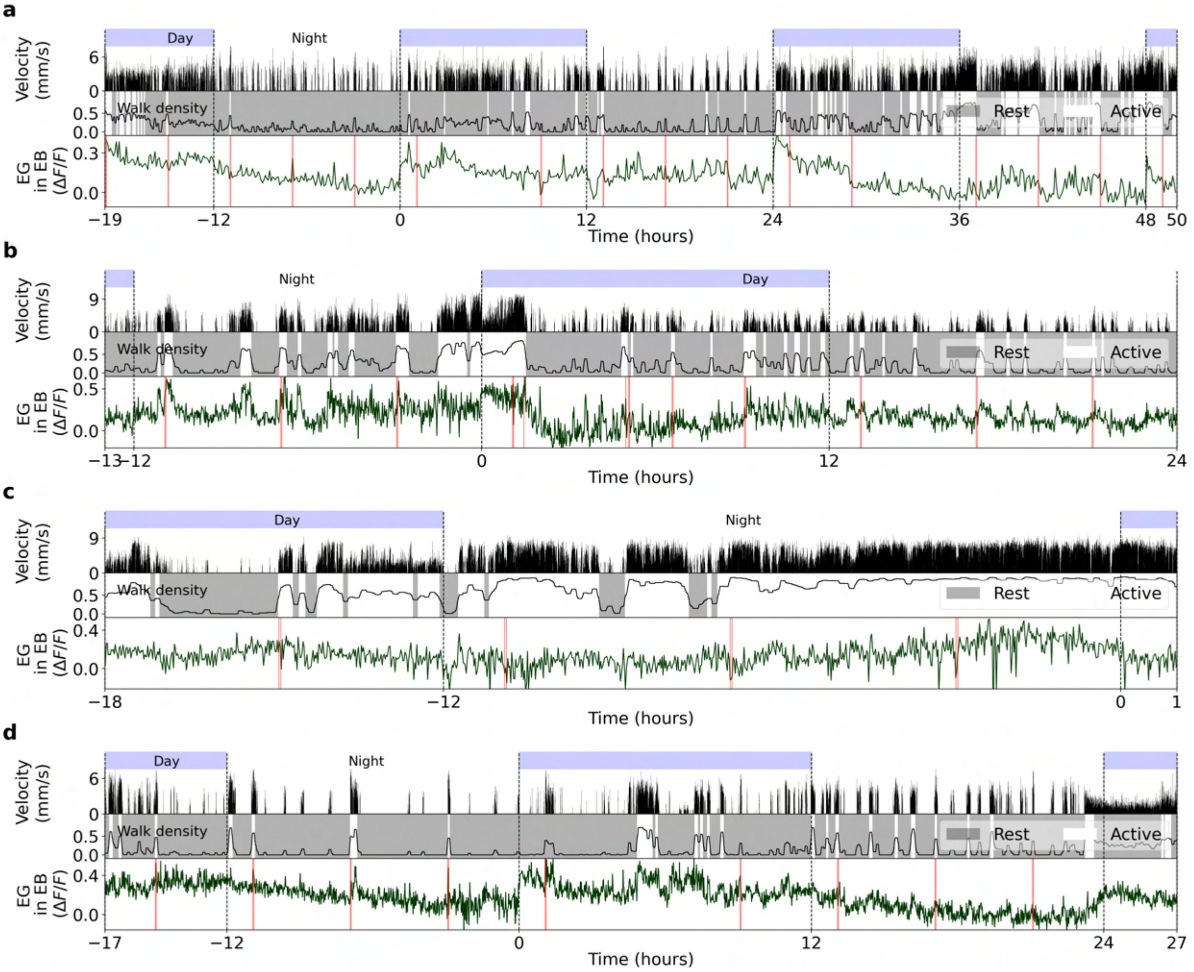

Supplementary Figure S33: Recordings of calcium activity in R5 neurons labeled by GAL4-88F06 in four flies. **a** Top row: day and night cycle in VR. Second row: velocity of fly in 1 s bins. Third row: walk density (see Methods), rest (gray region) and active (white region) epochs. Fourth row: Calcium activity of R5 neurons. Thick line indicates low-pass filtering with a 0.1 h cut-off period. Vertical red lines represent feeding events. See Extended Data Fig. 1e for ROIs of ring neurons. **b-d** Same as a. Each panel shows a different fly. See also next Supplementary Fig. S34

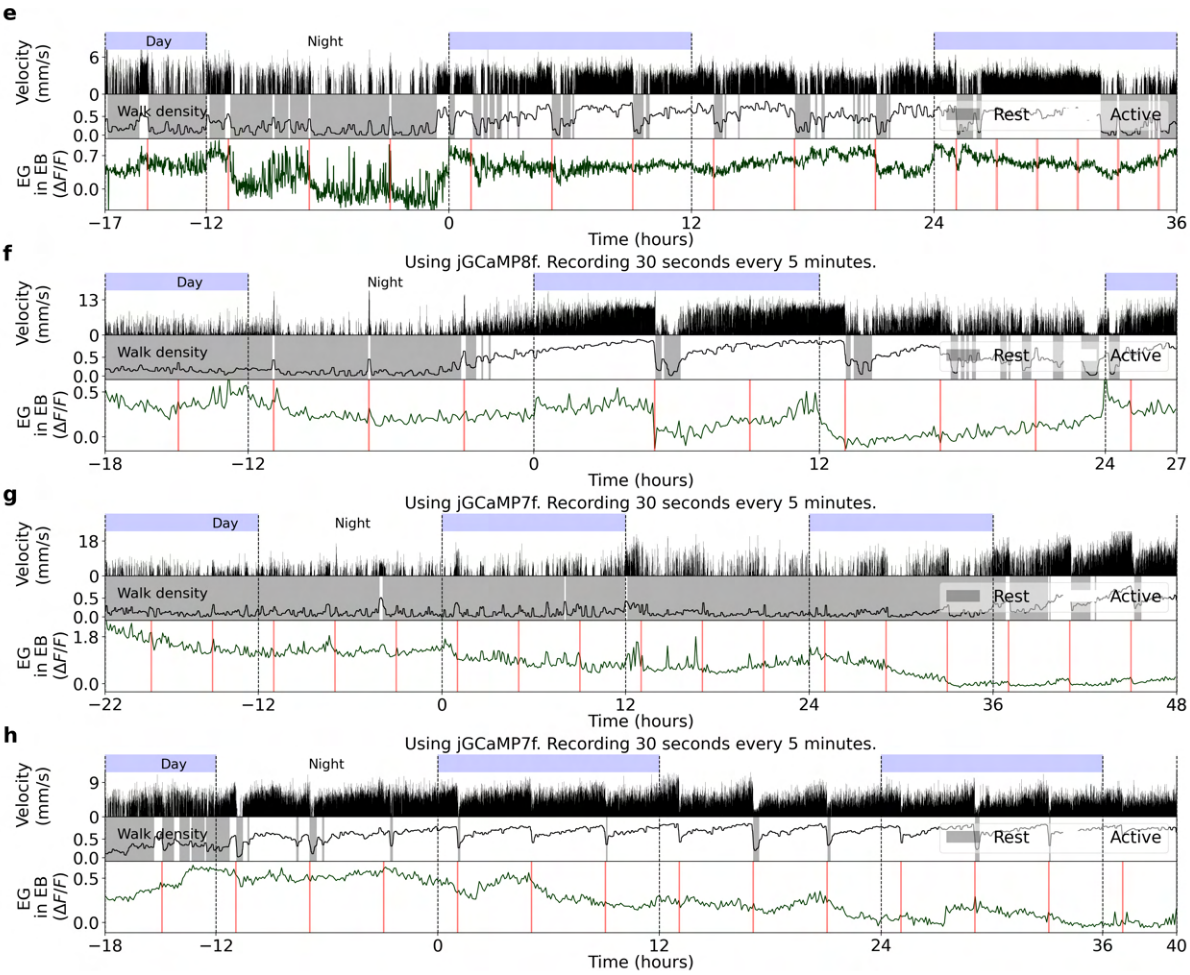

Supplementary Figure S34: Four more recordings of calcium activity in R5 neurons labeled by GAL4-88F06. **e-h** As in and continued from to Supplementary Fig. S33. Continuous imaging at 60 Hz for 30 sec every 5 min is used in f-h (see Methods).

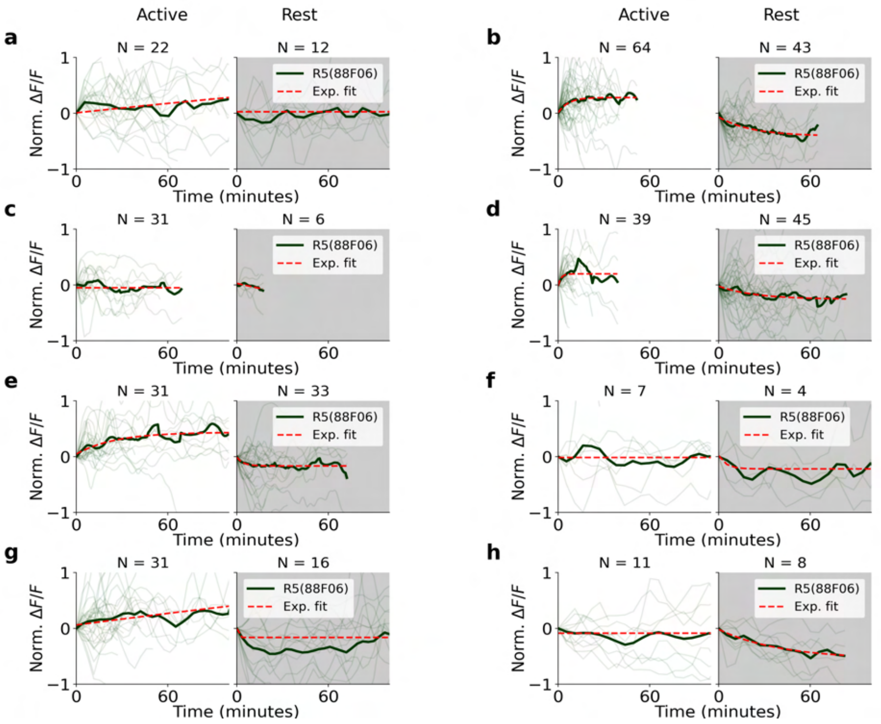

Supplementary Figure S35: Normalized fluorescence traces during active and rest epochs for eight flies in R5 neurons (labeled by GAL4-88F06). **a** Left side: single (thin lines) and average (thick lines) normalized fluorescence traces of activity in R5 neurons during active epochs. Red lines indicate exponential fit. Right side (gray background): the same as left side, but during rest epochs. **b-h** Same as a. Each panel is a different fly corresponding to Supplementary Figs. S34, S33 . See Extended Data Fig. 1e for ROIs of ring neurons.

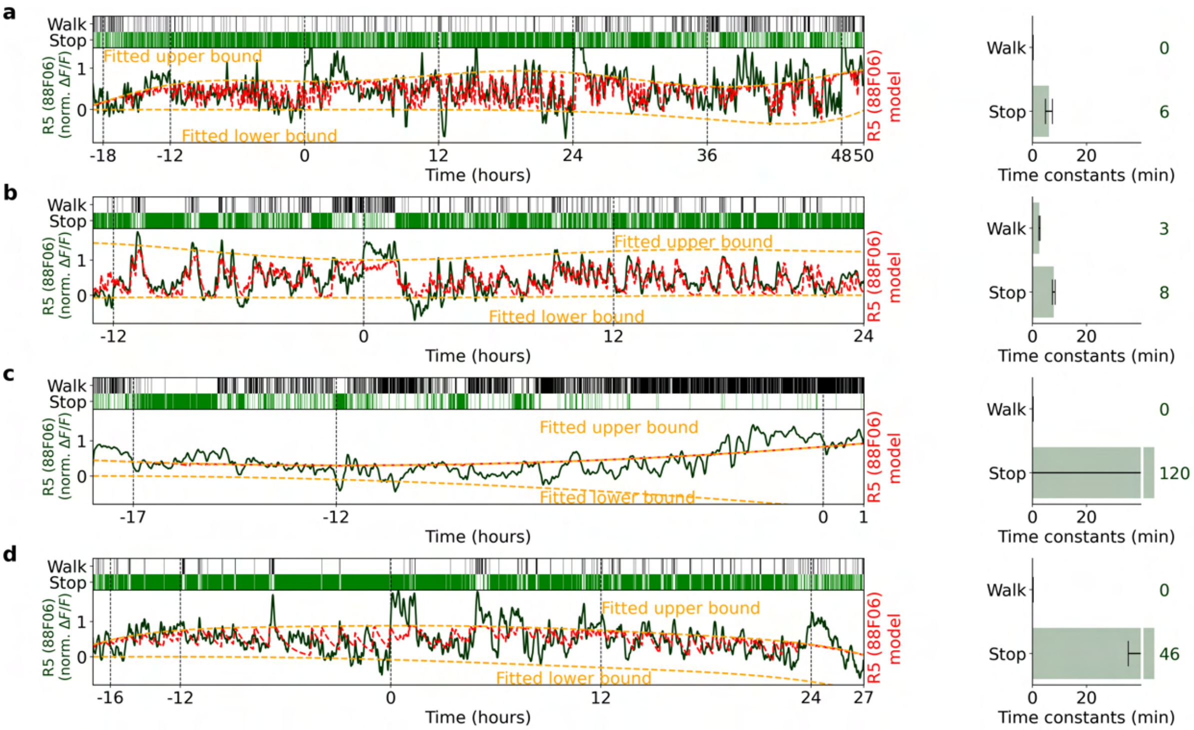

Supplementary Figure S36: Fitting calcium activity of R5 neurons (labeled by GAL4-88F06) with homeostat 2-state model. **a** Left side: top row shows walking and stopping bouts of a fly. Second row: Normalized fluorescence of R5 neurons. Red lines show the fitted model, while orange lines represent fitted upper and lower bounds of model. Right side: time constants obtained from model fitting. Gray lines indicate error bars of estimated time constants (see Methods), while colored numbers show rounded values of fitted time constants. **b-d** Same as a. Each panel represents a fitted model for each fly.

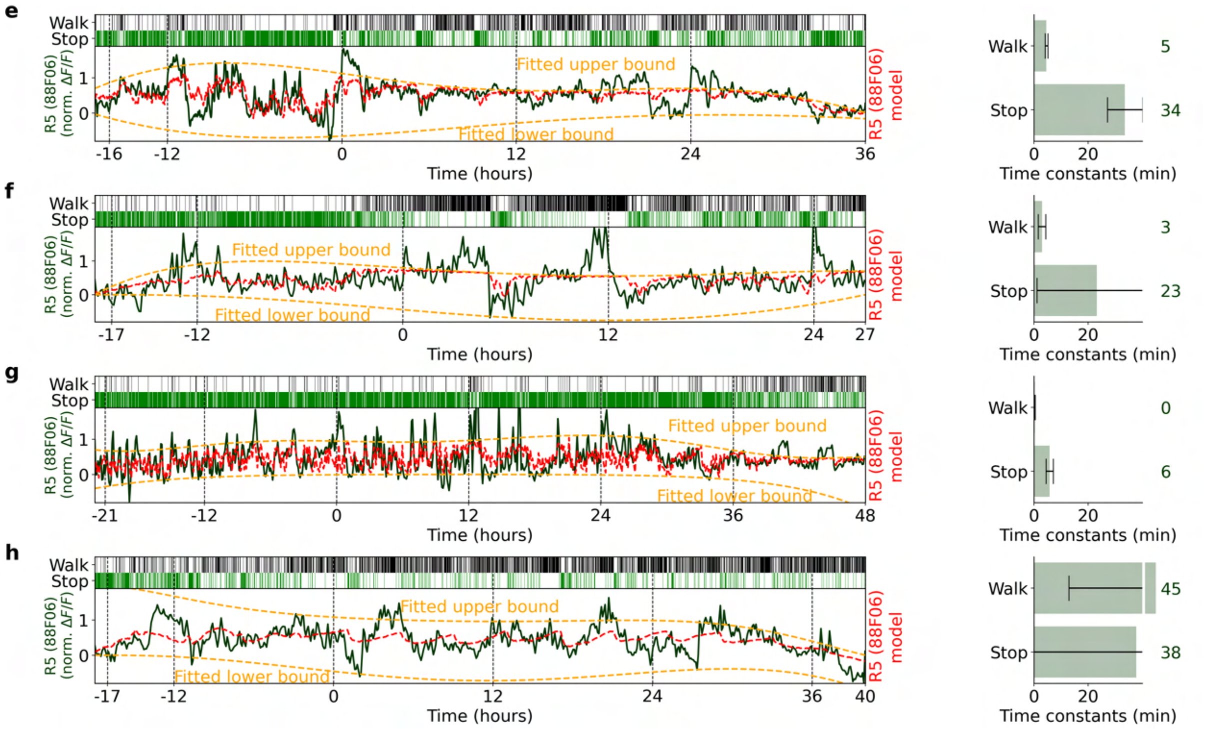

Supplementary Figure S37: Four more recordings and fits of homeostat 2-state model with calcium activity of R5 neurons (labeled by GAL4-88F06). **e-h** As in and continued from Supplementary Fig. S36. See Extended Data Fig. 1f for ROIs of dFB neurons.

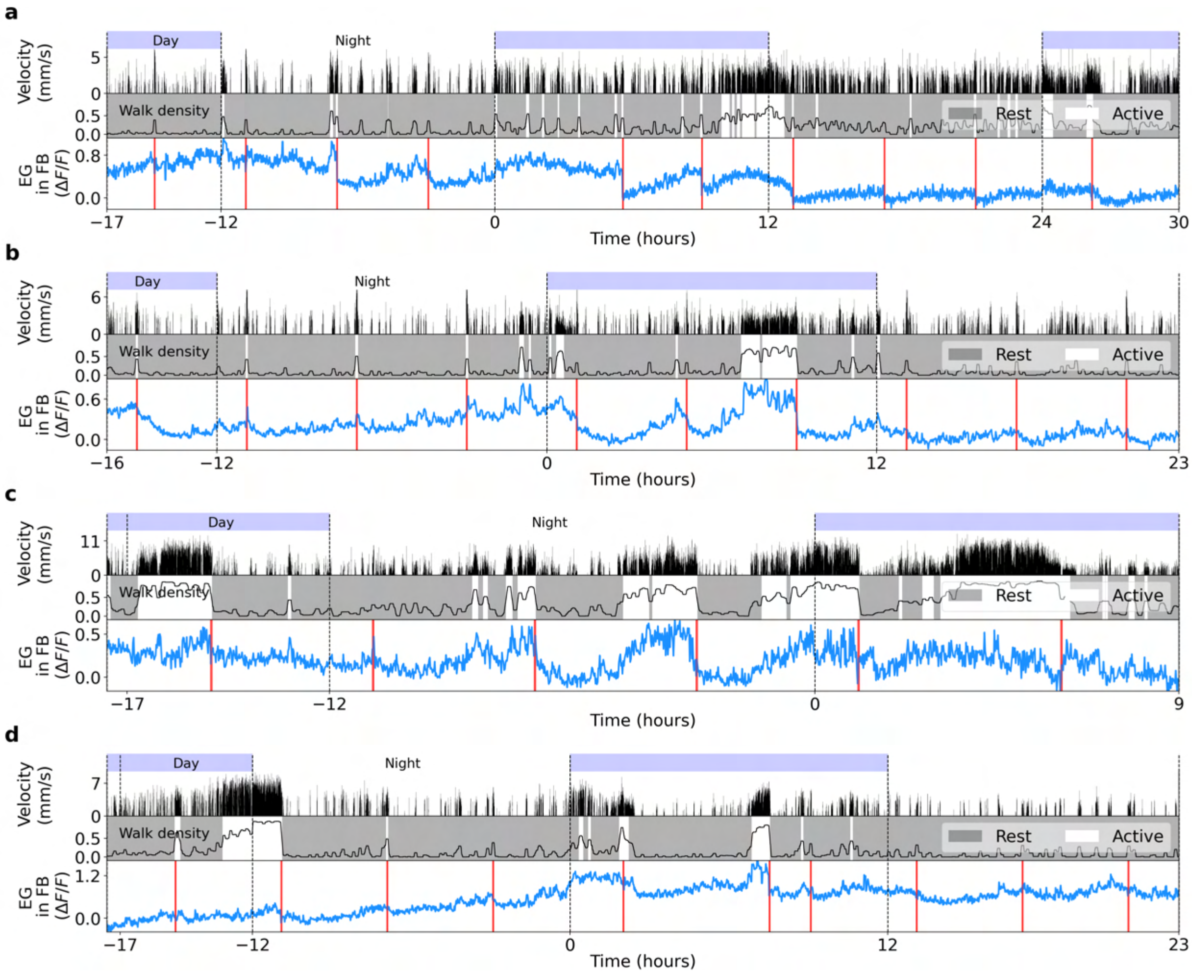

Supplementary Figure S38: Four different recordings of calcium activity in dFB neurons labeled by 23E10-GAL4. **a** Top row: day and night cycle in VR. Second row: velocity of fly in 1 s bins. Third row: walk density (see Methods), rest (gray region), and active (white region) epochs. Fourth row: Calcium activity of dFB neurons. Thick line indicates low-pass filtered data with a 0.1 h cut-off period. Vertical red lines represent feeding events. **b-d** Same as a. Each panel shows a different fly. See also next Supplementary Fig. S39. See Extended Data Fig. 1f for ROIs of dFB neurons.

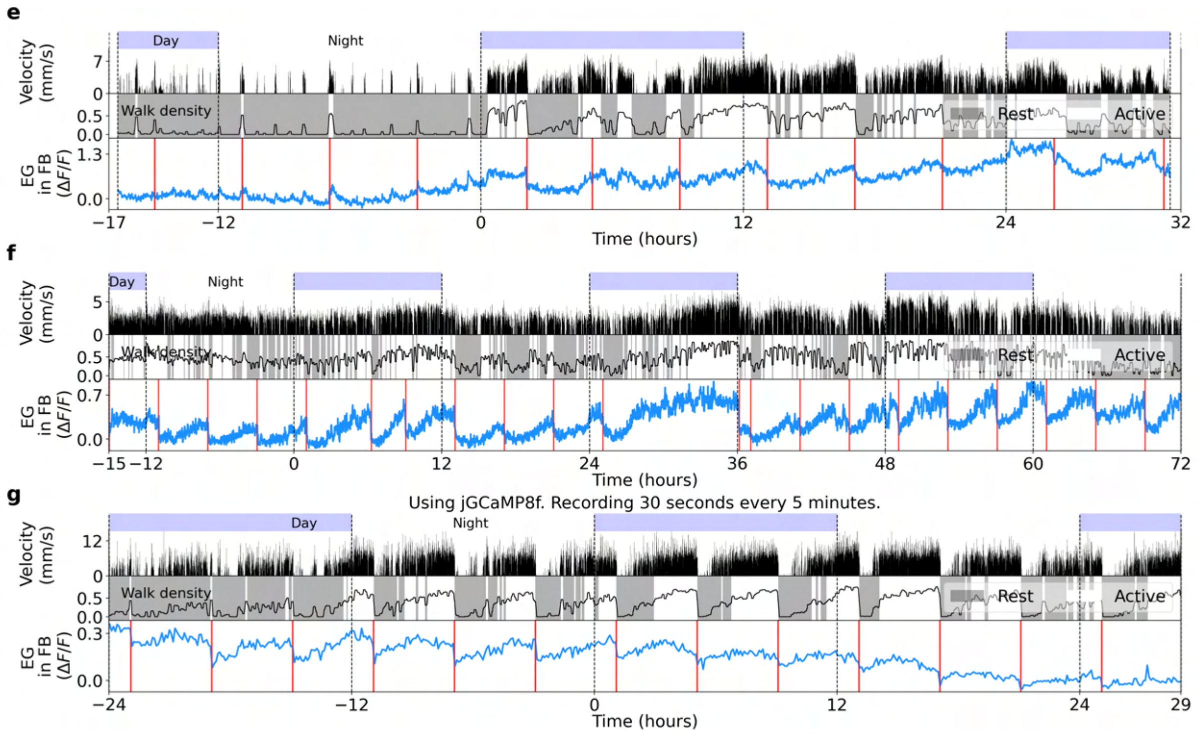

Supplementary Figure S39: Three recordings of calcium activity in dFB neurons labeled by 23E10-GAL4. **e-g** As in and continued from Supplementary Fig. S38.

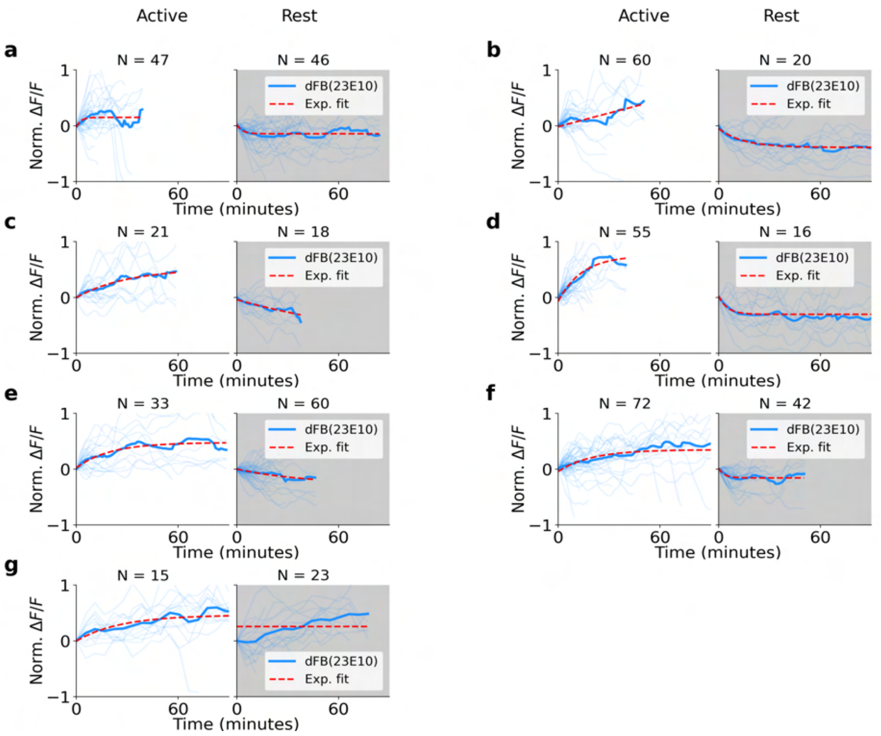

Supplementary Figure S40: Normalized fluorescence traces during active and rest epochs for seven flies in dFB neurons (labeled by 23E10-GAL4). **a** Left side: single (thin lines) and average (thick lines) normalized fluorescence traces of activity in dFB neurons during active epochs. Red lines indicate exponential fit. Right side (gray background): same as the left side, but during rest epochs. **b-f** Same as a. Each panel is from a different fly corresponding to Supplementary Figs. S38, S39.

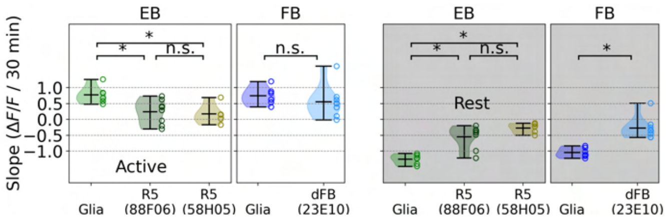

Supplementary Figure S41: Left side: slope of a linear fit between time that flies spent in active epochs and average fluorescence traces from glia and neurons in EB and FB. Right side: same as left side but during resting epochs. Statistical significance was assessed using two-sided t-tests ($p < 0.05$). All p-values are corrected using the Benjamini–Hochberg procedure (for detailed statistics information, see Supplementary Data S1).

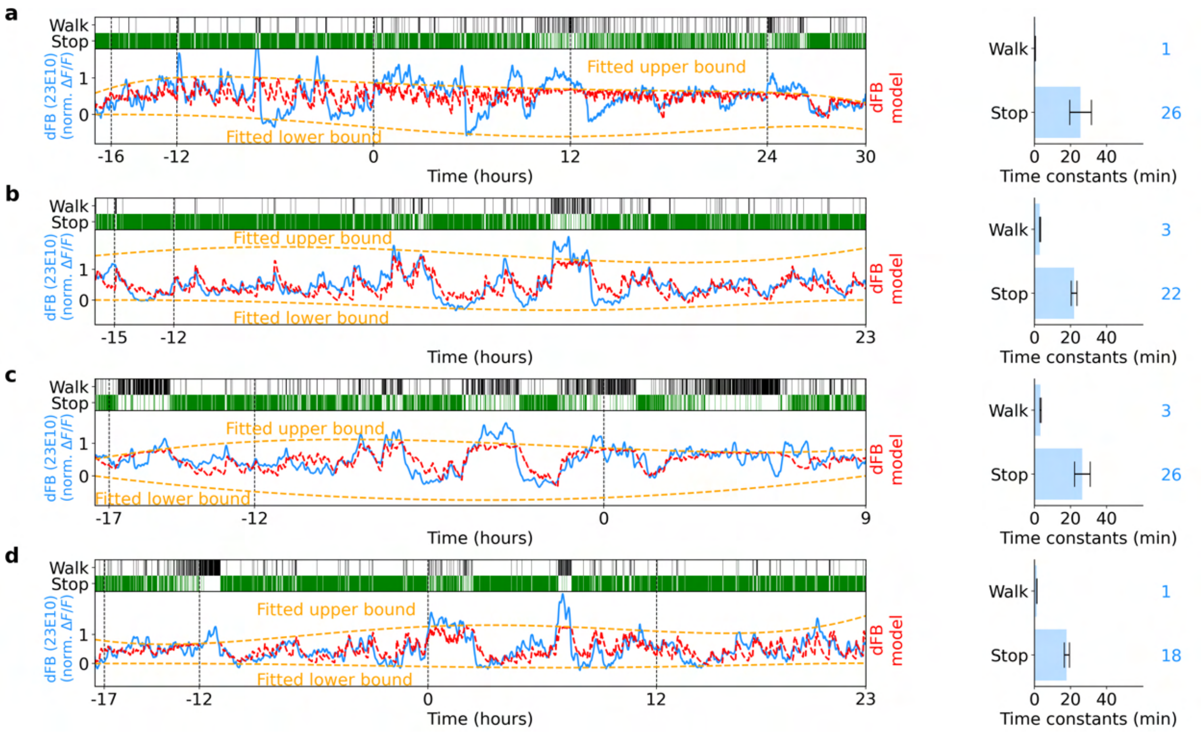

Supplementary Figure S42: Fitting calcium activity of dFB neurons (labeled by 23E10-GAL4) with homeostat 2-state model. **a** Left side, top row: walk and stop activity of a fly (in 1 s time bins). Second row: Normalized fluorescence of dFB neurons. Red lines show fitted model, while orange lines represent fitted upper and lower bounds of model. Right side: fitted model time constants. Gray lines indicate error bars of estimated time constants (see Methods), while colored numbers show rounded values of the fitted time constants. **b-d** Same as a. Each panel represents a fitted model for each fly. See Extended Data Fig. 1f for ROIs of dFB neurons. See also next Supplementary Fig. S43

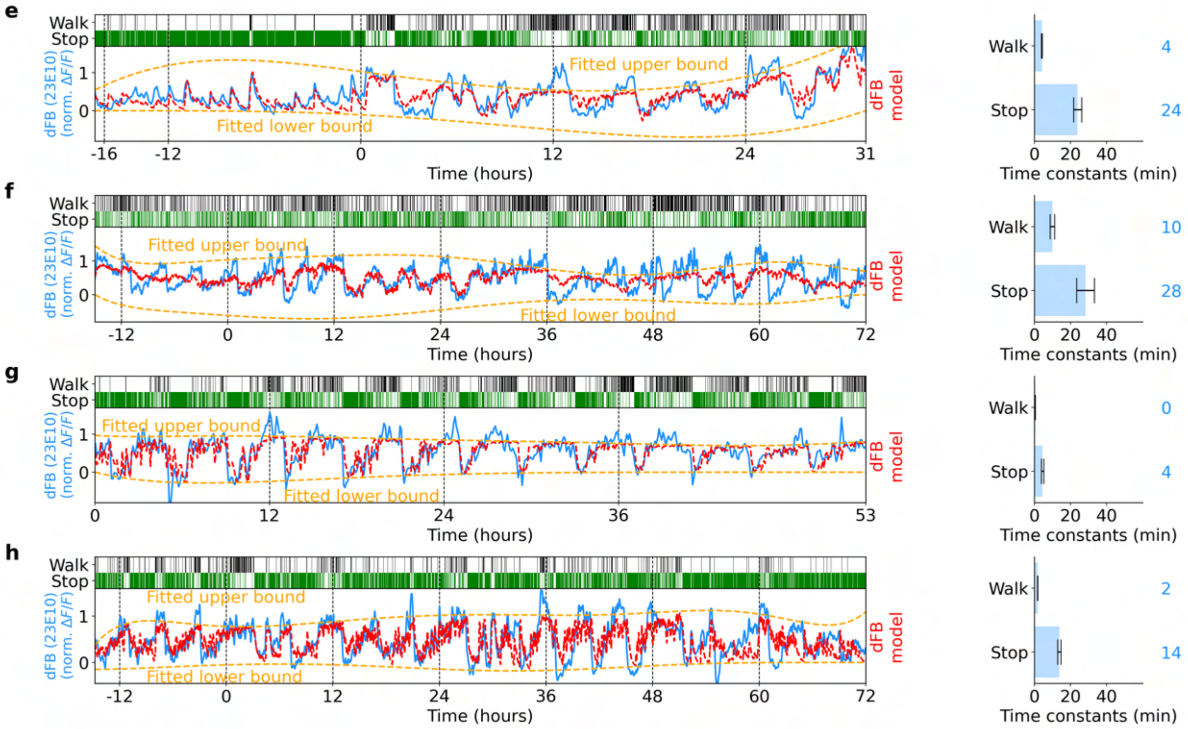

Supplementary Figure S43: Four more experiments and fits of the homeostat 2-state model with calcium activity of dFB neurons (labeled by 23E10-GAL4). **e-h** As in and continued from Supplementary Fig. S42.

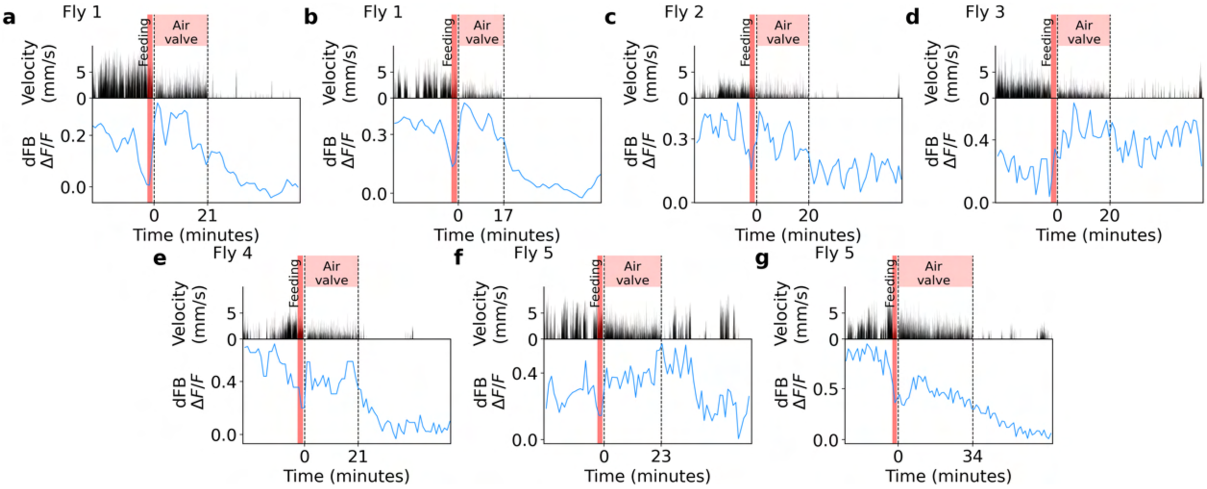

Supplementary Figure S44: Trials where air supply of the ball was intermittently interrupted to promote walking after feeding, while activity in dFB neurons was recorded. **a** First row: times where the ball was perturbed opening and closing the air valve intermittently which induced fast walking behavior (red region). Second row: velocity of the fly. Third row: Calcium activity in dFB neurons. Vertical red line indicates feeding event before perturbing the ball. **b-g** Same as a. Each panel represents a different trial. The fly from which each trial was recorded is shown in the top left corner of each panel. See Extended Data Fig. 1f for ROIs of dFB neurons. See Supplementary Fig. S45 for comparison.

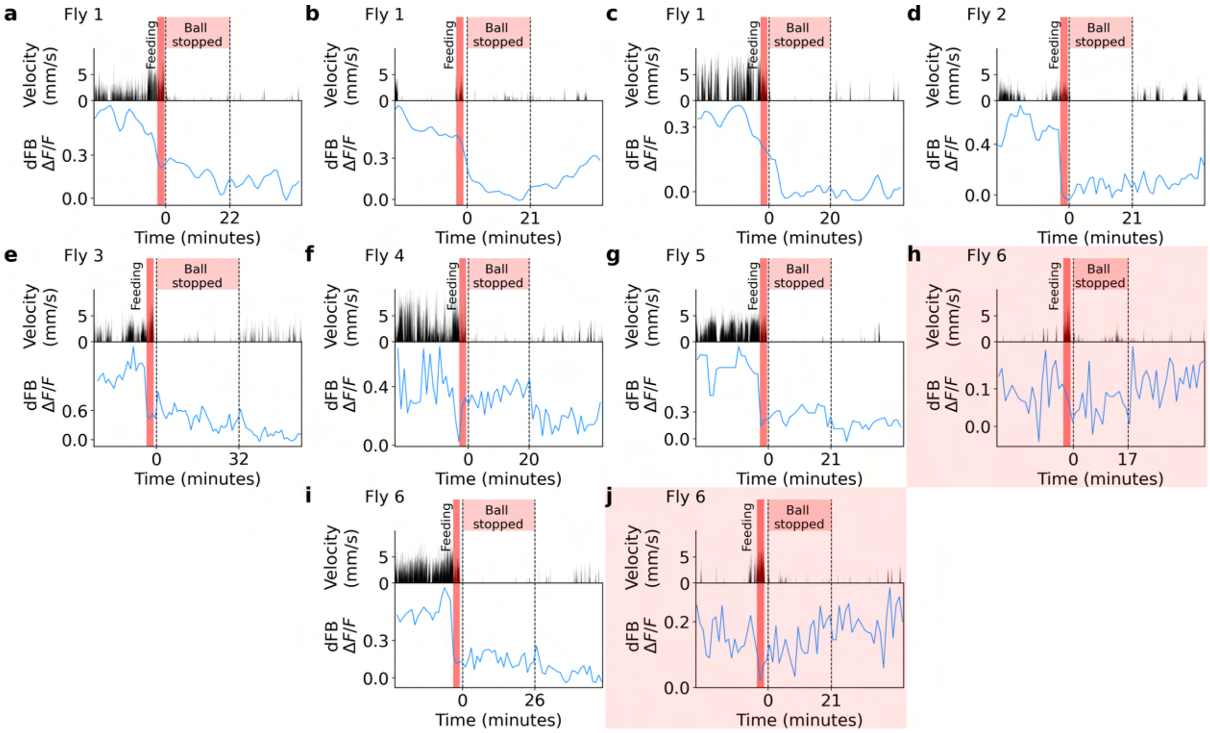

Supplementary Figure S45: Trials where the ball was blocked after feeding while activity in dFB neurons was recorded. **a** First row: time where the ball was stopped (red region). This induced constant pushing and pulling on the ball without coordinated walking activity, see Methods. Second row: velocity of the fly. Third row: Calcium activity dFB neurons. The vertical red line indicates the feeding event before blocking the ball. **b-j** Same as a. Each panel represents a different trial. Trials h and j were not considered for the analysis (highlighted in red) because dFB neurons had low activity levels before feeding. The fly from which each trial was recorded is shown in the top left corner of each panel. See Extended Data Fig. 1f for ROIs of dFB neurons. See Supplementary Fig. S44 for comparison.

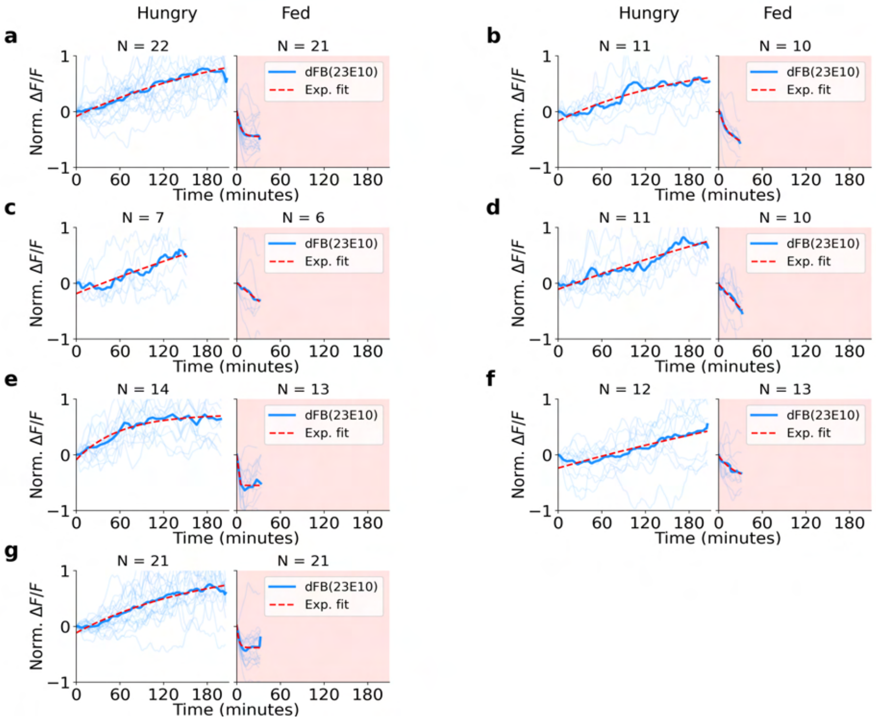

Supplementary Figure S46: Normalized fluorescence traces during hungry and fed epochs for seven flies in dFB neurons (labeled by 23E10-GAL4). **a** Left side: single (thin lines) and average (thick lines) normalized fluorescence traces of activity in dFB neurons during hungry epochs. Red lines indicate exponential fit. Right side: same as the left side, but during fed epochs. **b-g** Same as a. Each panel is from a different fly corresponding to Supplementary Figs. S38, S39.

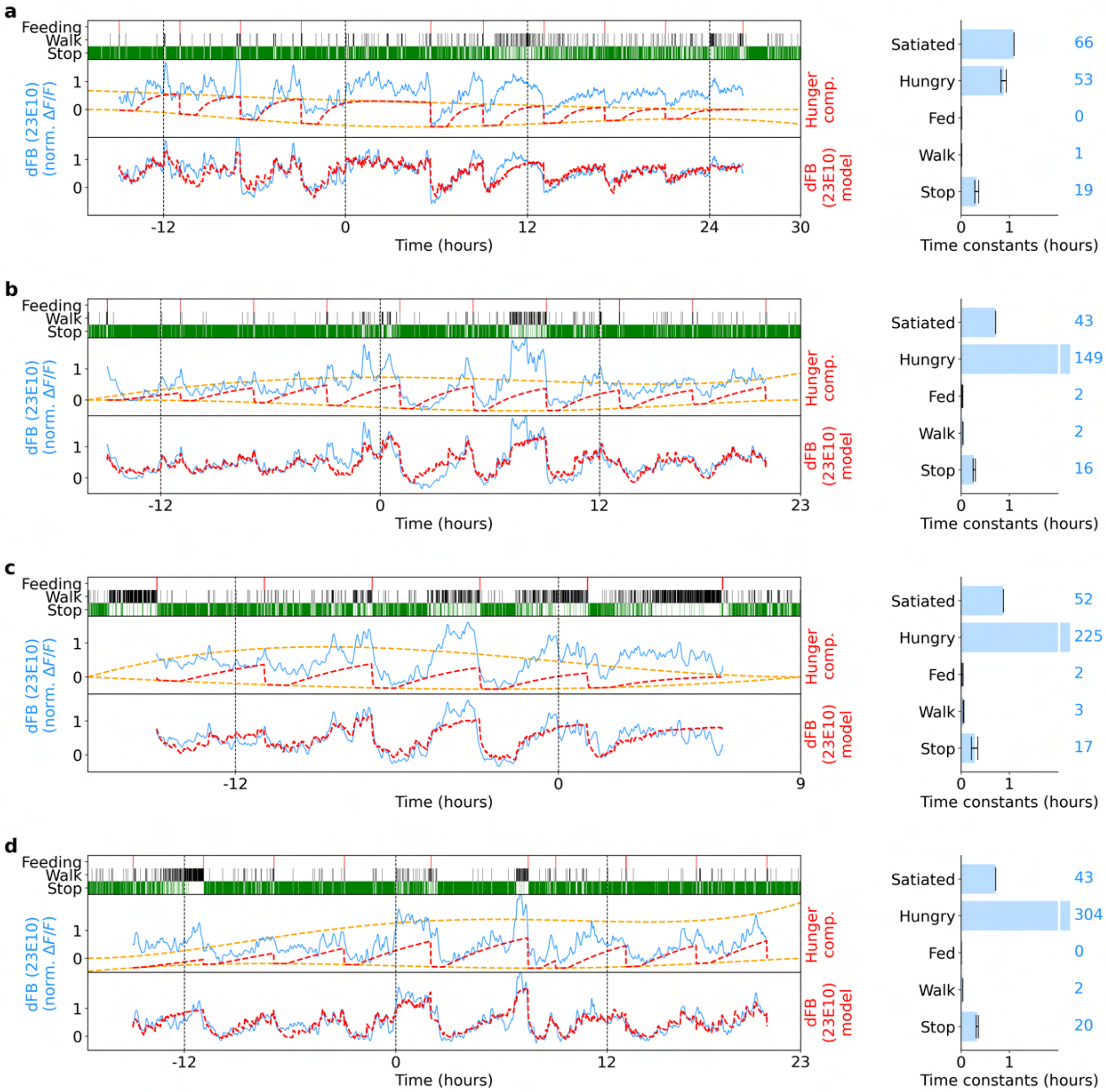

Supplementary Figure S47: Fitting calcium activity of dFB neurons (labeled by 23E10-GAL4) with hunger-walk model. **a** Left side: top row shows feeding events as well as walk and stop bouts of a fly. Second row: normalized fluorescence of dFB neurons in blue. Red line shows hunger component fitted by the model, while orange lines represent fitted upper and lower bounds of the hunger component. Third row: normalized fluorescence of dFB neurons in blue and fitted hunger-walk model in red. Right side: fitted time constants from the model. Gray lines indicate error bars of estimated time constants (see Methods), while colored numbers show their rounded fitted value. **b-d** Same as a. Each panel represents a fitted model for a different fly.

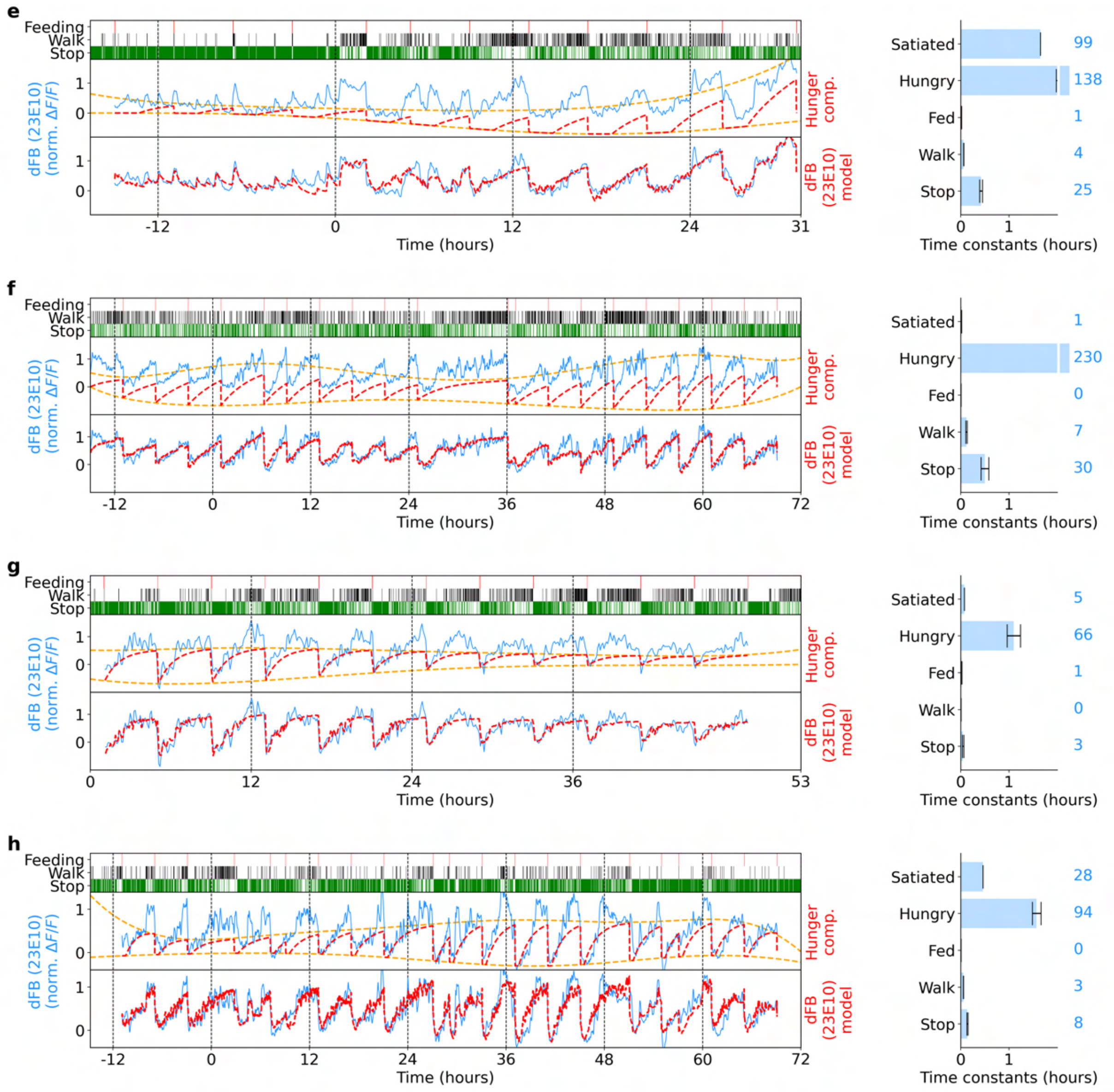

Supplementary Figure S48: Another four experiments (flies) with dFB neurons and fits of hunger-walk model to calcium activity (labeled by 23E10-GAL4). **e-h** As in and continued from Supplementary Fig. S47. See Extended Data Fig. 1f for ROIs of dFB neurons.

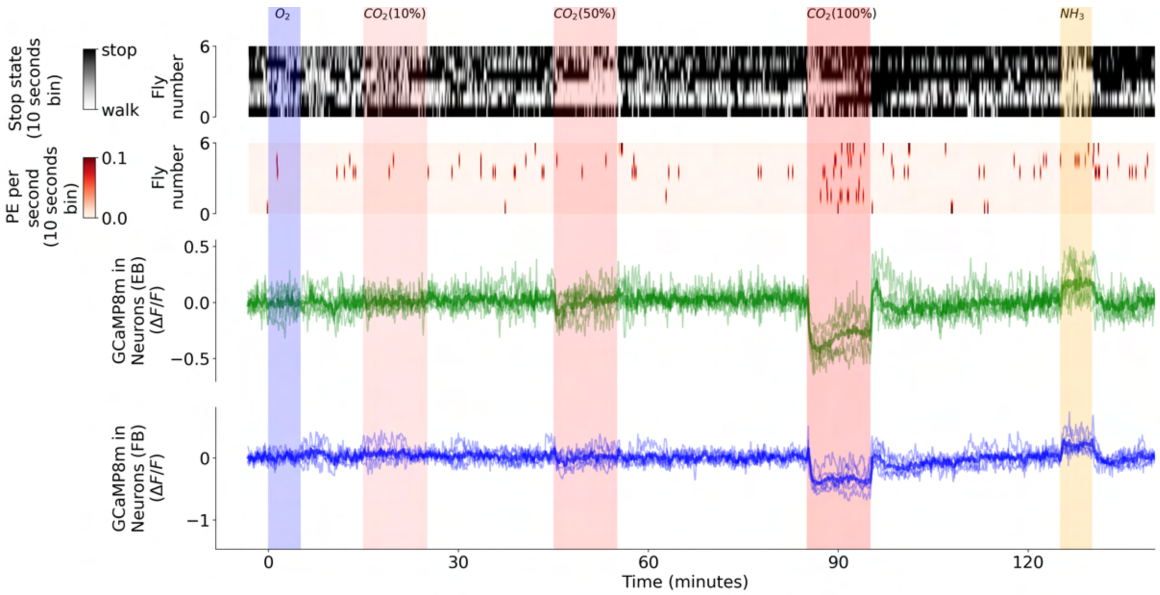

Supplementary Figure S49: Single traces of combined gas exposure during imaging experiments in neurons expressing GCaMP8m (UAS-jGCaMP8m;57C10-GAL4). First row: type of gas exposure. Periods of airflow between gas exposures are not shown. Second row: stop state (black) of 6 different flies, calculated in 10-s bins. Third row: proboscis extensions per second, calculated in 10-s bins. Forth and fifth rows: traces corresponding to single recordings of GCaMP8m in EB and FB, respectively. Average is shown as a thick line.

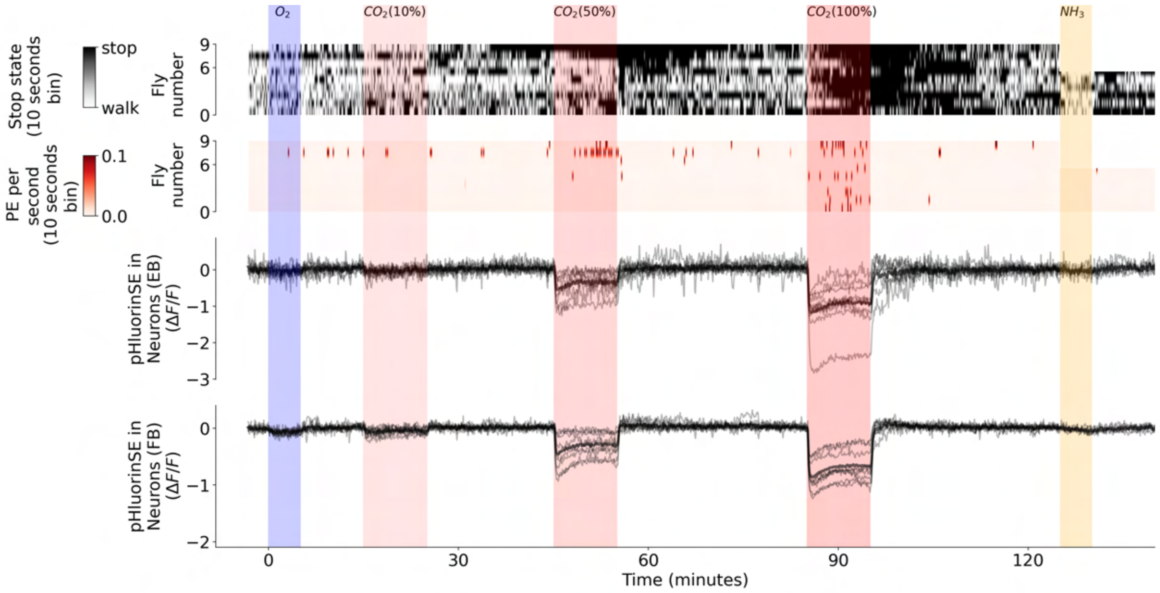

Supplementary Figure S50: Same as S49 but for neurons expressing pHluorinSE (UAS-pHluorinSE;57C10-GAL4). Recorded from 9 individual flies, 6 of which were exposed to ammonia (indicated in the y-axis of second and third rows).

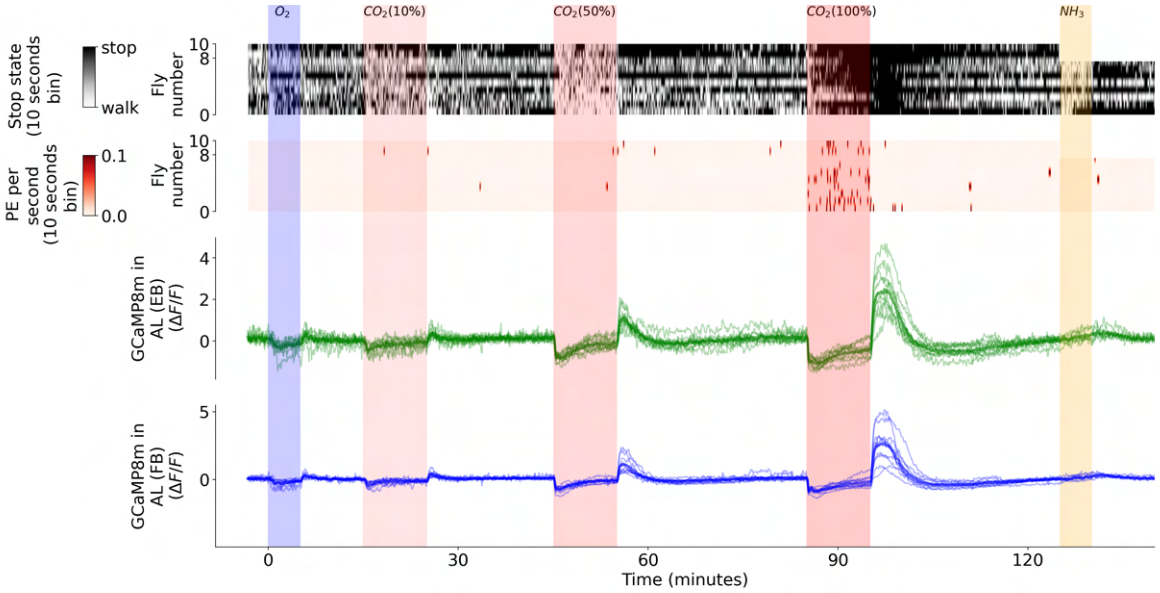

Supplementary Figure S51: Same as S49 but for astroctyes (AL) expressing GCaMP8m (UAS-jGCaMP8m;86E01-GAL4). Recorded from 10 individual flies, 8 of which were exposed to ammonia (indicated in the y-axis of second and third rows).

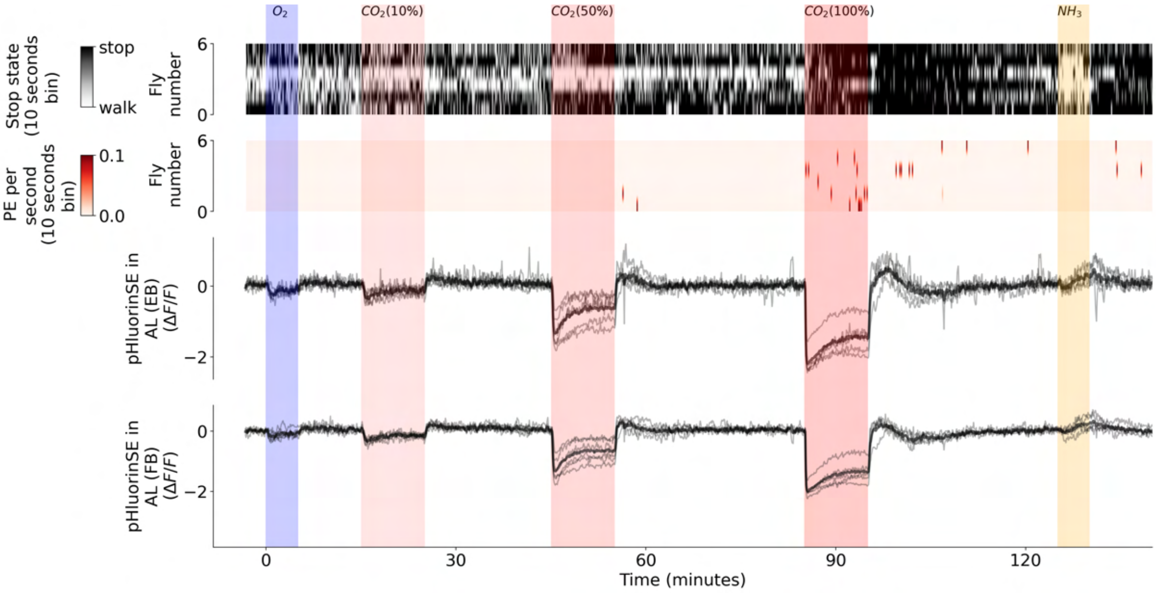

Supplementary Figure S52: Same as S49 but for astroctyes (AL) expressing pHluorinSE (UAS-pHluorinSE;86E01-GAL4).

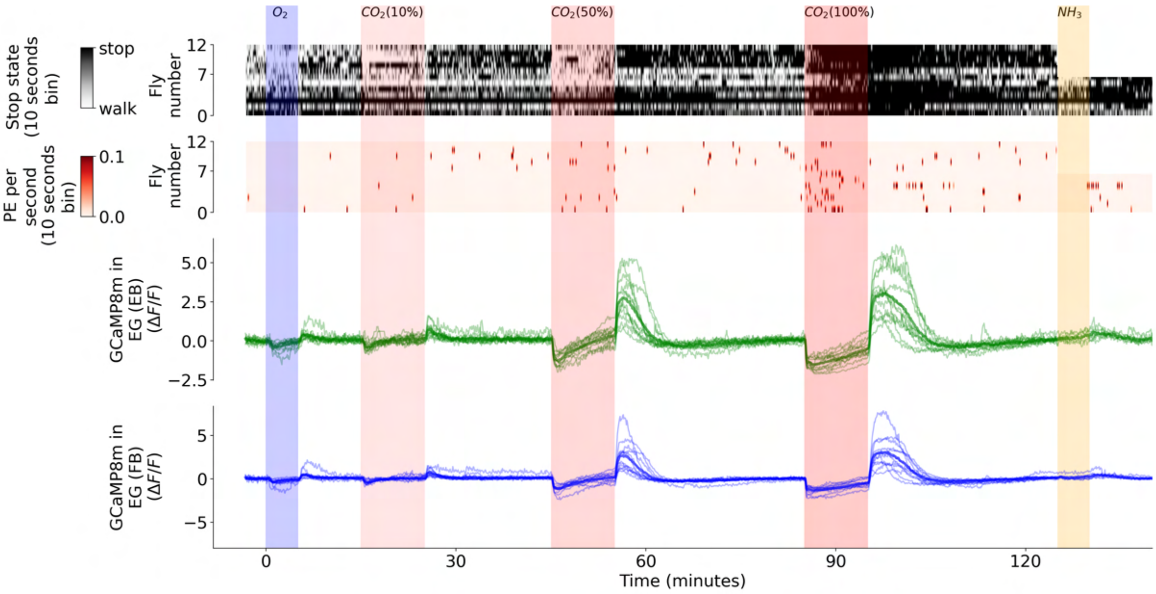

Supplementary Figure S53: Same as S49 but for ensheathing glia (EG) expressing GCaMP8m (UAS-jGCaMP8m;56F03-GAL4). Recorded from 12 individual flies, 7 of which were exposed to ammonia (indicated in the y-axis of second and third rows).

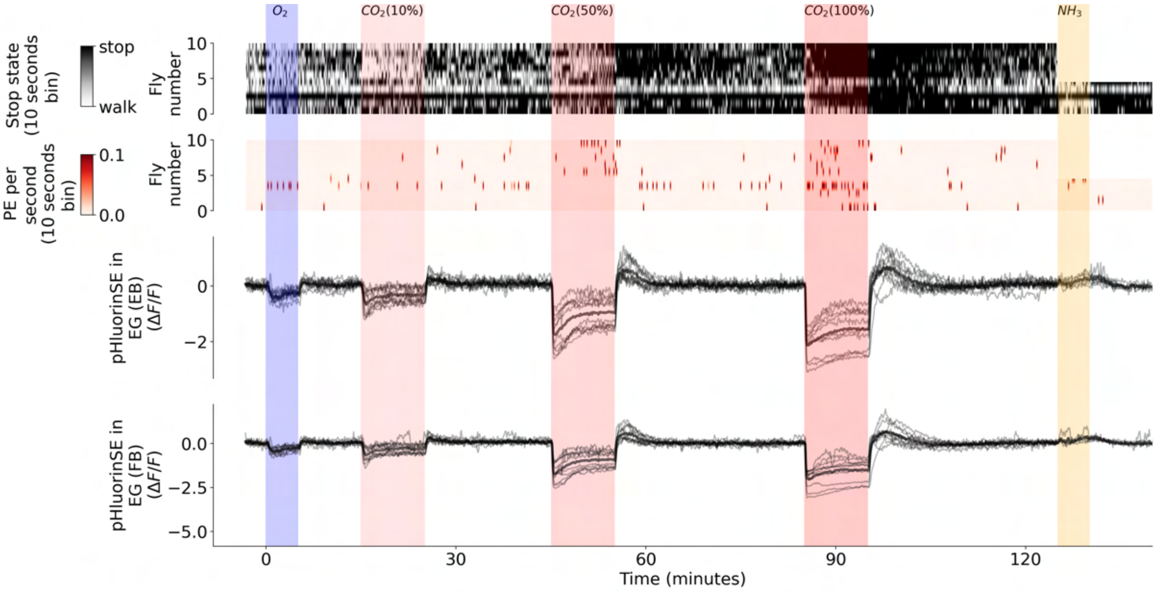

Supplementary Figure S54: Same as S49 but for ensheathing glia (EG) expressing pHluorinSE (UAS-pHluorinSE;56F03-GAL4). Recorded from 10 individual flies, 5 of which were exposed to ammonia (indicated in the y-axis of second and third rows).

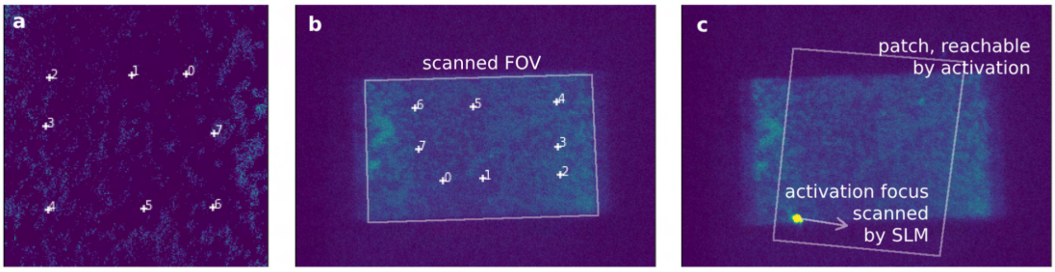

Supplementary Figure S55: Images of fluorescent beads used for optogenetic activation calibration: **a** 2-photon image of the beads. **b** Area camera view of the sample, illuminated by scanning beam (the exposure of the camera is longer than scanning period). **c** Area camera view of the scanned sample, with optogenetic laser focused in one spot. Calibration procedure: 2-photon images (a) are taken simultaneously with the area scan camera image (b), and correspondence of several prominent features in both images are selected manually. Transform matrix is found between pixel coordinates of the 2-photon and area camera image. Next, k-space is sampled with SLM by displaying phase ramps with varying spatial frequencies, sampling the k-space in x- and y-directions from -0.5 to 0.5 inverse pixels. Corresponding foci locations are found in area camera images (c), and the second transform matrix is calculated, linking area camera pixels to the SLM coordinates. The bounds of the patch, illuminated by the SLM are found. The final transform matrix is obtained by chaining both found transforms, linking 2-photon image coordinates to the coordinates of the optogenetic activation illumination patch. This transform is applied to any activation pattern drawn on 2-photon image, to obtain activation pattern in coordinates linked to the SLM, thus allowing to run Gerchberg-Saxton algorithm on it and yield the corresponding phase hologram.

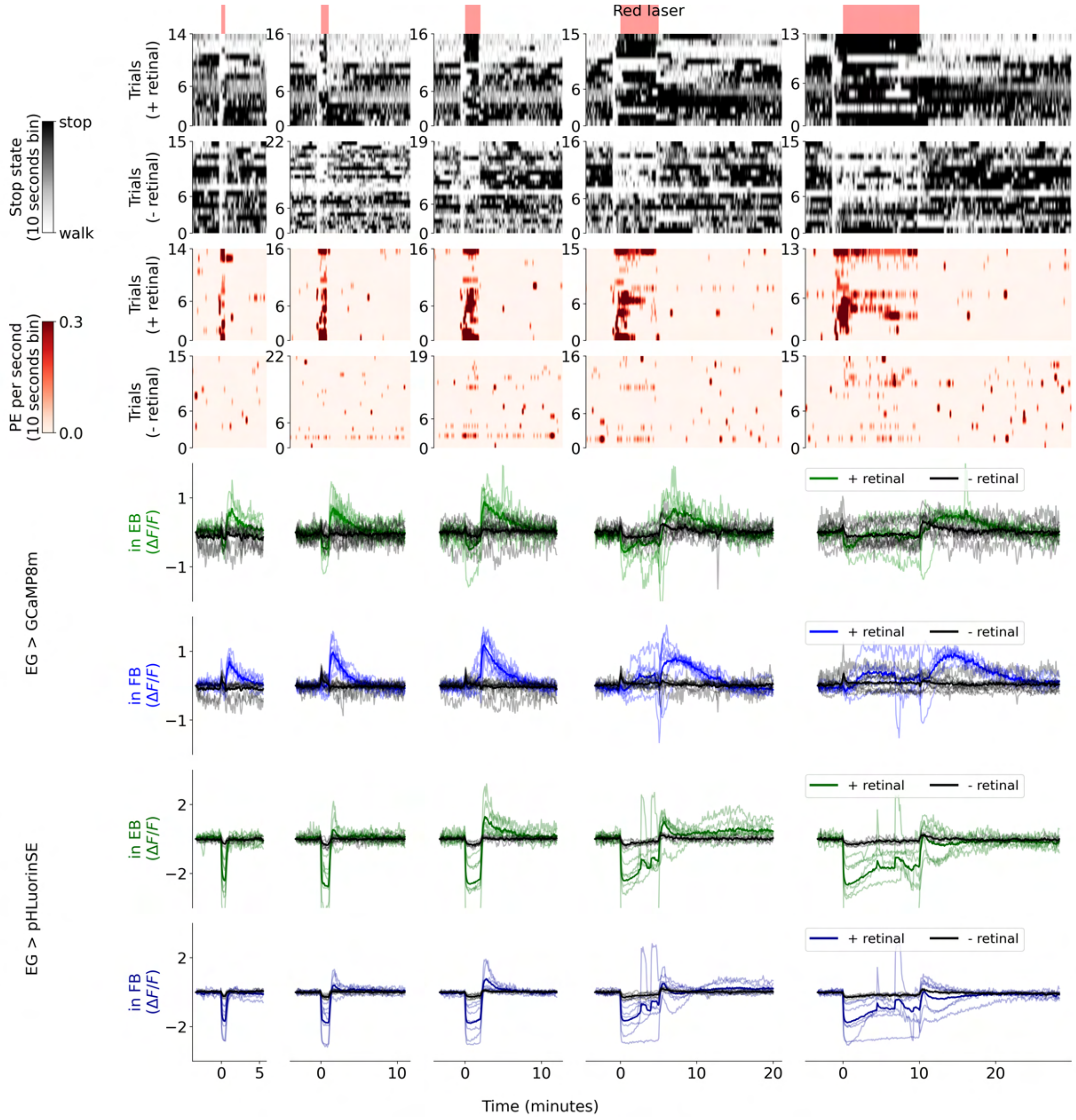

Supplementary Figure S56: Single traces of combined optogenetics and imaging experiments in ensheathing glia (EG). First row: laser activation. Second row: Stop state (black) of different trials for $n = 12$ flies fed with retinal food. The number of trials varied across activation times, as certain activation times were repeated in some flies (see Methods for details). Third row: Stop state for flies fed with standard food. Forth row: Proboscis extension per second in flies fed with retinal food. Fifth row: proboscis extension per second in flies fed with standard food. All flies expressed CsChrimson and either pHluorinSE (6 flies) or jGCaMP8m (6 flies). Trials 0–6 correspond to flies expressing pHluorinSE, while trials 7 onward involve flies expressing jGCaMP8m. Sixth and seventh rows: traces corresponding to single recordings of jGCaMP8m in EB and FB, respectively (from trials 7 onward). Average is shown as a thick line. Eighth and ninth rows: traces corresponding to single recordings of pHluorinSE in EB and FB, respectively (from trials 0-6).

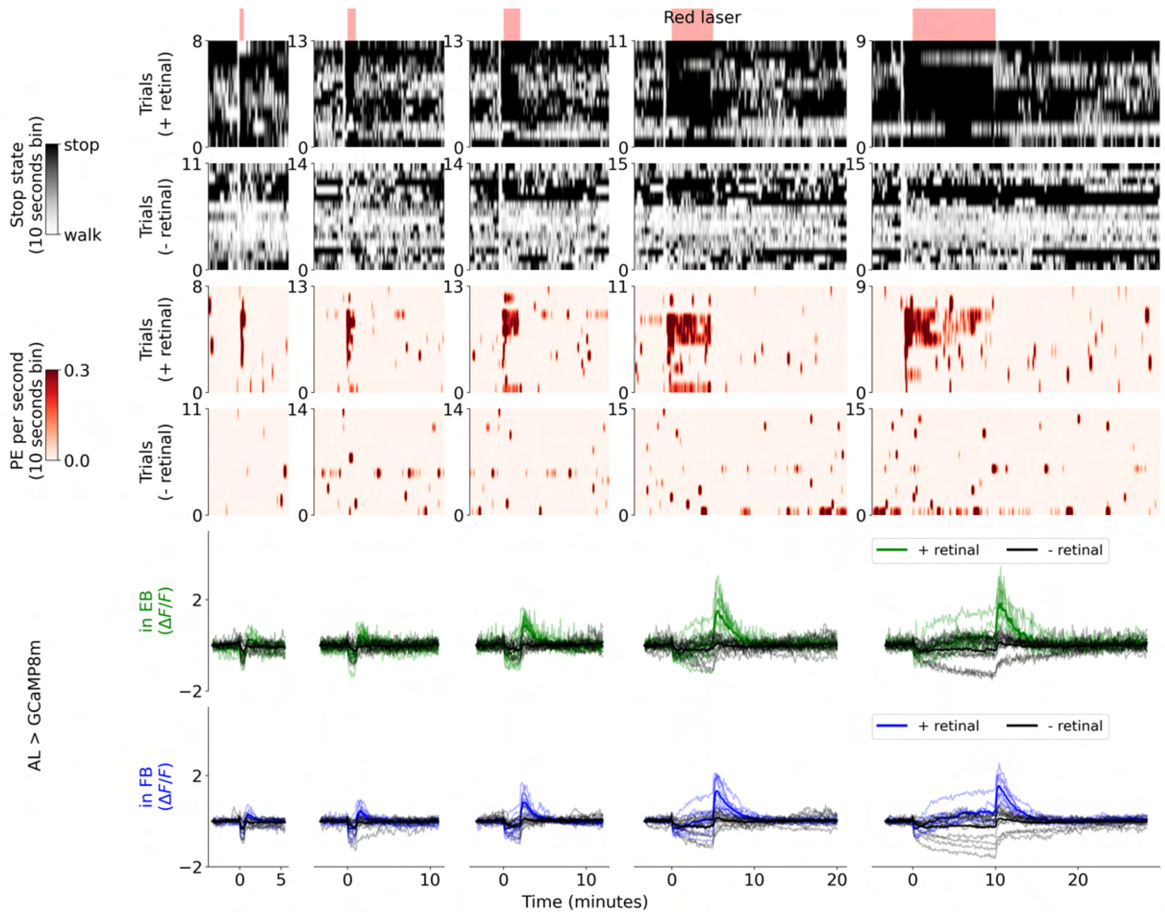

Supplementary Figure S57: Single traces from optogenetics experiments in astrocytes (AL), similar to Supplementary Fig. S56. Trials were recorded from a total n= 8 flies fed with retinal food, and n= 8 flies fed with standard food (control).

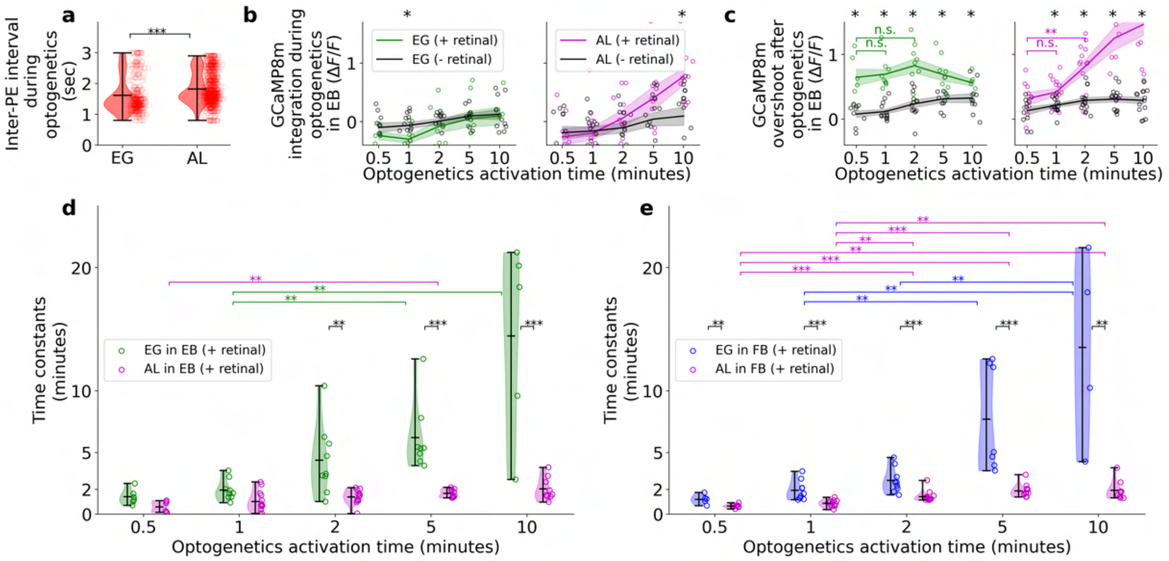

Supplementary Figure S58: Analysis of proboscis extension (PE) behavior and glia dynamics during optogenetics activation. **a** Time between consecutive PEs (period) during optogenetics activation of EG ($n = 439$ points) and AL ($n = 1251$ points). Statistical significance was extracted using the Kolmogorov–Smirnov test ($p < 0.0005$, for detailed statistics information, see Supplementary Data S1). **b** and **c** same as 7f and g, but for EB. Color code in c same as in b. **d** Fitted time constants from the calcium decay in EG (green) and AL (magenta) after each optogenetic activation experiment in EB. **e** Same as d but for FB where EG is now in blue. Asterisks indicate statistical significance using the t-test between EG and AL (black), and between different optogenetics activation times in EG (green) and AL (magenta). The number of data points in figures b-e for each group and for each optogenetics activation time is shown in Supplementary Fig. S56 (for EG) and S57 (for AL). One, two, or three asterisks indicate statistical significance for $p < 0.05$, $p < 0.005$, or $p < 0.0005$, respectively. All p-values were corrected using Benjamini–Hochberg procedure (for detailed statistics see Supplementary Data S1).

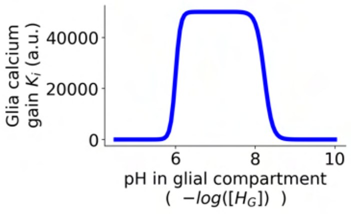

Supplementary Figure S59: The glia controller model gain, K_i, is expressed as a function of glial pH. This relationship is used to simulate calcium controller partial inhibition during simulations of gas exposure or optogenetic activation (see Fig. 8d and f). The function is manually defined using two distinct sigmoid functions to allow smooth changes of the gain as pH changes (equation (30)).

Supplementary Data S1: Statistical data for all figures

Supplementary Video S1: Video for experiment of fly 1. Top: time during experiment. Middle, right: imaging data (60 frames or 1 s average, recorded every 1 min). Rotating blue (during the day) or gray (during the night) arch represents stripe orientation in VR with respect to the fly (the fly's head and abdomen are pointing toward the upper and lower axis of the video, respectively). Right: side view of the fly on the ball. Behavior classification is shown in white in the left corner. Bottom panel shows velocity of the fly (first row), behavior classification (second row), and glia activity in EB (green) and FB (blue) over time. Gray areas in top and bottom rows indicate night (VR display is off). The time during the experiment at which the movie snippet was extracted is indicated by the green bar.

Supplementary Video S2: Same as Supplementary Video S1 for fly 2.

Supplementary Video S3: Same as Supplementary Video S1 for fly 3.

Supplementary Video S4: Same as Supplementary Video S1 for fly 4.

Supplementary Video S5: Same as Supplementary Video S1 for fly 5.

Supplementary Video S6: Same as Supplementary Video S1 for fly 6.

Supplementary Video S7: Classification of fly behavior using 3D CNN. The video shows examples of classification of different behaviors.

Supplementary Video S8: Example of tracking freely walking flies in behavior setup under infrared illumination.
